# Big data mining, rational modification, and ancestral sequence reconstruction inferred multiple xylose isomerases for biorefinery

**DOI:** 10.1126/sciadv.add8835

**Published:** 2023-02-01

**Authors:** Sitong Chen, Zhaoxian Xu, Boning Ding, Yuwei Zhang, Shuangmei Liu, Chenggu Cai, Muzi Li, Bruce E. Dale, Mingjie Jin

**Affiliations:** ^1^ School of Environmental and Biological Engineering, Nanjing University of Science and Technology, Nanjing 210094, China.; ^2^ Biorefinery Research Institution, Nanjing University of Science and Technology, Nanjing 210094, China.; ^3^ Biomass Conversion Research Laboratory, Department of Chemical Engineering and Materials Science, Michigan State University, East Lansing, MI 48824, USA.; ^4^ Great Lakes Bioenergy Research Centre (GLBRC), Michigan State University, East Lansing, MI, 48824 USA.

## Abstract

The isomerization of xylose to xylulose is considered the most promising approach to initiate xylose bioconversion. Here, phylogeny-guided big data mining, rational modification, and ancestral sequence reconstruction strategies were implemented to explore new active xylose isomerases (XIs) for *Saccharomyces cerevisiae*. Significantly, 13 new active XIs for *S. cerevisiae* were mined or artificially created. Moreover, the importance of the amino-terminal fragment for maintaining basic XI activity was demonstrated. With the mined XIs, four efficient xylose-utilizing *S. cerevisiae* were constructed and evolved, among which the strain *S. cerevisiae* CRD5HS contributed to ethanol titers as high as 85.95 and 94.76 g/liter from pretreated corn stover and corn cob, respectively, without detoxifying or washing pretreated biomass. Potential genetic targets obtained from adaptive laboratory evolution were further analyzed by sequencing the high-performance strains. The combined XI mining methods described here provide practical references for mining other scarce and valuable enzymes.

## Teaser

Combined data mining, rational modification, and ancestral sequence reconstruction results in the discovery of xylose isomerase.

## INTRODUCTION

With the increasing demand for energy supply, rapid consumption of fossil resources, and requirement for low carbon emissions, lignocellulosic biorefinery has attracted much interest (*1*–*3*). Lignocellulosic biomass mainly consists of cellulose, hemicellulose, and lignin, which account for approximately 32 to 52, 16 to 33, and 9 to 32 weight % (wt %), respectively, across different plants (*4*). With developed biorefinery technologies, cellulose and hemicellulose can be efficiently hydrolyzed to fermentable sugars, mostly glucose and xylose. Lignocellulosic biomass–derived glucose has been further efficiently converted to multiple chemicals, such as alcohols, organic acids, amino acids, and aromatic compounds, by various microbial strains (*5*, *6*). However, the technology for the bioconversion of xylose to desirable products has progressed slowly due to limited efficient xylose-fermenting microbes. In particular, *Saccharomyces cerevisiae*, one of the most suitable microorganisms for lignocellulosic biorefineries, cannot use xylose. Given the high hemicellulose content in lignocellulosic biomass (even up to 50 wt % in certain grasses and cereal tissues) (*4*, *7*, *8*), efficient conversion of xylose is significant for lignocellulosic biorefinery.

Three main types of xylose metabolic pathways have been identified in nature: the oxido-reduction pathway, isomerization pathway, and oxidative pathways of Weimberg and Dahms (*9*–*14*), among which the first two types have been studied the most. The oxido-reduction pathway, with xylose reductase (XR) and xylitol dehydrogenase (XDH) as key enzymes, is mostly found in fungi, such as *Scheffersomyces* (*Pichia*) *stipitis*. In these strains, xylose is first reduced to xylitol by NADPH (reduced form of nicotinamide adenine dinucleotide phosphate)–preferred XR, and then the generated xylitol is oxidized to xylulose for further metabolism by nicotinamide adenine dinucleotide–dependent XDH. The xylose isomerization pathway, with xylose isomerase (XI) as the key enzyme, mainly exists in bacteria and a few fungi, which are capable of directly isomerizing xylose to xylulose independent of cofactors. Nonetheless, many important industrial microorganisms, such as *S. cerevisiae*, do not express sufficient amount of enzymes to convert xylose efficiently to xylulose, although they have complete xylulose metabolism systems (*15*). When XRs-XDHs and/or XIs are actively expressed in these microorganisms, xylose can be efficiently converted to xylulose (*16*, *17*), which is further converted to xylulose 5-phosphate by xylulose kinase and enters the nonoxidative pentose phosphate pathway, glycolytic pathway, tricarboxylic acid cycle, etc., enabling the synthesis of substantial amount of metabolites/bioproducts (*18*–*21*). However, the cofactor imbalance of the oxido-reduction pathway typically leads to the accumulation of xylitol as a byproduct, reducing metabolic fluxes to downstream pathways. Although numerous efforts have been made to regulate intracellular cofactor status (e.g., modifying the cofactor specificity of XRs and/or XDHs, regulating the mutual conversion of reduced form of nicotinamide adenine dinucleotide and NADPH), the intracellular cofactor balance is difficult because there are many correlative intracellular reactions (*22*–*24*). Cofactor-independent XIs are considered the most promising enzymes for xylose valorization as they entirely circumvent the cofactor imbalance in oxido-reduction pathways. Unfortunately, few XIs are available for use in industrial microorganisms.

*S. cerevisiae* is one of the most preferred microorganisms for lignocellulosic biorefineries and the most widely used microorganism in the current fermentation field due to its safety, robustness, metabolic versatility, and insensitivity to bacteriophage contamination (*21*, *25*, *26*). In addition, bioethanol produced by *S. cerevisiae* is regarded as the first large-scale product obtained from lignocellulosic biomass (*1*, *3*, *5*). In 1996, an XI (TheXI) from *Thermus thermophilus* was first actively expressed in *S. cerevisiae*, breaking the perspective that XIs could not function in *S. cerevisiae* due to possible protein misfolding, improper posttranslational modification, incorrect disulfide formation, unmatched intracellular pH, etc. However, the optimum temperature for TheXI is 85°C, and only 4% activity is retained at 30°C, limiting its further application in fermentation processes (*27*). Seven years later, Kuyper *et al.* (*28*) reported the high-level functional expression of an XI (PirXI) from the anaerobic fungus *Piromyces* sp. E2 in *S. cerevisiae*. Thereafter, researchers have searched for XIs active in *S. cerevisiae* for several decades across strains, environments, and metagenomes, but most attempts have met with disappointing results, and only approximately 20 active XIs have been identified to date (*29*–*32*). Moreover, the activity of these XIs in *S. cerevisiae* varies greatly, with only very few XIs, such as PirXI from *Piromyces* sp. E2, exhibiting high activity and being applied for efficient xylose-utilizing *S. cerevisiae* construction. In light of the significant economic implications of xylose utilization, it is important to find more active XIs and explore the factors affecting their activity in *S. cerevisiae*.

Benefiting from rapidly developing high-throughput sequencing technologies, massive nucleotide/protein data from diverse samples have been sequenced and uploaded to public databases. With appropriate approaches, numerous efficient enzymes can be mined from these meta-data, providing high-quality enzymes for biocatalysis and metabolic engineering. For instance, Ozcan *et al.* (*33*) mined several Cas7-11 proteins free of collateral activity and cell toxicity from a meta genomic database by using verified Cas7-11 family members as references; Hillenmeyer *et al.* (*34*) constructed a large-scale phylogenetic tree to analyze type II polyketide gene clusters and traced the evolutionary history from a single ancestor to current gene clusters; and in a recent study, the third triterpene synthase was mined through an AlphaFold2-based genome mining approach on the basis of understanding the catalytic mechanism of the first two triterpene synthases (*35*). Likewise, immense amounts of data on XIs are available, making it possible to trace their evolutionary footprints, to analyze the differences between active and inactive XIs in *S. cerevisiae* and to mine highly active XIs for lignocellulosic biorefineries from untapped resources.

**Fig. 1.**
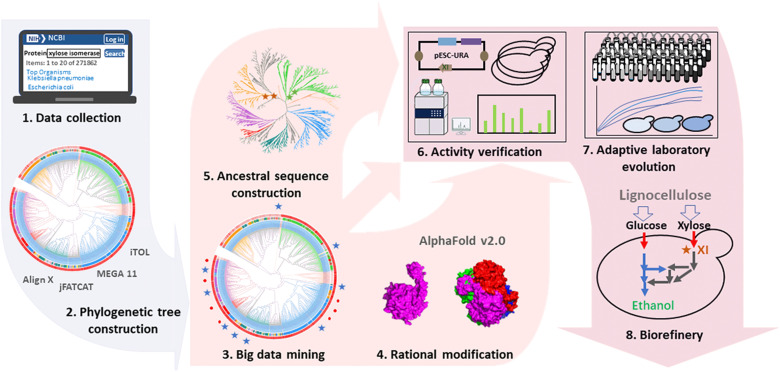
Big data mining, rational modification, and ancestral sequence reconstruction inferred multiple active XIs for biorefinery.

There were approximately 250,000 annotated XIs publicly available in the NCBI (National Center for Biotechnology Information) database on 10 November 2019, among which approximately 240,000 sequences are from bacteria, and the rest are from fungi and archaea. In this study, after reducing redundant sequences, we used 867 representative XI sequences and 16 verified XIs active in *S. cerevisiae* to construct a phylogenetic tree. Then, 15 uncharacterized XIs were selected from the phylogenetic tree for activity testing in *S. cerevisiae*, and 4 exhibited xylose conversion capacity. Through sequence and structure analysis for active and inactive XIs in *S. cerevisiae*, we hypothesized that the shortened or extended N-terminal fragments were one of the main causes of XI inactivity in *S. cerevisiae*. This hypothesis was then confirmed as we successfully conferred activity on five XIs that were inactive in *S. cerevisiae* by complementing missed N-terminal fragments or deleting redundant N-terminal fragments and deactivated functional XIs by deleting N-terminal fragments or assembling extra N-terminal fragments. Moreover, we created four ancestral sequences for XIs, all of which exhibited activity in *S. cerevisiae*. The heterologous expression of the four initially mined active XIs enabled *S. cerevisiae* to produce ethanol from xylose. After adaptive laboratory evolution (ALE), the evolved *S. cerevisiae* strains showed excellent xylose fermentation performance in semisynthetic medium, corn stover hydrolysate, and corn cob hydrolysate (Fig. 1). The 13 active XIs, which were directly mined from databases, revived from inactive ones, and artificially created through computer reconstruction, and the xylose-utilizing *S. cerevisiae* strains constructed in this study will facilitate the valorization of lignocellulosic biomass, especially the hemicellulose component. The XI mining methods mentioned here also provide practical references for finding other scarce and valuable enzymes for biotechnology.

## RESULTS AND DISCUSSION

### Phylogenetic analysis of XIs from the NCBI database

Researchers have been seeking functional XIs in *S. cerevisiae* for more than four decades. Despite enormous efforts, only approximately 20 XIs active in *S. cerevisiae* have been obtained (*29*, *32*). Finding highly active XIs in *S. cerevisiae* is critical for lignocellulosic biorefinery and for broadening our knowledge of XIs. Excitingly, the tremendous amounts of data from high-throughput sequencing provide an opportunity for mining active XIs. According to the search results, there were approximately 250,000 amino acid sequences relevant to XIs in the NCBI database on 10 November 2019, a potential source of treasure for mining.

**Fig. 2.**
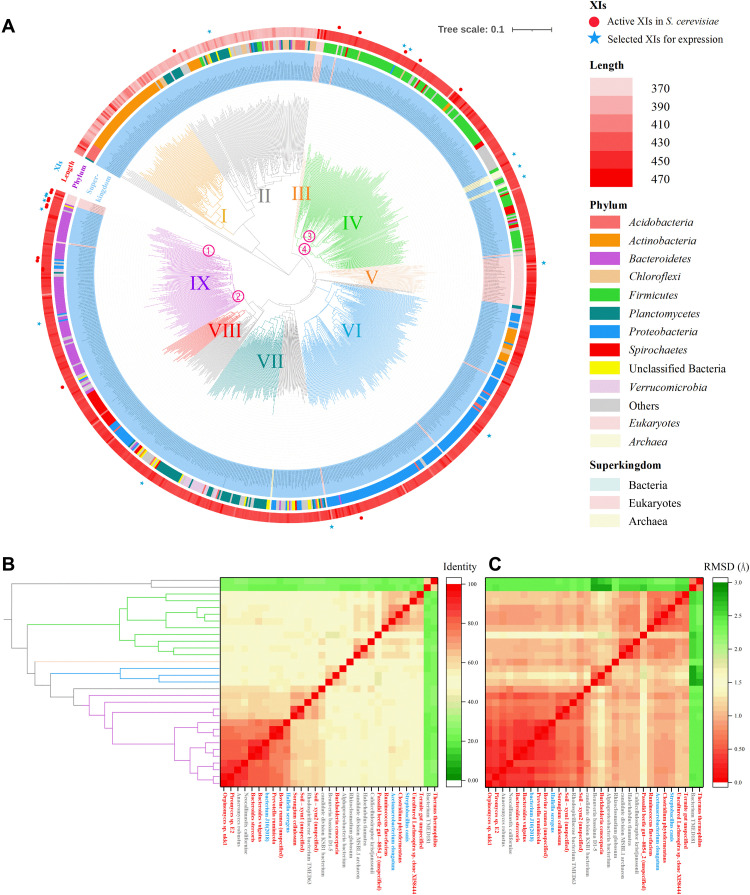
Evolutionary analysis of XIs on the basis of reported XIs and annotated XIs in a public database. (**A**) A phylogenetic tree constructed on the basis of disclosed XIs and annotated XIs in the NCBI database. As illustrated, the phylogenetic tree mainly consists of nine evolutionary branches, which are represented in different colors. The accession numbers and original hosts of XIs are shown at the ends of branches. The superkingdoms of the corresponding hosts are marked via the background colors under XI labels. Phyla are labeled using color strips located outside the circular phylogenetic tree. Sequence lengths are represented by color gradients. The 16 reported XIs that are active in *S. cerevisiae* are labeled with red circles, and 15 tested XIs in this study are labeled with blue stars. (**B**) The identities of the 16 disclosed XIs (labeled in red) and 15 tested XIs (the active ones are labeled in blue and the inactive ones are labeled in gray). (**C**) Root mean square deviations (RMSDs) of tertiary structures of 16 disclosed active XIs (labeled in red) and 15 XIs (the active ones are labeled in blue and the inactive ones are labeled in gray) tested in this study.

We first excluded non-XIs from these 250,000 amino acid sequences based on the length (380 to 390 amino acids for type I XIs and 440 to 460 amino acids for type II XIs), obtaining approximately 40,000 sequences. Second, these XIs were further simplified by removing duplicate and redundant sequences using cluster analysis (redundancy threshold of 73% sequence identity), which resulted in 1042 sequences. Third, sequences with conserved sites (metal ion binding sites: H102, D105, E233, K235, E269, H272, D297, D308, D310, and D340; residues around the substrate binding pocket: W50, F61, W140, F146, and W189; coordinates are based on PirXI) (*32*) were further retained, leaving 867 sequences. These 867 putative XIs and 16 verified XIs active in *S. cerevisiae* (table S3) were selected to build a phylogenetic tree using the maximum likelihood method to depict the evolutionary history of XIs. As shown in Fig. 2A, the obtained phylogenetic tree was mainly grouped into nine major evolutionary branches: XIs in clades I, IV, VI, VII, VIII, and IX are mainly from the phyla *Actinobacteria*, *Firmicutes*, *Proteobacteria*, *Planctomycetes*, *Spirochaetes*, and *Bacteroidetes*; XIs in clades III and V are mainly from eukaryotes; and XIs in clade II are from multiple bacterial phyla. As referenced, *Proteobacteria*, *Firmicutes*, *Bacteroidetes*, and *Actinobacteria* are the most abundant phyla of natural bacteria (*36*), which suggests a relatively complete evolutionary history of the XIs derived from these phyla.

A few XIs that do not form distinct evolutionary branches and are distributed in multiple locations throughout the phylogenetic tree are likely derived from horizontal gene transfer. Six XIs from archaea with no distinct evolutionary branches might have undergone horizontal gene transfer from bacterial XIs. XIs from eukaryotes, accounting for approximately 4% of the total XIs, formed two independent evolutionary lineages (clades III and V). In particular, eukaryotic clades III and V are separated by the prokaryotic clade IV. We tend to attribute this phenomenon to horizontal gene transfer, which happened in the early stage of XI gene migration. The ancestor species in clade III and clade V received XI genes from prokaryotes and then underwent independent evolution. Although horizontal gene transfer commonly occurs in bacteria, it also occurs between prokaryote and eukaryotes. For instance, PirXI, the representative XI active in *S. cerevisiae*, is considered entering fungus *Piromyces* sp. E2 through horizontal gene transfer (*37*). A small branch of eukaryotes in clade IX probably acquired XI genes through horizontal transfer from *Bacteroidetes* and subsequently underwent independent evolution through gene mutation. XIs in clades I and II, mostly originating from *Actinobacteria*, *Planctomycetes*, *Chloroflexi*, and *Acidobacteria*, have substantially different evolutionary lengths and amino acid residue numbers compared with other XIs, suggesting that they belong to type I XIs. The remaining XIs with more than 400 amino acid residues were classified as type II XIs. The 16 reported XIs active in *S. cerevisiae* are mostly from *Firmicutes* (clade IV) and *Bacteroidetes* (clade IX), except that TheXI and BurXI are located in clades I and VI, respectively. PirXI, as well as other reported XIs from *Orpinomyces* sp. ukk1 (OrpXI), *Bacteroides stercoris* (BasXI), *Bacteroides vulgatus* (BavXI), *Prevotella ruminicola* (PreXI), and bovine rumen (unspecified) (BovXI), are close in evolutionary relationships.

### Mining XIs for active expression in *S. cerevisiae*

**Fig. 3.**
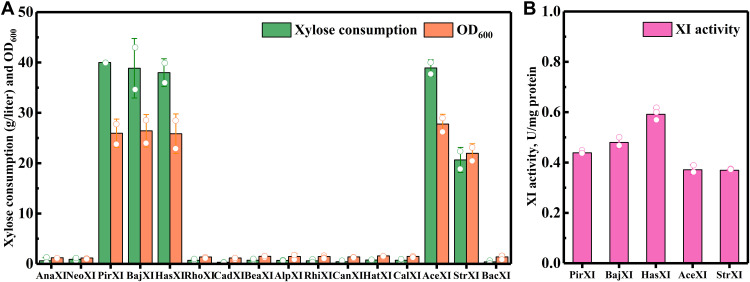
Activity test for 15 XIs mined from the public database. (**A**) The xylose consumption and cell growth of *S. cerevisiae* CRD3 expressing different XIs selected from the constructed phylogenetic tree, with PirXI as the positive control. The XIs were expressed in *S. cerevisiae* CRD3 by the replicating plasmid pESC-URA. The strains were cultured in YPX40 medium for 192 hours. As a result, the expression of PirXI, BajXI, HasXI, and AceXI all contributed to consumption up of xylose (40 g/liter). In contrast, the expression of StrXI contributed to xylose consumption (approximately 20 g/liter). (**B**) Enzyme activities of PirXI, BajXI, HasXI, AceXI, and StrXI when expressed in *S. cerevisiae* CRD3. One unit of XI activity was defined as the enzyme amount required to form 1 μM xylulose/min under the assay conditions. Considering that the xylulose generated in this system can be converted by the inherent xylulose kinase in crude enzyme solution, the obtained XI activities are lower than they actually are. The data shown in (A) and (B) are presented as the mean value ± SD of two biological replicates.

To mine XIs that are active in *S. cerevisiae*, 15 unidentified XIs (table S3) were chosen from the constructed phylogenetic tree, among which six XIs are evolutionarily close to the previously reported XIs, and the other nine XIs are distributed throughout the phylogenetic tree. The selected 15 XIs cover all the nine major evolutionary branches. After codon optimization, these XIs were separately expressed in a diploid strain *S. cerevisiae* CRD3 (an ATCC26603 derivative with overexpression of the nonoxidative pentose phosphate pathway and some other modifications beneficial to xylose utilization) using a replicating plasmid pESC-URA. Intriguingly, the introduction of BajXI (from Bacterium J10), HasXI (from *Hallella seregens*), AceXI (from *Acetanaerobacterium elongatum*), and StrXI (from *Streptobacillus canis*) enabled this *S. cerevisiae* to use xylose at 38.86, 37.97, 38.91, and 20.64 g/liter within 192 hours, with XI activities of protein at 0.48, 0.59, 0.37, and 0.37 U/mg, respectively (Fig. 3). We also expressed PirXI, the best XI so far, in CRD3 strain, which showed that the expression of HasXI, BajXI, or AceXI contributed to similar xylose utilization results with PirXI (Fig. 3A and fig. S5). This is the first report on the active expression of XIs from Bacterium J10, *H. seregens*, *A. elongatum*, and *S. canis*. While most verified XIs have been screened exclusively from lignocellulose-rich samples, such as the guts of wood-feeding insects, stomach contents of ruminants, and feces of herbivores, mining XIs from meta-data enlarged the sample sources of XIs. For instance, the *S. canis* strain mentioned here was isolated from a phlegmon in a dog, which is not rich in lignocellulose (*38*). Furthermore, the meta-data mining method also exhibits high potential in screening active XIs from uncultured microorganisms.

### Analysis of the sequence characteristics of active and inactive XIs

The earliest ancestral XIs, with similar sequences, evolved and diffused by mutations and horizontal gene transfers, leading to various survivors today. Most of them may harness the ability to convert xylose to xylulose in their native hosts, but when introduced into *S. cerevisiae*, the majority cannot function on xylose due to differences in the intracellular environment (pH, metal ions, temperature, etc.), protein synthetic modes, formation of tertiary structures, etc. (*39*). With the identified active XIs, inactive XIs, and the constructed phylogenetic tree, we expect to identify the key distinctions between active and inactive enzymes and investigate the key factors affecting XI activity.

Sequence alignments were performed for the 15 XIs tested in this study and the 16 reported active XIs. Basically, the identities of these 31 XIs exhibited a pattern consistent with the evolutionary relationships in the phylogenetic tree (Fig. 2B), with higher identities for XIs in the same branch (47 to 75% for branch IV and 55 to 95% for branch IX) and lower identities between different branches (≤57%). As illustrated in fig. S1, among the inactive XIs tested in this study, RhiXI and AnaXI lack a 37– and a 22–amino acid fragment in the N terminus, respectively, compared with active XIs. In contrast, there is an 11–amino acid extension in the N terminus of NeoXI. In addition, RhiXI features an extra fragment, “NTSIPFD,” in the middle of the sequence. In addition to the amino acid fragments, several individual amino acid sites of inactive XIs, as indicated by red boxes in fig. S1, were inconsistent with active XIs.

To deepen our understanding of the relationship between the structure and function of XIs, structure predictions were performed using AlphaFold v2.0 for the 15 XIs tested in this study and the 16 reported active XIs. With the experimentally measured PirXI structure as a positive control, the three-dimensional structure of PirXI predicted by AlphaFold v2.0 was remarkably similar to the experimentally measured structure, which both harness a distorted (β/α)8-barrel fold and a 3 α helix extension (*40*) with root mean square deviations (RMSDs) of 0.65 Å, demonstrating the accuracy of AlphaFold v2.0 for XI structure prediction. Basically, the three-dimensional structures of the abovementioned XIs varied with RMSD values, from 0.11 to 1.84 Å, except that TheXI and BacXI had a significant RMSD of 2.22 to 2.85 Å (Fig. 2C). The RMSDs for XIs in the same branches are relatively small (≤1.27 Å for branch IV and ≤0.77 Å for branch IX). In particular, the simulated structure showed that NeoXI contains an extra α helix at the N terminus compared with XIs active in *S. cerevisiae*, which is concordant with the 11–amino acid extension in the NeoXI N terminus (fig. S1). The N termini of both AnaXI and RhiXI are shorter than those of active XIs, although fragments in the N terminus of disclosed active XIs are not involved in secondary structure formation (fig. S2).

### Modifying inactive XIs for active expression in *S. cerevisiae*

**Fig. 4.**
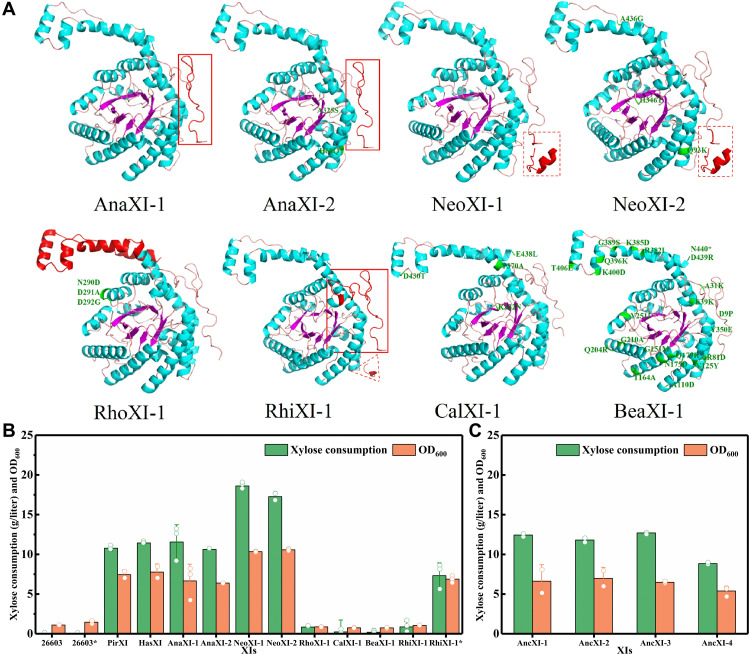
Modifying inactive XIs for active expression in *S. cerevisiae*. (**A**) Protein structure prediction of modified XIs using the AlphaFold v2.0 model. The protein structures formed by the altered amino acid sequence are marked in red. Specifically, AnaXI-1, AnaXI-2, and RhiXI-1 were modified by assembling a fragment to their N terminus; NeoXI-1, NeoXI-2, and RhiXI-1 were modified by deleting the redundant fragment at the N terminus, and the deleted sequences are shown in dashed lines; RhoXI-1 was modified by replacing its C-terminal fragment with the C-terminal fragment from HasXI. Some point mutations were performed for AnaXI-2, NeoXI-2, RhoXI-1, CalXI-1, and BeaXI-1, and mutant amino acids are marked in green. (**B**) The xylose utilization and cell growth in YPX40 medium of *S. cerevisiae* strains expressing modified XIs (except RhiXI-1*) for 96 hours and the strain expressing RhiXI-1* for 120 hours. ATCC26603 and 26603* expressing the plasmid TDH3_G418_pESC-URA were used as the negative controls. PirXI and HasXI were used as the positive controls. (**C**) The xylose utilization and cell growth of *S. cerevisiae* expressing ancestral XIs in YPX40 medium for 96 hours. The data shown in (B) and (C) are presented as the mean value ± SDs of two or three biological replicates.

With the above understanding of the active and inactive XIs based on the amino acid sequences and simulated structures, inactive XIs were modified (Fig. 4), aiming to activate these potential XIs. According to sequence alignments and simulated structure analysis, AnaXI lacks a 22–amino acid fragment in its N terminus. Therefore, we assembled the N-terminal sequence of PirXI onto the N terminus of AnaXI, obtaining AnaXI-1. In addition, the histidine and alanine at the 60th and 325th positions were further mutated to glutamine and serine, respectively, on AnaXI-1 to be consistent with the aligned amino acids in active XIs, obtaining AnaXI-2. For NeoXI, there is a redundant sequence in its N terminus. Thus, we deleted the redundant N-terminal sequence of NeoXI, obtaining NeoXI-1. Then, glutamine, histidine, and alanine at 93rd, 346th, and 436th positions were further mutated to lysine, threonine, and glycine, respectively, to obtain NeoXI-2. For RhiXI, the N-terminal sequence of HasXI was assembled onto its N terminus, and the redundant sequence NTSIPFD was deleted, obtaining RhiXI-1. For RhoXI, the C-terminal fragment has multiple amino acids that differ from those in active XIs; thus, its C-terminal fragment was replaced by that of HasXI, resulting in RhoXI-1. For BeaXI and CalXI, multiple amino acid sites were modified according to conserved amino acids in active XIs, obtaining BeaXI-1 and CalXI-1, respectively (Fig. 4A).

As illustrated in Fig. 4B, among the eight modified XIs, RhoXI-1, BeaXI-1, and CalXI-1, which were mainly modified by a small number of amino acid substitutions, did not confer xylose utilization capability on *S. cerevisiae*. In contrast, NeoXI-1, NeoXI-2, AnaXI-1, AnaXI-2, and RhiXI-1, which were modified by missed fragment assembly or redundant fragment deletion, endowed *S. cerevisiae* with the ability to use xylose. In detail, NeoXI-1, NeoXI-2, AnaXI-1, and AnaXI-2 enabled xylose consumption of 18.61, 17.26, 11.55, and 10.63 g/liter within 96 hours, respectively. RhiXI-1 enabled xylose consumption of 7.32 g/liter within 120 hours. Although Aeling and colleagues (*41*) improved the activity of an XI from *Ruminococcus flavefaciens* by replacing the first 10 amino acids with the corresponding amino acids from PirXI, this is the first time that inactive XIs were turned into active XIs through sequence modification, which also suggested the necessity of the N-terminal fragment as a prerequisite. To further prove the significance of the N-terminal structure, the N-terminal fragments of active XIs (PirXI, HasXI, BajXI, AceXI, and StrXI) were truncated or extended (fig. S3). Results suggested that the activities of those XIs disappeared in *S. cerevisiae*.

**Fig. 5.**
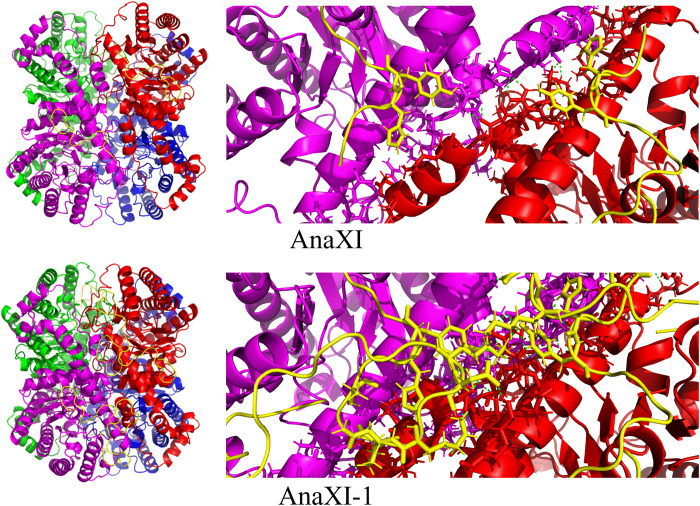
Predicted tetrameric structures of AnaXI and AnaXI-1 and the diagonal dimers generated by subunit B and subunit C. Subunits A, B, C, and D and the N-terminal extension of each subunit are colored blue, red, magenta, green, and yellow, respectively. For diagonal dimer, the interface residues between subunit B and subunit C are displayed in stick formats. The polar connections between subunit B and subunit C are displayed in green dashes. For diagonal dimers (formed by subunits A and D, and formed by subunits B and C), the appropriate N-terminal extension and linker subunit have multiple interface residues, creating enough intermolecular forces to maintain the structural stability.

As noted, XIs normally function in a dimer or a tetramer form (*42*, *43*), whose formation depends on the interactions between the cores and the N- and C-terminal tails of subunits. For diagonal dimers (formed by subunits A and D, and formed by subunits B and C), the appropriate N-terminal extension and linker subunit have multiple interface residues, creating enough intermolecular forces to maintain the structural stability (Fig. 5). Inadequate or redundant N-terminal extension may not be able to support proper dimer or tetramer formation. Although there are some inconsistent amino acid sites previously considered essential to the activity of XIs in NeoXI-1 and AnaXI-1, the presence of these divergent residues did not deactivate these two XIs, and the modification of these sites did not improve their activities (Fig. 4B). Similarly, amino acid site modification of BeaXI, RhoXI, and CalXI did not endow the relevant mutants with XI activity, even when 22 amino acid sites in BeaXI were adjusted to align with active XIs. With the results obtained here and in previous studies, it is suggested that mutations at individual residues are sometimes beneficial to boost the activity of naturally active XIs, whereas the completeness of N-terminal and C-terminal fragments is no doubt critical to maintaining basic XI activity.

### Reconstructing ancestral XIs for active expression in *S. cerevisiae*

Researchers have tested numerous XIs for active expression in *S. cerevisiae*, but only a small proportion of them exhibited activities, most of which are from *Firmicutes* and *Bacteroidetes* and are located in branches IV and IX of the evolutionary tree (Fig. 2A). It is possible that the ancestors of XIs from *Firmicutes* and *Bacteroidetes* were capable of functioning on xylose in *S. cerevisiae*, and as time went on, many mutations were generated during gene transfer and amplification, which could elevate, reduce, or lose the enzyme activity. To demonstrate the above hypothesis, we computationally constructed the ancestral sequences of XIs in branches IV and IX (table S5 and fig. S4). The obtained four ancestral sequences were distinct from known XI sequences in the public database, with the highest identities to existing sequences of only 82.15 to 90.14% (table S6). Excitingly, the expression of all four artificial XIs in *S. cerevisiae* enabled xylose consumption. In particular, the xylose utilization rates of the AncXI-1–, AncXI-2–, and AncXI-3–expressing strains were comparable to those of the HasXI- and PirXI-expressing strain (Fig. 4, B and C). The above results suggested that ancestral XIs have high potential for enabling *S. cerevisiae* to use xylose and that the activity lost during long-term evolution can be recovered through rational reconstruction. In addition to the regular *S. cerevisiae* culture conditions mentioned in this study, previous studies suggested that ancestral enzymes commonly exhibit high temperature stability, high pH tolerance, and high compatibility with multiple hosts (*44*–*46*). This means that these artificial ancestral XIs may have high potential in numerous scenarios in addition to the lignocellulosic ethanol process. The obtained ancestral XIs also provide references for finding other scarce and valuable industrial enzymes with different physicochemical properties and various types of products. With the aforementioned information, both suitable modifications and ancestral sequence reconstruction enabled the development of active XIs from several inactive XIs. As a vast amount of omics data become available, more genes originating from nonculturable or unconventional microorganisms are becoming easy to obtain, providing valuable resources for XI mining.

In summary, we used three methods to obtain active XIs for *S. cerevisiae*: (i) We screened active XIs based on the constructed phylogenetic tree. As a result, 4 of 15 tested XIs showed activity in *S. cerevisiae*. (ii) We engineered the inactive XIs on the basis of the information obtained from the sequence analysis and predicated protein structures. As a result, five of eight modified XIs were conferred activity in *S. cerevisiae*. (iii) We artificially created four XIs through computer reconstruction according to the phylogenetic footprinting of XIs. As a result, all the four XIs exhibited activity in *S. cerevisiae*. We think that the first method is the foundation of this study because the tested 15 XIs (both the active and inactive ones) provided valuable information for us to find key factors for XIs active in *S. cerevisiae*. According to the sequence information and predicated protein structures of reported XIs and XIs tested in this study, targeted protein engineering and sequence reconstruction were able to be performed. In particular, the success rates to find active XIs in *S. cerevisiae* were significantly improved with these two targeted methods. In just recent years, several advanced de novo protein design methods have been invented, such as Rosetta design (*47*) and SCUBA (side chain–unknown backbone arrangement)–driven structure design (*48*). Here, ancestral XI sequences were reconstructed by referring to the phylogenetic relationship between homologs and applying a statistical model of amino acid substitution to calculate sequences at internal nodes of the phylogenetic tree. We think that the ancestral sequence reconstruction method can be considered a primary method for de novo XI design. It is promising that some distinctive XIs may be de novo designed with the de novo protein design methods.

### The influence of *S. cerevisiae* hosts on XI expression

Just like the fact that there are immense XIs, there are also various *S. cerevisiae* in nature. The influence of *S. cerevisiae* hosts on XI expression was also investigated in this study. Eight XIs (including the classical XI from *Piromyces* sp., four XIs directly mined from the NCBI database, and three XIs obtained by protein engineering) were expressed in four *S. cerevisiae* strains (ATCC26603, a wild diploid yeast; CRD3, an ATCC26603 derivative with overexpression of the nonoxidative pentose phosphate pathway and some other modifications beneficial to xylose utilization; ATCC204508, a commonly used haploid yeast in laboratory; 16-4, a wild diploid yeast isolated by our laboratory, which exhibits high tolerance to many lignocellulosic inhibitors). To limit variation in the copy number of XI gene between strains, the same replicating plasmid pESC-URA was used for XI expression.

As presented in fig. S5, *S. cerevisiae* hosts indeed significantly affected the xylose utilization performance, showing that the expression of the same XI contributed to different fermentation results in different hosts. In particular, expression of PirXI, HasXI, BajXI, AceXI, and AnaXI-2 in CRD3 contributed to higher xylose consumption, higher cell growth, and higher ethanol production within 72 hours, compared to the expression of those genes in ATCC26603 strain, which suggested that overexpression of the nonoxidative pentose phosphate pathway and some other mentioned gene modifications enhanced xylose utilization. It was predicted that these five XIs have high catalytic ability, and other pathways are the rate-limiting steps for xylose assimilation. In contrast, the expression of StrXI, AncXI-1, or AncXI-3 in ATCC26603 and CRD3 contributed to similar fermentation results. It was probably because of the fact that the catalytic ability of these three XIs is low and is the rate-limiting steps for xylose assimilation. Moreover, the differences of intracellular environmental and posttranslational modification, which enable different XI activities, may be another reason for varied xylose utilization performances of different hosts. Nonetheless, regardless of the variation fermentation results, all selected XIs exhibited activity in all of the four hosts, which indicated that the active expression of XIs is decided by XIs themselves instead of *S. cerevisiae* hosts.

### Improving xylose utilization through ALE

With the mined active XIs, we attempted to construct efficient xylose-utilizing *S. cerevisiae* for lignocellulosic biorefinery. To avoid the need to use antibiotics for maintaining replicating plasmids, the codon-optimized nucleotides for the four mined XIs were integrated into delta sites [delta sites are repeated Ty retrotransposon delta sites, which enable multicopy integration of XI genes and automatic increase in the copy number of XI genes during cultivation (*49*)] of *S. cerevisiae* CRD3 chromosomes, obtaining strains CRD4HS, CRD4BJ, CRD4AE, and CRD4SC. These recombinant strains completely consumed xylose (40 g/liter) within 72, 84, 108, and 144 hours, respectively (fig. S6, A to D). To mimic the composition of lignocellulosic hydrolysate, these four strains were further cultured with glucose (80 g/liter) and xylose (40 g/liter). Results showed that all these four strains completely consumed glucose within 12 hours, while xylose utilizations were severely impaired, with only xylose at 10.69, 11.90, 8.80, and 9.37 g/liter consumed by the CRD4HS, CRD4BJ, CRD4AE, and CRD4SC strains, respectively, within 120 hours (fig. S6, E to H). The inhibition of glucose on xylose utilization due to carbon catabolite repression has been widely documented. Furthermore, the phenomenon that xylose utilization was still severely impaired after glucose depletion indicated by our results, was also observed by some other researchers, and was termed as “the post-glucose effect on xylose metabolism.” Transcriptome analysis suggested that glucose-sensing systems became lethargic after glucose depletion, which also contributed to weak xylose utilization (*50*, *51*). Further studies demonstrated that deletion of the transcription factor *THI2* and adenosine 3′,5′-monophosphate phosphodiesterase genes of *PDE1* and *PDE2*, overexpression of the ribosome-related genes of *RPL9A*, *RPL7B*, and *RPL7A*, and cell cycle–related gene *NRM1* were all beneficial to alleviate the post-glucose effect on xylose metabolism (*52*).

**Fig. 6.**
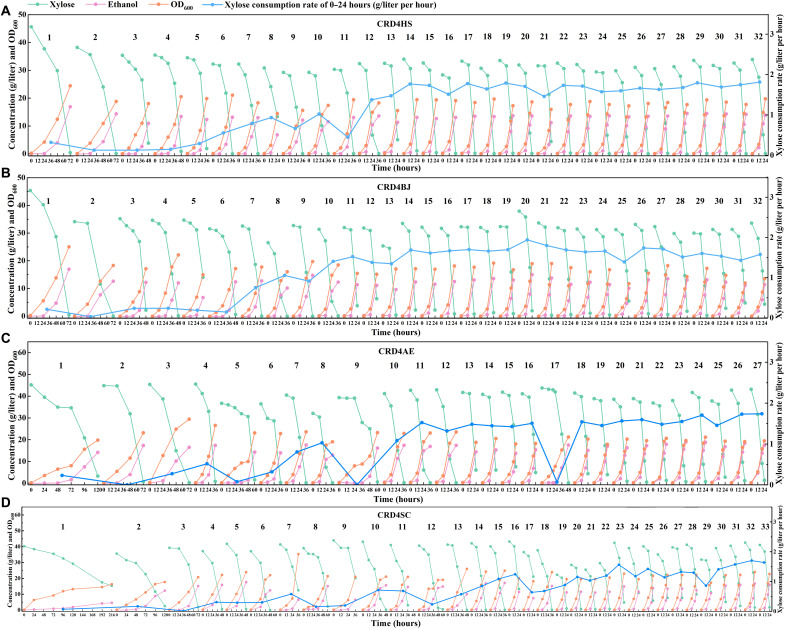
Improving xylose utilization through ALE. Strains were cultured in YPX40 medium at 30°C and 150 rpm under anaerobic conditions with an initial OD_600_ value of 0.2. When the OD_600_ value of the culture broth increased to approximately 20, the yeast cells were transferred to another fresh YPX40 medium. The initial strains used for ALE were CRD4HS (**A**), CRD4BJ (**B**), CRD4AE (**C**), and CRD4SC (**D**). The xylose consumption rate was calculated on the basis of xylose consumed from 0 to 24 hours. It is noteworthy that the delay period of CRD5AE at rounds 9 and 17 during ALE was more than 24 hours, so the xylose consumption rates of 0 to 24 hours were near 0.

To improve the strains’ capability for xylose utilization, ALE was carried out through sequential batch cultivation with xylose as the main carbon source. As presented in Fig. 6, ALEs were conducted for 41 to 58 days and 27 to 33 transfers, corresponding to 180 to 222 generations. In detail, the CRD4HS, CRD4BJ, CRD4AE, and CRD4SC strains took 72, 72, 120, and >216 hours, respectively, to consume up xylose (40 g/liter) during the first round of cultivation, with xylose utilization rates of 0.33, 0.21, 0.23, 0.07 g/liter per hour in the first 24 hours. As ALE progressed, the time required to consume xylose (40 g/liter) was gradually reduced to and maintained at approximately 24 hours, with xylose utilization rates of 1.41 to 1.82 g/liter per hour for these four groups. Apparently, compared with the CRD4AE and CRD4SC strains, the CRD4HS and CRD4BJ strains acquired faster xylose utilization capability during ALE. These phenomena imply that the activities of HasXI and BajXI are higher than those of AceXI and StrXI when they are expressed in *S. cerevisiae* or that CRD4HS and CRD4BJ acquired better mutations to support xylose utilization during ALE.

During ALE, cells are confronted with phenotypic heterogeneity and trade-off situations (*53*, *54*). To screen for strains with high and stable utilization capabilities for both glucose and xylose, the evolved populations were cultivated in YPD20 liquid medium [glucose (20 g/liter) added as the main carbon source] for five consecutive transfers and then plated on YPX40 medium [xylose (40 g/liter) supplemented as the main carbon source]. Larger colonies were selected and inoculated into YPX40 liquid medium to test their xylose utilization capability. As illustrated in fig. S7, most colonies grew slowly in YPX40 liquid medium, with only a few colonies exhibiting excellent xylose utilization performance, which implies that only a few cells acquired sufficient positive mutations for xylose utilization during the ALE processes. Evolved strains with a high growth rate in xylose culture were chosen and named CRD5HS, CRD5BJ, CRD5AE, and CRD5SC. The xylose utilization, cell growth, and ethanol production of CRD5HS, CRD5BJ, CRD5AE, and CRD5SC were similar to those in the stage with the highest xylose utilization rate and those in the last stage. In addition, even after a protracted period without xylose culture, these four evolved strains still kept steady xylose fermentation capability (fig. S8).

**Fig. 7.**
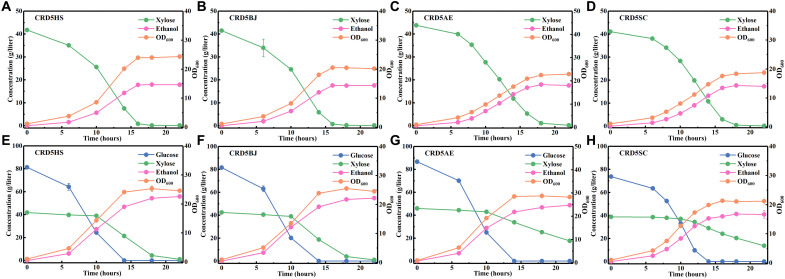
Fermentation performances of evolved strains with xylose as the main carbon source or with glucose and xylose as the main cocarbon source. The evolved strains used here were CRD5HS (**A** and **E**), CRD5BJ (**B** and **F**), CRD5AE (**C** and **G**), and CRD5SC (**D** and **H**). The medium used for (A) to (D) was YPX40 medium [yeast extract (10 g/liter), tryptone (20 g/liter), and xylose (40 g/liter)]; the medium used for (E) to (H) was YPD80X40 medium [yeast extract (10 g/liter), tryptone (20 g/liter), glucose (80 g/liter), and xylose (40 g/liter)]. The data shown in (A) to (H) are presented as the mean value ± SD of two biological replicates.

When xylose (40 g/liter) was used as the main carbon source, CRD5HS and CRD5BJ consumed up xylose (40 g/liter) within 16 hours with specific xylose consumption rates of 0.851 and 0.832 g/g dry cell weight (DCW) per hour, respectively (Fig. 7, A and B). In contrast, the time for CRD5AE and CRD5SC was 18 hours, and the specific xylose consumption rates were 0.698 and 0.808 g/g DCW per hour (Fig. 7, C and D). The ethanol yields of these four strains were all about consumed xylose (0.43 g/g), among which CRD5HS achieved the highest of consumed xylose (0.437 g/g; table S7). When glucose (80 g/liter) and xylose (40 g/liter) were supplied as cocarbon sources to mimic lignocellulosic hydrolysate, the evolved strains, especially CRD5HS and CRD5BJ, showed satisfactory performance with all glucose and xylose consumed within 24 hours (Fig. 7, E to H). In light of the fermentation performances of their parent strains, the xylose fermentation capacities of the evolved strains were significantly improved. Moreover, these four yeast strains also showed rapid xylose consumption under aerobic conditions, demonstrating high efficiencies of the mined isomerases during aerobic cultivation and showing great potential in applying these XIs to produce biochemicals that require aerobic cultivation (fig. S9). We also constructed the xylose-utilizing strain, *S. cerevisiae* CRD51, from the CRD3 strain with the same construction and ALE methods, except that PirXI was used for xylose utilization. It is worth noting that CRD51 performances were as good as those four strains except that CRD5HS received slightly higher xylose utilization rate (fig. S11), validating the effectiveness of the mined XIs from another side.

### Genome sequencing unveiled mutations occurring in evolution

**Fig. 8.**
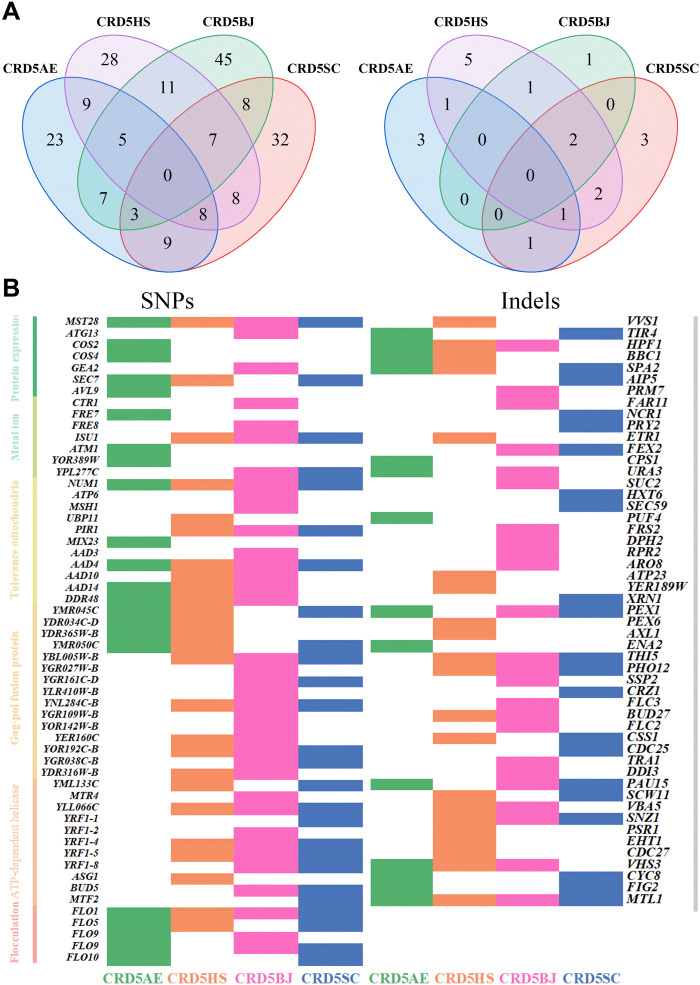
Genome resequencing unveiled mutations occurring in ALE. (**A**) Venn diagram of SNPs and Indels occurring in evolved strains of CRD5AE, CRD5HS, CRD5BJ, and CRD5SC. (**B**) Mutant genes occurring in four evolved strains. The mutant genes on the left can be classified into seven categories (protein expression, metal ion, mitochondria, stress tolerance, gag-pol fusion protein, Y′ element adenosine 5′-triphosphate (ATP)–dependent helicase, and flocculation), while the genes on the right are not classified.

During ALE, the gene copy number of XI in evolved strains increased, with CRD5HS having twice as much copies of XI gene as CRD51 carrying PirXI (fig. S10), indicating that the number of copies of XI gene remains a key parameter for improved performances. In addition, to elucidate other genetic basis underlying the improved xylose utilization of the evolved strains, genome resequencing was conducted on the four evolved yeasts, with the parental CRD3 strain as the reference strain. In general, there were no base mutations for introduced XIs and other introduced genes related to the hexose transporter and pentose phosphate pathway except a single point mutation on hexose transporter protein gene *HXT6* (A556T) of CRD5SC (table S9). A total of 203 nonsynonymous single-nucleotide polymorphisms (SNPs) in 98 genes and 20 Indels in 17 genes were found in the four evolved yeasts. Among them, four genes with SNPs, including *AAD4*, *MST28*, *NUM1*, and *FLO1*, were found in all four evolved strains, while there were no identical SNPs (Fig. 8).

According to the genome sequencing results, genes related to organelles of the endoplasmic reticulum, Golgi, anti-Golgi network, endosomes, and vacuoles, which are commonly involved in protein folding, subunit assembly glycosylation, and transport (*55*), are the most abundant mutants during ALE. In addition, many genes related to the biosynthesis of vesicles, which are responsible for transporting synthesized proteins to target organelles or the cytomembrane, are also mutated. These mutated genes may contribute to improved subunit assembly or improved XI expression. Some genes associated with metal ion transport or concentration maintenance were also mutated during ALE. Divalent metal ions are essential for XI activity (*40*), and thus, mutations influencing metal ion homeostasis are likely to affect XI activity. Aryl-alcohol dehydrogenases (AADs) are enzymes that catalyze oxidation-reduction reactions converting alcohols to corresponding aldehydes or the reverse reactions (*56*). DNA damage response (DDR) family members are commonly involved in DNA repair and environmental stress tolerance, and their gene expression is commonly up-regulated in response to environmental shock. Here, the mutation of *AAD* and *DDR48* during ALE may be beneficial to maintain the stability of the intracellular environment for xylose metabolism. Flocculation genes contain multiple repeats and are prone to mutation during replication processes. The variation in flocculent protein, gag pol fusion protein, and Y′ element adenosine 5′-triphosphate–dependent helicase (*57*) during ALE is more likely caused by natural diversity than by xylose utilization. There are many additional genes involved in the biosynthesis or functional execution of pheromones, the cell wall, sterols, ubiquitin, actin, and mannoprotein that are also mutated during ALE. They may not directly participate in xylose utilization but promote xylose metabolism in other ways.

Overall, most SNPs and Indels mutations occurred in genes with versatile physical functions and not in genes directly involved in the xylose metabolic pathways (xylose isomerization, xylulose phosphorylation, nonoxidative pentose phosphate pathway, glycolytic pathway, etc.). The effectiveness of these gene mutations in improving xylose utilization will be evaluated in future work, which may deepen our understanding of xylose metabolism and provide valuable targets for efficient xylose-utilizing strain construction.

### Applying developed xylose-utilizing *S. cerevisiae* in corn stover biorefinery

Although lignocellulosic biomass represents the largest renewable bioresource in the ecosphere, starch-derived glucose is still the most commonly used carbon source in the fermentation industry (*58*, *59*). One important reason is that many currently used fermentation strains cannot use xylose. With the mined active XIs and the newly developed efficient xylose-utilizing *S. cerevisiae* strains, we studied bioethanol production from corn stover, a typical agricultural lignocellulosic biomass. A pretreatment approach of densifying lignocellulosic biomass with alkaline/acidic chemicals followed by a regular steam Autoclave (DLCA) invented in our laboratory was used to pretreat corn stover with calcium hydroxide as the pretreatment reagent (*60*, *61*). Then, the pretreated biomass (containing 31.63 wt % glucan and 15.03 wt % xylan) was enzymatically hydrolyzed at a solid loading of 30 wt % for 72 hours, obtaining lignocellulosic hydrolysate containing glucose (116.31 g/liter) and xylose (42.90 g/liter). Thereafter, xylose-utilizing *S. cerevisiae* strains constructed and evolved in this study were inoculated into the hydrolysate for bioethanol fermentation. It should be noted that no solid-liquid separation, washing, or detoxification was applied during or after DLCA pretreatment. Therefore, the hydrolysate contained a small amount toxic compounds (although not as much as the hydrolysates using traditional pretreatment methods), as determined by our previous study (*60*, *61*).

**Fig. 9.**
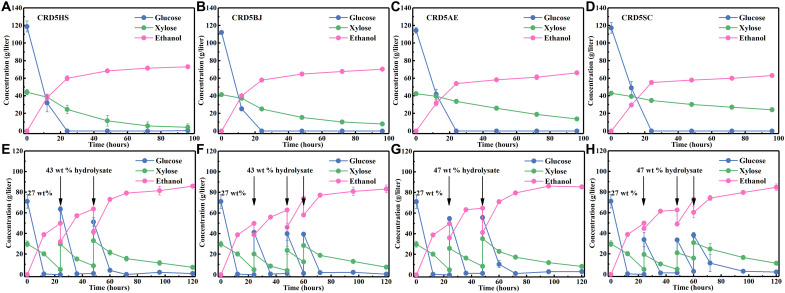
Batch and fed-batch fermentation with DLCA(ch) corn stover as feedstock. For batch fermentation, DLCA(ch) corn stover was hydrolyzed at 30 wt % for 72 hours, and then CRD5HS (**A**), CRD5BJ (**B**), CRD5AE (**C**), or CRD5SC (**D**) was inoculated for fermentation. For fed-batch fermentation, 27 wt % DLCA(ch) corn stover was hydrolyzed for 14 hours, and then CRD5HS was inoculated for fermentation. To reach a total solid loading of 35 wt %, isovolumetric 43 wt % DLCA(ch) corn stover hydrolyzed for 72 hours was equally added into the fermentation system at 24 and 48 hours (**E**) or at 24, 48, and 60 hours (**F**); to reach a total solid loading of 37 wt %, isovolumetric 47 wt % DLCA(ch) corn stover hydrolyzed for 72 hours was equally added into the fermentation system at 24 and 48 hours (**G**) or at 24, 48, and 60 hours (**H**). The data shown in (A) to (H) are presented as the mean value ± SD of two biological replicates.

As shown in Fig. 9 (A to D), all four strains completely consumed glucose (116.31 g/liter) in the hydrolysate within 24 hours, and thereafter, the cells rapidly consumed xylose. As a result, ethanol titers as high as 73.06, 70.38, 66.16, and 63.00 g/liter were produced by CRD5HS, CRD5BJ, CRD5AE, and CRD5SC, respectively, at 96 hours, with residual xylose at 4.29, 8.04, 13.70, and 24.19 g/liter remaining in the fermentation broth. Analogous to the fermentation results in YPDX medium, CRD5HS and CRD5BJ exhibited more powerful xylose utilization capacity than CRD5AE and CRD5SC. In the ethanol industry, a high ethanol titer is a prerequisite for improving productivity, facilitating distillation, and reducing both capital and operating costs (*1*, *62*). Here, we attempted to achieve higher ethanol titers by increasing biomass solid loading. It should be noted that in addition to raising the glucose and xylose concentrations, higher solid loadings also lead to higher inhibitor concentrations, higher viscosity of the system, and higher osmotic pressure, all of which hamper yeast viability and xylose utilization capacity, as xylose metabolism is sensitive to these factors (*63*, *64*). In addition, an increased glucose concentration also inhibits on xylose utilization. The undesirable effects caused by increased solid loading were reflected in the fermentation results, as expected, when the solid loading increased from 30 to 35 wt % (fig. S13), which led to much xylose remaining unused in the fermentation broth.

Fed-batch operations are beneficial to alleviate the high viscosity and high osmotic pressure caused by high solid loading (*1*). Therefore, fed-batch operations were performed for high loading fermentation at initial solid loadings of 27 wt %, with high solid loading hydrolysate of 43 wt % added during the fermentation process to achieve a total solid loading of 35%. The prehydrolysis time for a biomass solid loading of 27 wt % was shortened to 14 hours to reduce the initial concentrations of glucose, xylose, and inhibitors, allowing CRD5HS to grow rapidly and consume glucose and xylose efficiently. When 43 wt % hydrolysate was added to the fermentation system, the glucose and xylose were diluted to low concentrations and effectively consumed by CRD5HS. Last, two and three batch operations yielded ethanol (85.95 and 83.33 g/liter, respectively), with residual xylose (7.17 and 7.34 g/liter) (Fig. 9, E and F). The impact of biomass solid loading and feeding mode was also reflected in the ethanol yield. The yield of 30 wt % separate hydrolysis and cofermentation process, 35 wt % simultaneous saccharification and cofermentation (SSCF) process, and 35 wt % fed-batch process with CRD5HS as fermentation strain were 71, 62, and 70%, respectively (table S8). When the total fermentation solid loading was increased to 37 wt %, a very high solid loading, and the corresponding solid loading of supplementary hydrolysate was increased to 47 wt %, the hydrolysis performance deteriorated, resulting in viscous fermentation broth and decreased glucose and xylose release. The final ethanol titers were 85.47 and 84.63 g/liter (Fig. 9, G and H) after two and three batches of hydrolysate addition, respectively. Overall, ethanol (approximately 85 g/liter) was achieved in all four designed fed-batch fermentations with corn stover as the feedstock and the developed xylose-utilizing *S. cerevisiae* as the fermentation strain. These are the highest ethanol titers yet obtained from corn stover, although no solid-liquid separation, washing, or detoxification was applied during pretreatment, hydrolysis, and fermentation processes. Moreover, the mined XIs well functioned in *S. cerevisiae* under both anaerobic or aerobic conditions. Thus, there is tremendous potential for applying these XIs in lignocellulosic biorefinery, not only for bioethanol production but also for other biochemicals production.

### Applying developed xylose-utilizing *S. cerevisiae* in corn cob biorefinery

In addition to corn stover, corn cob is another massive agricultural lignocellulosic biomass. The mass ratio of corn cob and aboveground dry matter (grain plus other biomass) is approximately 8 to 9 wt %, with yields ranging from 1.42 to 1.53 dry t/ha (*65*). Compared with corn stover and other typical lignocellulosic biomass types, corn cob has a significantly higher xylan content. Thus, corn cob valorization requires high xylose conversion. Corn cob was pretreated by the DLCA method with sulfuric acid as the pretreatment reagent. The DLCA-pretreated corn cob (containing 24.59 wt % glucan and 24.77 wt % xylan) was hydrolyzed at 25 wt % solid loading, yielding glucose (84.55 g/liter) and xylose (74.14 g/liter). As shown in fig. S15 (A to D), ethanol of 72.57, 72.42, 60.90, and 57.73 g/liter was produced at 96 hours by CRD5HS, CRD5BJ, CRD5AE, and CRD5SC, respectively, with residual xylose of 4.89, 6.43, 24.46, and 38.05 g/liter remaining unused. The results showed that CRD5HS and CRD5BJ consumed xylose (approximately 70 g/liter), confirming their excellent xylose consumption capabilities. However, when the solid loading was increased to 30 wt %, the xylose utilization and ethanol yield became poor due to the increasing inhibitor, glucose, and xylose concentrations (fig. S14 and table S8). Therefore, fermentations performed simultaneously with saccharification (i.e., SSCF) at high solid loadings of 35, 37, and 40 wt % were conducted via fed-batch methods. Because of the easily hydrolyzed properties of DLCA(sa) corn cob, the supplementary feedstocks can be added directly during fermentation without prehydrolysis. As illustrated in fig. S15 (E and F), during the entire fermentation process at solid loadings of 35 and 37 wt %, the xylose concentration was maintained at low levels owing to the fed-batch operations. As a result, ethanol titers as high as 89.94 and 90.87 g/liter were produced from corn cob in 96 hours, respectively. These results indicated that fed-batch fermentation was favorable for xylose consumption and ethanol production at high solid loadings. When the solid loading was further increased to 40% (an extremely high solid loading), ethanol (84.36 and 94.76 g/liter) was produced with two fed-batch operations (fig. S15, G and H). The results suggest that fed-batch operations with small quantities added at a time can achieve better fermentation performances. Overall, for the strain constructed with the mined XI from *H. seregens*, ethanol (89.94, 90.87, and 94.76 g/liter) was produced from 35, 37, and 40 wt % DLCA-pretreated corn cob, respectively. These ethanol titers, to our knowledge, are the highest achieved on unwashed and undetoxified pretreated lignocellulosic biomass and are close to the ethanol titers produced with corn kernel as the feedstock. It was also likely the first time a yeast strain successfully fermented both glucose and xylose from an undetoxified lignocellulosic biomass at such high solid loadings.

## MATERIALS AND METHODS

### Microorganisms, plasmids, and primers

The microorganisms, plasmids, and primers used in this study are provided in tables S1 and S2. In particular, the diploid yeast *S. cerevisiae* ATCC26603 and CRD3 were used as a host for testing the activities of mined XIs, mutated XIs, and artificial ancestral XIs. Strain CRD3 is a derivative of strain ATCC26603 with modified genotypes for enhancing xylose metabolism, including overexpressing the xylose kinase gene *XKS1* and the pentose phosphate pathway genes *TKL1*, *RPE1*, *RKI1*, and *TAL1*, introducing a xylose specific transporter gene of *GAL2*^N376F^, and deleting aldose reductase gene *GRE3* and 4-nitrophenylphosphatase gene *PHO13*. The replicating plasmid pESC-URA provided by GenScript Corporation (www.genscript.com) was used for XI expression in *S. cerevisiae*. The pML104 plasmid–mediated CRISPR-Cas9 system was used to integrate XI genes into the *S. cerevisiae* chromosome (*66*).

### Media and materials

LB medium [yeast extract (5 g/liter), tryptone (10 g/liter), and NaCl (10 g/liter)] was used for the routine culture of *E. coli*; YPD20 medium [yeast extract (10 g/liter), tryptone (20 g/liter), and glucose (20 g/liter)] was used for the routine culture of *S. cerevisiae*; YPX40 medium [yeast extract (10 g/liter), tryptone (20 g/liter), and xylose (40 g/liter)] was used for the evolution of recombinant *S. cerevisiae*; and YPD80X40 medium [yeast extract (10 g/liter), tryptone (20 g/liter), glucose (80 g/liter), and xylose (40 g/liter)] and YPX40 medium were used to test the xylose utilization capability of the constructed or evolved *S. cerevisiae* strains.

The corn stover and corn cob used in this study were purchased from a farm in Lianyungang, Jiangsu, China. The corn stover contained 37.19 wt % glucan, 19.20 wt % xylan, and 19.45 wt % lignin, and the corn cob contained 30.1 wt % glucan, 29.4 wt % xylan, and 20.3 wt % lignin.

### Phylogenetic tree construction for XIs

With “XI” as the search word, approximately 250,000 results were obtained from the “Protein” module of NCBI (www.ncbi.nlm.nih.gov/protein/). According to the basic traits of previously reported XIs, the obtained sequences were preliminarily screened for lengths of 370 to 470 amino acids and then clustered using Cdhit with a threshold for dereplication at 73%, which resulted in 1042 putative XI sequences. Thereafter, sequences with conserved sites (metal binding residues of H102, D105, E233, K235, E269, H272, D297, D308, D310, and D340, as well as the active pocket surrounding residues of W50, F61, F146, W140, and W189; coordinates are based on PirXI amino acid sequences) were further retained. The obtained sequences and 16 reported XIs that are active in *S. cerevisiae* were subjected to phylogenetic tree construction using MEGA11 with the maximum likelihood method. The obtained phylogenetic tree was optimized using the online software Interactive Tree Of Life (https://itol.embl.de/).

### Expression of XIs in *S. cerevisiae* with a replicating plasmid

The nucleotide sequences encoding 15 selected XIs were codon optimized by GenSmart Codon Optimization (www.genscript.com/gensmart-free-gene-codon-optimization.html) according to the codon preferences of *S. cerevisiae* and synthesized by GenScript Corporation (Nanjing, China). The nucleotide sequences encoding four ancestral sequences of XIs and BeaXI-1 were codon-optimized and synthesized by Tsingke Biotechnology Co. Ltd. (Nanjing, China). It should be pointed out that potential differences may be caused by different codon optimization tools. The replicating plasmid pESC-URA with the G418 resistance gene and the strong *TDH3* promoter inserted was used for XI expression in *S. cerevisiae*. The synthesized or mutated XI gene fragments were individually inserted into the site between the *TDH3* promoter and the *CYC1* terminator. The verified XI-containing plasmids were transformed into *S. cerevisiae* ATCC26603 or CRD3 by the LiAc/SS carrier DNA/poly(ethylene glycol) (PEG) method. Recombinant yeasts were screened by G418 (400 μg/ml) with polymerase chain reaction (PCR) verification for target XI genes. After that, verified XI-containing yeasts were inoculated into YPX40 medium [supplemented with G418 (400 μg/ml) with an initial optical density at 600 nm (OD_600_) of 1.0 for cultivation at 30°C and 150 rpm under anaerobic conditions]. Samples were withdrawn at intervals to determine cell growth, residual xylose, and ethanol production.

### Structure predication for XIs

The structure prediction for XIs was performed through the AlphaFold v2.0 program, whose prediction accuracy is comparable with experimental results in many cases (*67*). The source code of the AlphaFold v2.0 program is publicly available at https://github.com/deepmind/alphafold. The predicted structures and key functional sites were visualized by PyMOL. The interface residues and polar connections between different subunits of XIs were analyzed and visualized using PyMOL.

### Calculation of sequence identities and RMSDs

The 31–amino acid sequences encoding XIs (15 XIs tested in this study and 16 reported XIs active in *S. cerevisiae*) were aligned by using the Align X program in Vector NTI. The identities between any two amino acid sequences were obtained from the identity table, and relevant heatmaps were prepared by Origin 2019 (OriginLab Corp., USA). The comparison of predicted XI structures was performed by pairwise structure alignment using jFATCAT in the Research Collaboratory for Structural Bioinformatics PDB (www.rcsb.org/alignment) to obtain RMSD data. The corresponding heatmap was prepared with Origin 2019 according to the obtained RMSD data.

### Construction of ancestral sequences of XI

The selected nodes for ancestral sequence construction are shown in the developed XI phylogenetic tree (Fig. 2A). The ancestral sequences of XI were reconstructed by PAMLX software according to the surviving amino acid sites under the selected nodes.

### Chromosomal integration of XI genes

The expression cassettes of AceXI, BajXI, HasXI, and StrXI were individually integrated into the delta sites of the *S. cerevisiae* CRD3 chromosome through CRISPR-Cas9–mediated gene insertion. In detail, the G418 resistance gene was cloned into the Hind III/Eco RI site of plasmid pML104, obtaining recombinant plasmid pML-G418. The 20-nucleotide oligomer guide sequence targeting the *S. cerevisiae* CRD3 delta locus (referenced from http://crispr.dbcls.jp/) was introduced into the single guide RNA expression cassette in plasmid pML-G418, generating plasmid pML-delta. Then, the upstream and downstream regions of the delta locus were amplified from the *S. cerevisiae* CRD3 genome. Thereafter, upstream fragment of the delta locus, *TDH3* promoter, target XI gene fragment, *CYC1* terminator, and downstream fragment of the delta locus were assembled through overlapping PCR. The obtained DNA fragments and plasmids pML-delta were cotransformed into *S. cerevisiae* CRD3 through the LiAc/SS carrier DNA/PEG method. The integration of the XI expression cassette was verified by PCR, and then the plasmid pML-delta was cured by culturing without selection for three transfers, obtaining recombinants with the XI expression cassette integrated into the delta locus of the *S. cerevisiae* CRD3 chromosome, named *S. cerevisiae* CRD4AE, CRD4BJ, CRD4HS, and CRD4SC.

### ALE to improve xylose utilization

Single colonies of *S. cerevisiae* CRD4HS, CRD4BJ, CRD4AE, and CRD4SC were first cultivated in YPD20 medium until the OD_600_ value of the culture broth reached approximately 10. Then, the cultured yeast cells were inoculated into 50 ml of YPX40 medium in Erlenmeyer flat-bottom flasks with an initial OD_600_ of 0.2 to initiate ALE. ALE was performed at 30°C and 150 rpm under anaerobic conditions, and cell growth was monitored by measuring the OD_600_ every 12 or 24 hours. When the OD_600_ value of the culture broth reached approximately 20 (indicating that xylose was almost completely consumed), 0.5 ml of culture broth was manually transferred into another fresh YPX40 medium. When the time required for consuming xylose (40 g/liter) stopped decreasing, ALE was terminated. In particular, 1 ml of culture broth from every transfer was stored in 20% glycerol stocks at −80°C. The number of generations during ALE was calculated using the formula: number of generations = log_2_(OD_final_/OD_initial_) (*68*). The xylose utilization rate was calculated using the formula: xylose utilization rate = (xylose concentration_0h_ − xylose concentration_24h_)/24.

Two populations obtained from the endpoint stage of ALE and from the ALE stage with the fastest xylose utilization rate were applied to screen mutants with high xylose utilization capability. Broth samples withdrawn from the above two stages of the ALE process were first cultured in YPD20 medium for five transfers. Thereafter, the obtained broth was plated on YPX40 agar medium and cultivated at 30°C for 48 hours. Large colonies were picked to determine their xylose utilization capabilities. For each evolved lineage, the selected strain with the fastest growth rate was identified. These strains were named CRD5HS, CRD5BJ, CRD5AE, and CRD5SC and saved at −80°C for further experiments and analyses.

### Enzyme activity assay for XIs

The activity assay for XIs in *S. cerevisiae* was performed as described in previous studies (*69*). These XI-containing yeasts were first cultured in YPD20 medium at 150 rpm and 30°C until the OD_600_ value of their culture broth reached 3.0. Then, yeast cells were harvested by centrifugation at 4°C and 8000 rpm and washed twice with sterile water. The washed cells were resuspended in 100 mM tris-HCl (pH 7.5) buffer containing protease inhibitors of phenylmethylsulfonyl fluoride and sodium fluoride, as well as acid-washed glass beads [wet yeast (2 g/g)]. Yeast cells were disrupted by a bead beater in an ice bath. The disrupted cell solutions were centrifuged at 4°C and 12,000 rpm for 10 min, and the supernatant was collected as the crude enzyme solution for the XI activity assay. The protein concentration of the crude enzyme solution was determined by the Bradford Coomassie brilliant blue method. The XI activity assay system was prepared as follows: 66 mM xylose, 10 mM MgCl_2_, 100 mM tris-HCl buffer (pH 7.5), and 1 ml of crude enzyme solution, with a total volume of 3 ml. The reaction was conducted at 30°C for 30 min and terminated by boiling in a water bath for 10 min. Xylose consumption and xylulose production were measured using high-performance liquid chromatography (HPLC) equipped with an HPX-87H column. As presented in fig. S16, xylose, xylulose, and xylitol can be well separated by the HPX-87H column. One unit of XI activity was defined as the enzyme amount required to form 1 μM xylulose/min under the assay conditions. What needs to be pointed out is that the generated xylulose in this system can be converted by the inherent xylulose kinase in crude enzyme solution; thus, the obtained XI activities are lower than the real values.

### Genome resequencing and mutation analysis

The evolved *S. cerevisiae* strains CRD5HS, CRD5BJ, CRD5AE, and CRD5SC were cultured overnight in YPX40 medium, and the parent strain *S. cerevisiae* CRD3 was cultured overnight in YPD20 medium. Then, cultured cells were collected by centrifugation, and the obtained cell pellets were frozen in liquid nitrogen. Genome resequencing and analysis were performed by Origingene company (Shanghai, China) using PacBio Sequel II sequencing combined with Illumina NovaSeq 6000 sequencing. Genome assembly was performed by Hifiasm software (v0.16.0) (https://github.com/chhylp123/hifiasm). Calibration was performed by comparing CRD3 tri-generation data to the assembled results using Racon software (v1.4.20) (https://github.com/lbcb-sci/racon). The calibrated genome sequences were lastly compared with CRD3 resequenced data using pilon (v1.22) (https://github.com/broadinstitute/pilon) to obtain the final calibrated CRD3 sequences. The CRD5HS, CRD5BJ, CRD5AE, and CRD5SC resequencing results were compared with the obtained CRD3 reference genome using the BWA software (http://bio-bwa.sourceforge.net/) to identify genetic variants (SNPs and InDels). The mutant loci were annotated and then filtered using ANNOVAR software, and the mutated genes were annotated with the Nr, genes, string, and Gene Ontology databases. Relevant genome data have been added to NCBI with the accession number of PRJNA901870.

### Testing the xylose utilization capability of the strains in semisynthetic medium

To test the xylose utilization capability of the constructed yeasts, they were first fermented with semisynthetic medium alone. In detail, yeast cells cultured overnight in YPX40 medium were inoculated into YPX40 or YPD80X40 medium with an initial OD_600_ value of 1.0. Then, fermentation was conducted in 250-ml flasks with 50-ml liquid volume at 30°C and 150 rpm under anaerobic conditions. To maintain anaerobic conditions, flasks were capped with rubber stoppers. Needles were used to pierce rubbers to release the carbon dioxide generated during fermentation. Samples were withdrawn during fermentation to determine cell growth, glucose and xylose consumption, and ethanol production. Product yields were calculated using the formula: product yield = produced product/(consumed xylose + consumed glucose)/0.51. The specific xylose consumption rate, specific ethanol production rate, and maximum growth rates (μ_max_) were calculated as previously described (*70*). The coefficient between DCW and OD_600_ was 0.34.

### Pretreatment of corn stover and corn cob

The glucan and xylan contents of corn stover and corn cob were measured using Standard Biomass Analytical Methods provided by the National Renewable Energy Laboratory. Corn stover was pretreated by the DLCA(ch) method invented by our group (*60*, *61*). First, calcium hydroxide suspension was evenly sprayed onto corn stover, with calcium hydroxide and water dosages of 0.15 and corn stover (0.5 g/g), respectively. Then, the corn stover was pelleted by a flat die pellet machine, obtaining DLC(ch)-pretreated corn stover, which was further thermally treated at 121°C for 60 min to accelerate the pretreatment process, obtaining DLCA(ch) corn stover. DLCA(ch) corn stover was neutralized with sulfuric acid and air-dried in a fume hood for later hydrolysis and fermentation.

Corn cob was pretreated by the DLCA(sa) method. Similar to the procedures of the abovementioned DLCA(ch) method, sulfuric acid solution was first evenly sprayed on the corn cob, with sulfuric acid and water dosages of 0.075 and corn cob (0.5 g/g), respectively, and then the corn cob was pelleted by a flat die pellet machine, obtaining DLC(sa)-pretreated corn cob, which was further thermally treated at 121°C for 30 min to accelerate the pretreatment process, obtaining DLCA(sa) corn cob. DLCA(sa) corn cob was neutralized with calcium hydroxide and air-dried in a fume hood for later hydrolysis and fermentation. In particular, the pretreated biomass was used without washing or detoxification.

### Batch fermentation with pretreated corn stover and corn cob as feedstocks

To test the fermentation performances of *S. cerevisiae* strains on real lignocellulosic biomass, DLCA(ch) corn stover and DLCA(sa) corn cob were used. First, DLCA(ch) corn stover or DLCA(sa) corn cob was enzymatically hydrolyzed in 50-ml flasks with a 30-ml liquid volume at pH 4.8, 50°C and 250 rpm for 72 hours using Cellic Ctec2 (87 mg protein/ml) with a dosage of 20 mg protein/g glucan. The solid loadings of DLCA(ch) corn stover and DLCA(sa) corn cob were set at 25, 30, or 35 wt %, with half biomass and cellulase added initially and the remaining half biomass and cellulase added at 4 hours. *S. cerevisiae* CRD5HS, CRD5BJ, CRD5AE, and CRD5SC cultured in YPX40 served as fermentation seeds and were inoculated into hydrolysate [supplemented with yeast extract (5 g/liter) and tryptone (10 g/liter)] with an initial OD_600_ value of 2.0 for fermentation. Fermentation processes were conducted at pH 5.5, 30°C, and 150 rpm under anaerobic conditions.

### Fed-batch fermentation with pretreated corn stover and corn cob as feedstocks

To address the problems caused by adding massive amounts of biomass at one time, DLCA(ch)-pretreated corn stover and DLCA(sa)-pretreated corn cob were used for ethanol production at high solid loadings of 35, 37, or 40 wt % by fed-batch operations. All fed-batch fermentation with pretreated corn stover and corn cob as feedstocks were performed in 50-ml flasks with a 30-ml final liquid volume.

For DLCA(ch)-pretreated corn stover, 27 wt % feedstock was hydrolyzed for 14 hours, and then CRD5HS with an OD_600_ of 2.0 was inoculated for fermentation. Thereafter, 43 wt % DLCA(ch) corn stover was hydrolyzed for 72 hours and equally added to the fermentation system at 24 and 48 hours or at 24, 48, and 60 hours to reach a total solid loading of 35 wt %. In another scenario, 47 wt % DLCA(ch) corn stover was hydrolyzed for 72 hours and then equally added into the fermentation system at 24 and 48 hours or at 24, 48, and 60 hours to reach a total solid loading of 37 wt %.

For 35 wt % solid loading of DLCA(sa) corn cob, 33.4% feedstock and cellulase were added at the initial time for prehydrolysis. Thereafter, the remaining corn cob and cellulase were added in equal amounts at 24 and 48 hours of the fermentation process. For 37 wt % solid loading scenario, 31.5% corn cob and cellulase were added for prehydrolysis. Thereafter, the remaining 31.5, 31.5, and 5.5% corn cob and cellulase were added at 12, 24, and 48 hours of the fermentation process. Two fed-batch operation modes were applied for the 40 wt % solid loading scenario. Mode 1: Fifty percent corn cob and cellulase were added initially for prehydrolysis. The remaining corn cob and cellulase were added into the fermentation system in equal parts at 24 and 48 hours; mode 2: Twenty-five percent corn cob and cellulase were added initially for prehydrolysis. The remaining corn cob and cellulase were equally added into the fermentation system at 12, 24, and 48 hours. The prehydrolysis processes were carried out at pH 5.5, 30°C, and 250 rpm for 12 hours. The CRD5HS strain formed and evolved in this study was inoculated at 0 hours of fermentation with an initial OD_600_ value of 20.

### Quantitative analysis

The concentrations of glucose, xylose, xylulose, xylitol, and ethanol of hydrolysis and fermentation samples were determined by an HPLC system (Agilent, 1260 Infinity II, USA) equipped with an Aminex HPX-87H column (300 mm by 7.8 mm, Bio-Rad, USA) and a refractive index detector. Samples were eluted with 5 mM H_2_SO_4_ with a flow rate of 0.6 ml/min at 65°C. The cell growth of *S. cerevisiae* was monitored by measuring the optical density of culture broth at a wavelength of 600 nm (OD_600_) by an ultraviolet-visible spectrophotometer. Ethanol yields were determined on the basis of the ratio of actual ethanol produced during fermentation to the theoretical maximum ethanol production calculated from the initial glucose and xylose.

## Notes

View/request a protocol for this paper from *Bio-protocol*.
