# Supplementary material for: Big data mining, rational modification, and ancestral sequence reconstruction inferred multiple xylose isomerases for biorefinery
Source: Sci Adv. 2023 Feb 1;9(5):eadd8835. doi: 10.1126/sciadv.add8835 (PMC9891696; doi:10.1126/sciadv.add8835)
Supplement: Supplementary file 1 — Figs. S1 to S16 Tables S1 to S10 [file sciadv.add8835_sm.pdf]

Science Advances
AAAS

Supplementary Materials for

Big data mining, rational modification, and ancestral sequence reconstruction inferred multiple xylose isomerases for biorefinery

Sitong Chen *et al.*

Corresponding author: Mingjie Jin, jinmingjie@njust.edu.cn

Sci. Adv. **9**, eadd8835 (2023)
DOI: 10.1126/sciadv.add8835

This PDF file includes:

Figs. S1 to S16
Tables S1 to S10

Supplementary Figures

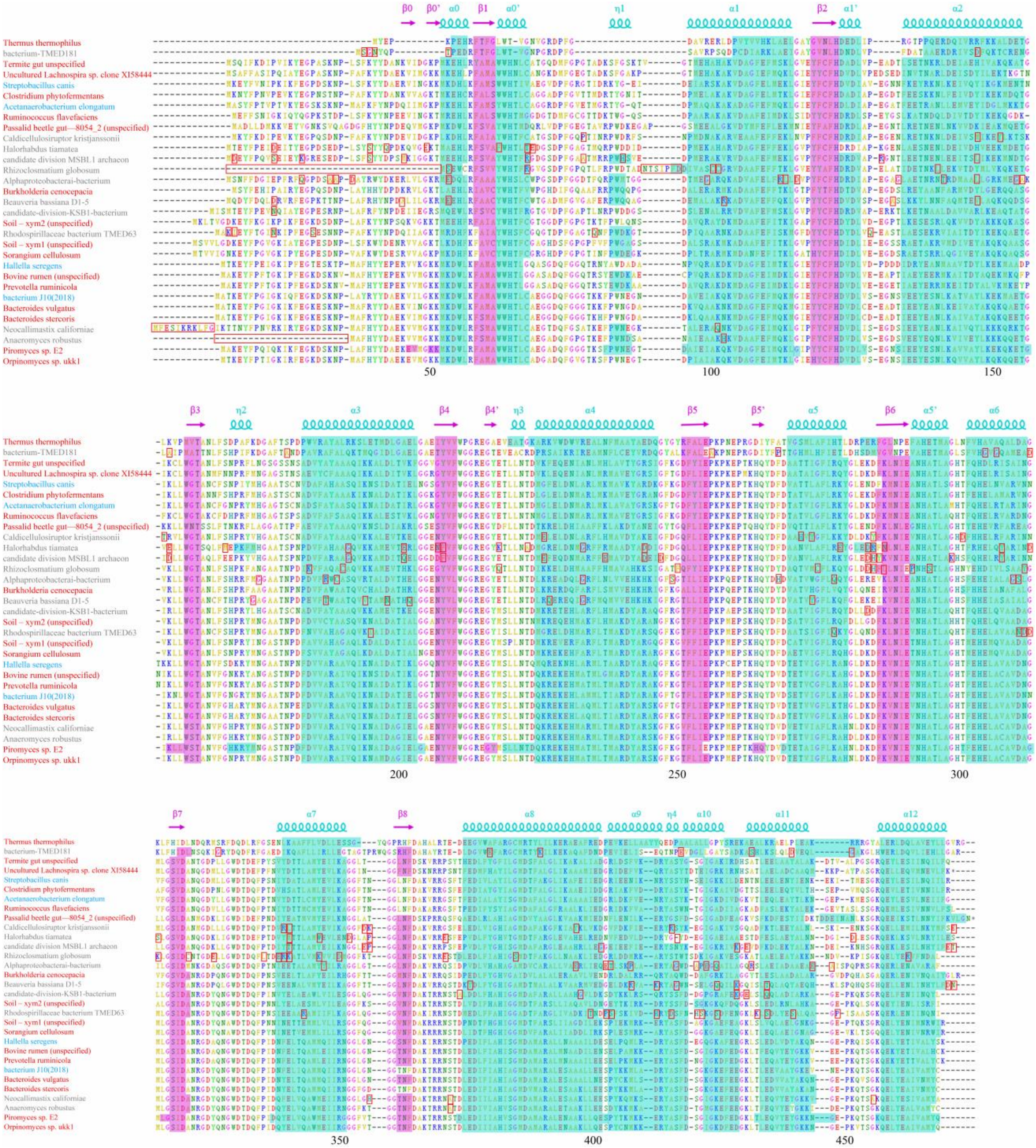

Fig. S1. Sequence alignments for the disclosed 16 XIs active in *S. cerevisiae* and 15 tested XIs in this study. The original hosts for these XIs are shown on the left of the figure. The original hosts for the disclosed 16 active XIs are marked in red; The original hosts for the 4 mined active XIs tested in this study are marked in blue; The original hosts for the 11 inactive XIs tested in this study are marked in grey. The amino acids forming α-helix are indicated with blue background; The amino acids forming β-sheet are indicated with red background; The amino acids of inactive XIs inconsistent with active XIs are marked by red boxes.

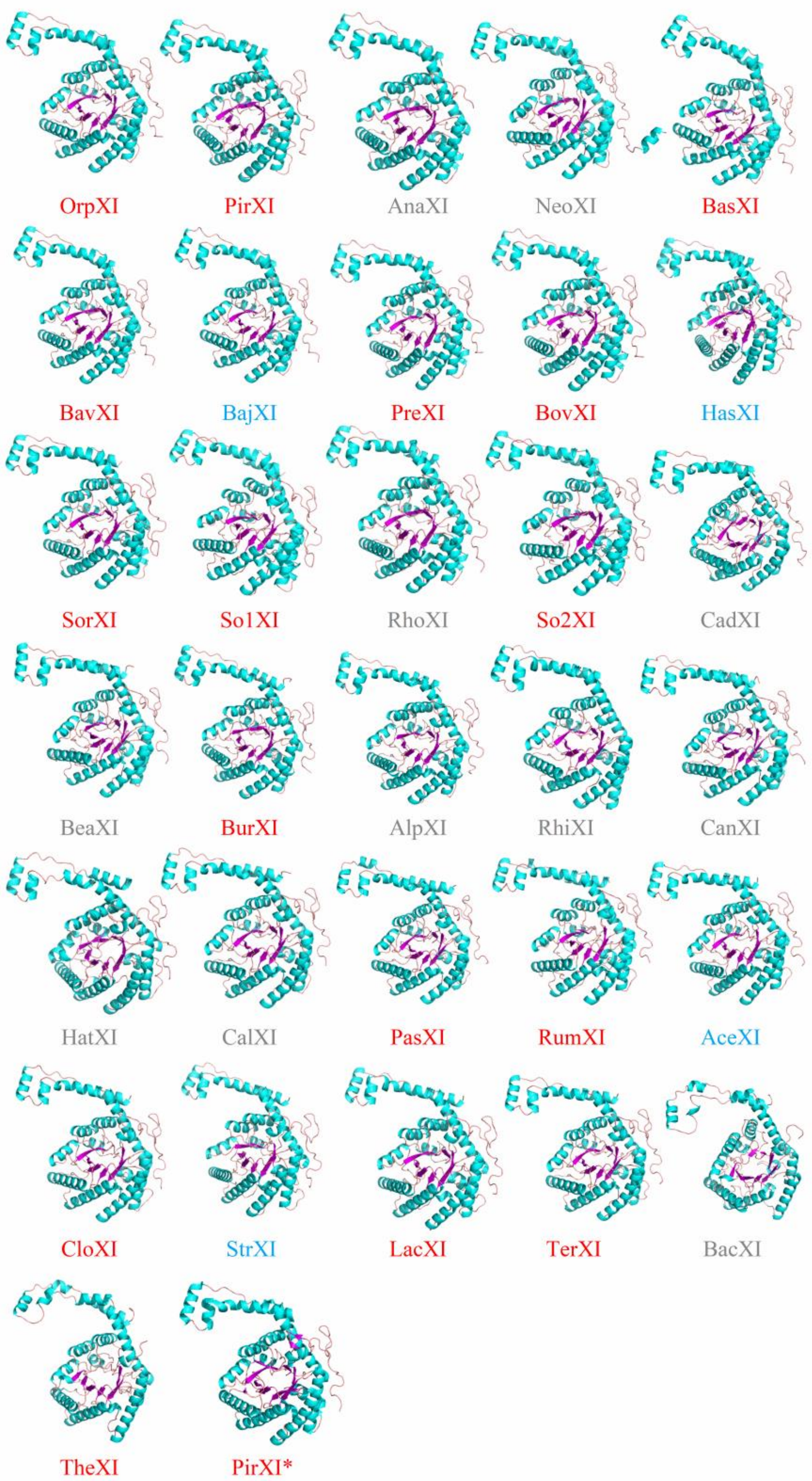

Fig. S2. Predicated protein structures of the disclosed 16 XIs active in *S. cerevisiae* and 15 XIs tested in this study. The names for the disclosed 16 active XIs are marked in red; The names for the 4 active XIs tested in this study are marked in blue; The names for the 11 inactive XIs tested in this study are marked in grey. The structure of PirXI was predicated by AlphaFold v2.0, and the structure of PirXI* (PDB ID: 5NH9) was experimentally determined in a previous study. The similarity of PirXI and PirXI* demonstrated the effectiveness of AlphaFold on XIs structure predication.

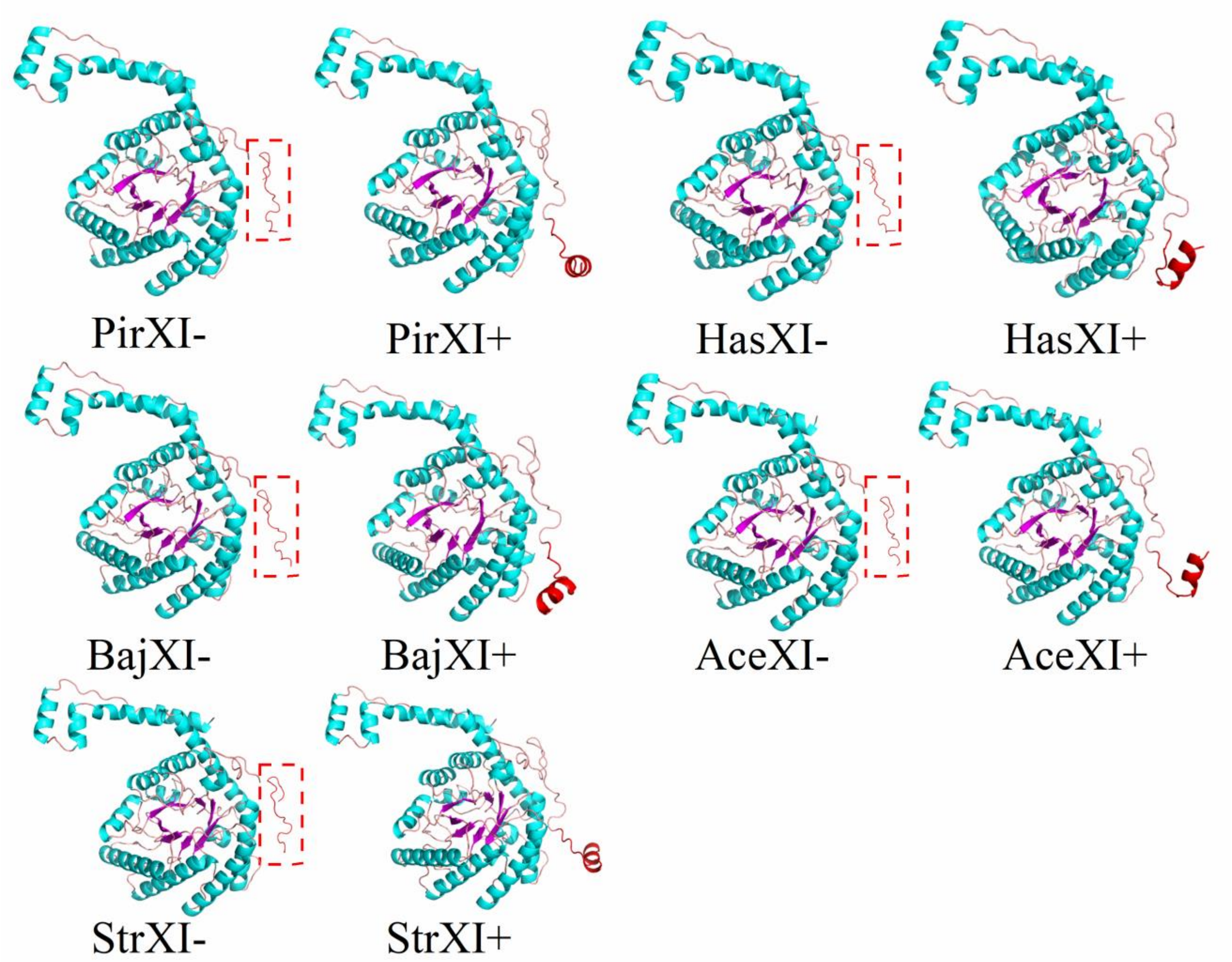

Fig. S3. Modifying active XIs for N-side functional verification in *S. cerevisiae*. Protein structure prediction of modified XIs was done by AlphaFold v2.0 model. The protein structures formed by the altered amino acid sequence are marked in red. Specifically, PirXI+, HasXI+, BajXI+, AceXI+, and StrXI+ were modified by assembling a fragment to their N-terminus; PirXI-, HasXI-, BajXI-, AceXI-, and StrXI- were modified by deleting the fragment at N-terminus, and the deleted sequences are shown in dash lines.

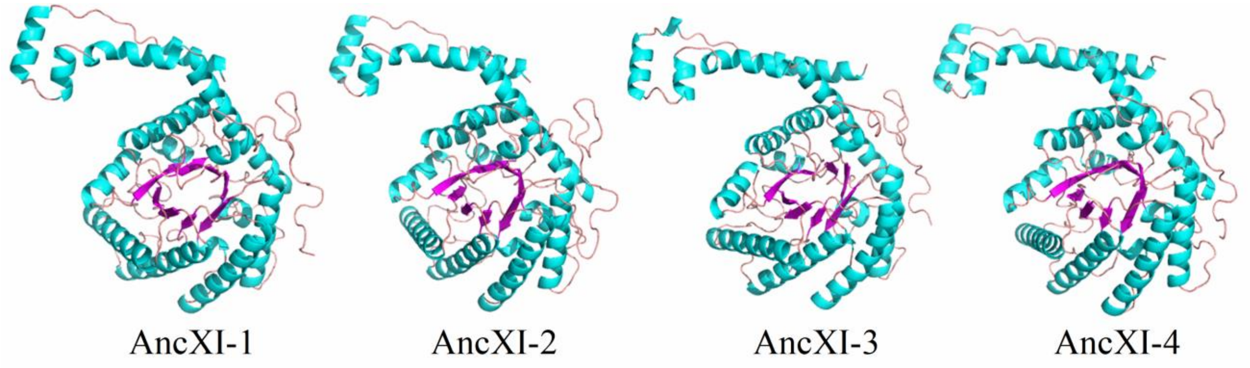

Fig. S4. Predicated protein structures of the ancestral XIs rebuilt in this study.

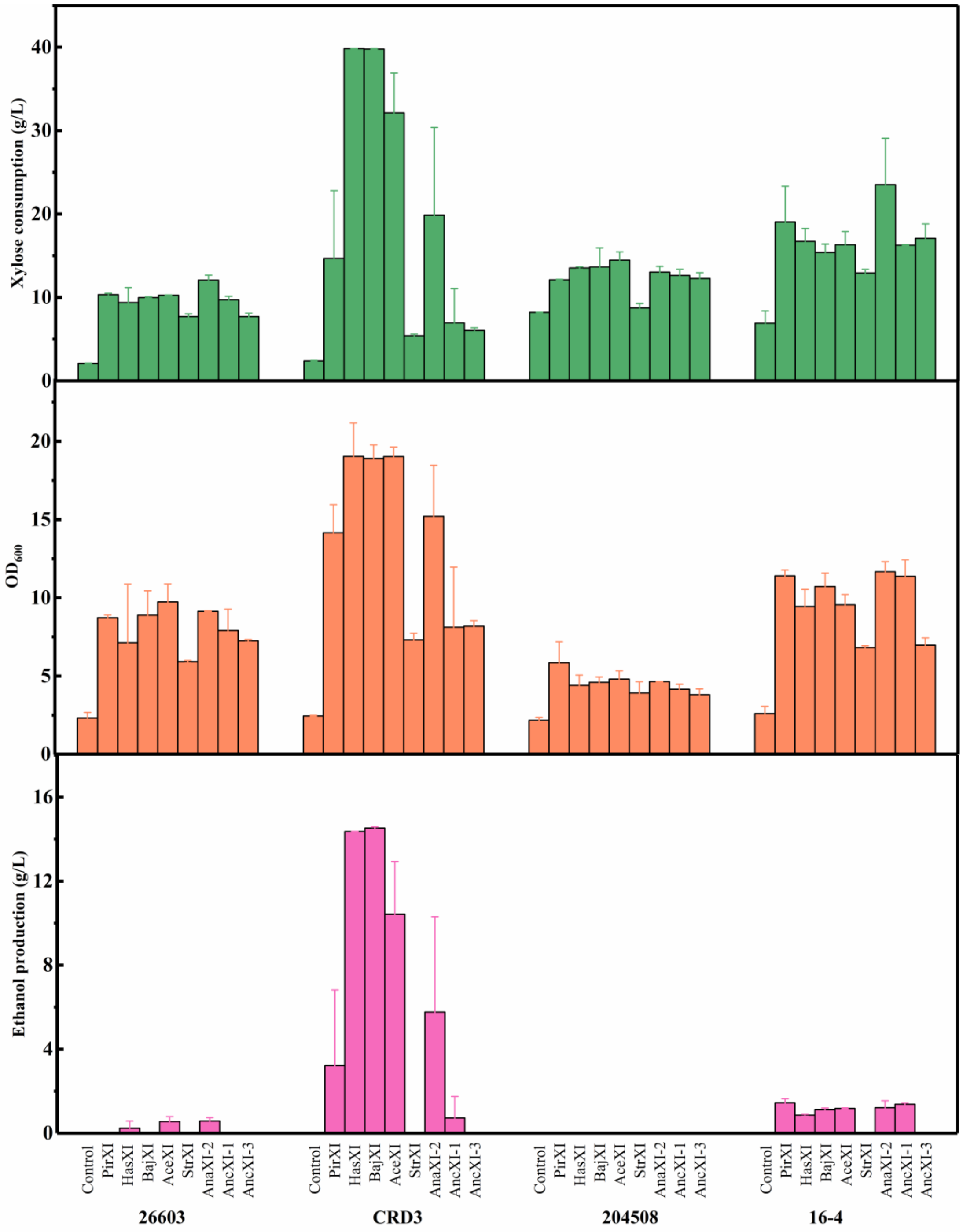

Fig. S5. Expression of verified XIs in *S. cerevisiae* hosts with different genetic backgrounds. Four *S. cerevisiae* strains (ATCC26603, a wild diploid yeast; CRD3, an ATCC26603 derivative with overexpression of the non-oxidative pentose phosphate pathway and some other modifications beneficial to xylose utilization; ATCC 204508, a commonly used haploid yeast; 16-4, a wild diploid yeast isolated by our laboratory, which exhibits high tolerance to many lignocellulosic inhibitors) were applied as the hosts for XIs expression. To limit variation in the copy number of XI gene between strains, the same replicating plasmid pESC-URA was used for XI expression. Recombination strains were cultured in YPX40 medium for 72 h with the conditions of initial OD_{600}=1.0, 30 °C and 150 rpm. Then, samples were withdrawn to determine xylose consumption, cell growth and ethanol production. The data shown in the figure are the mean value ± SD (standard deviation) of two biological replicates.

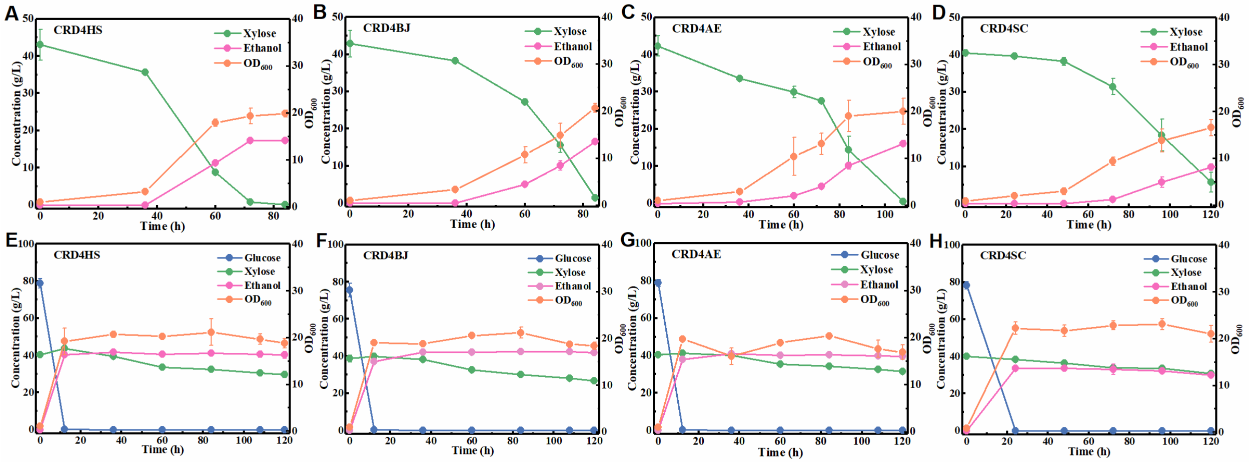

Fig. S6. Fermentation profiles of *S. cerevisiae* strains (XI expression cassettes were inserted into their genomes) with xylose as the main carbon source, or with glucose and xylose as co-carbon source. The evolved strains used here were CRD4HS (**A**, **E**), CRD4BJ (**B**, **F**), CRD4AE (**C**, **G**), and CRD4SC (**D**, **H**). The medium used for A to D was YPX40 medium (10 g/L yeast extract, 20 g/L tryptone, and 40 g/L xylose); The medium used for E to H was YPD80X40 medium (10 g/L yeast extract, 20 g/L tryptone, 80 g/L glucose, and 40 g/L xylose). The data shown in A to H are present as the mean value ± SD (standard deviation) of two biological replicates.

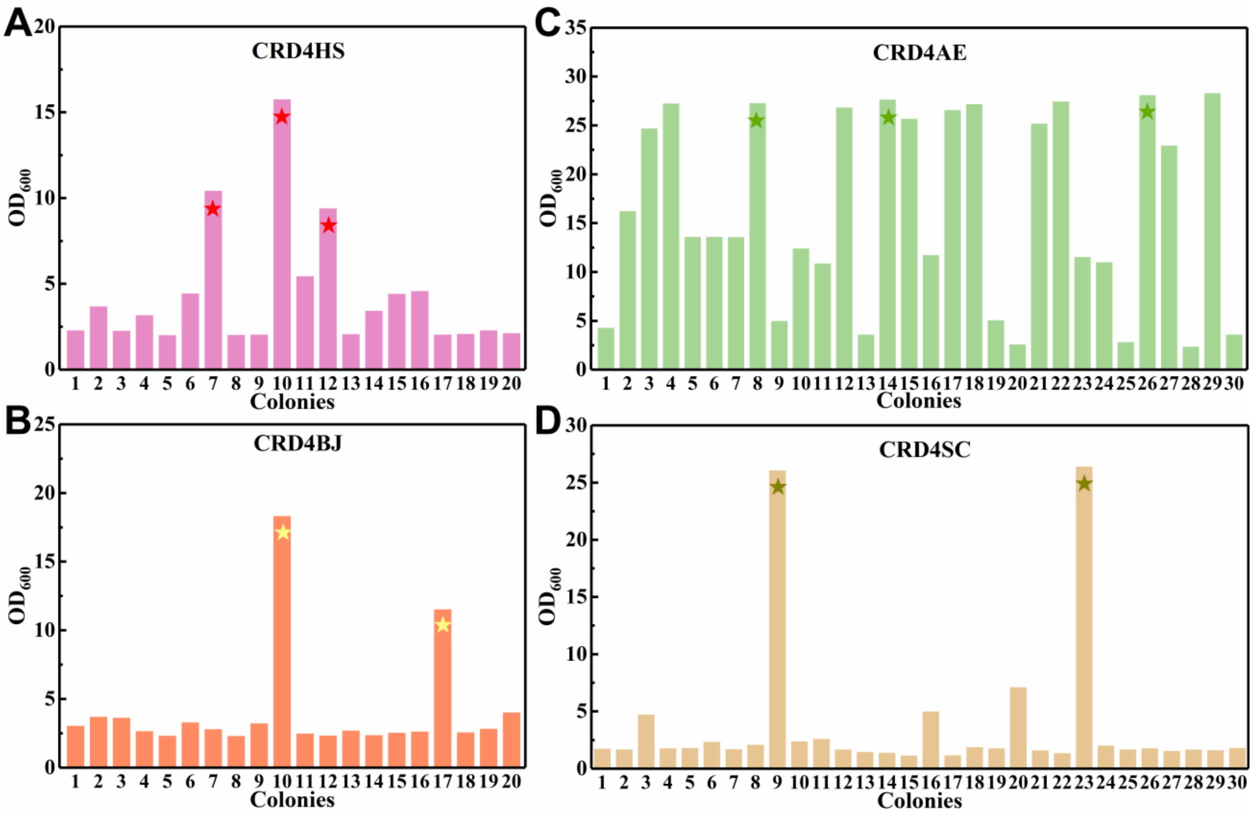

Fig. S7. Screening strains with stable xylose utilization capacity after adaptive laboratory evolution. The screening samples were obtained from the last stage of adaptive laboratory evolution and from the stage with fastest xylose utilization rate. Samples were first cultured in YPD20 medium for five transfers. Thereafter, the obtained broth was plated on YPX40 agar medium and cultured at 30 °C for 48 h. Large colonies were picked to determine their growth capacity on xylose in YPX40 medium (10 g/L yeast extract, 20 g/L tryptone, and 40 g/L xylose).

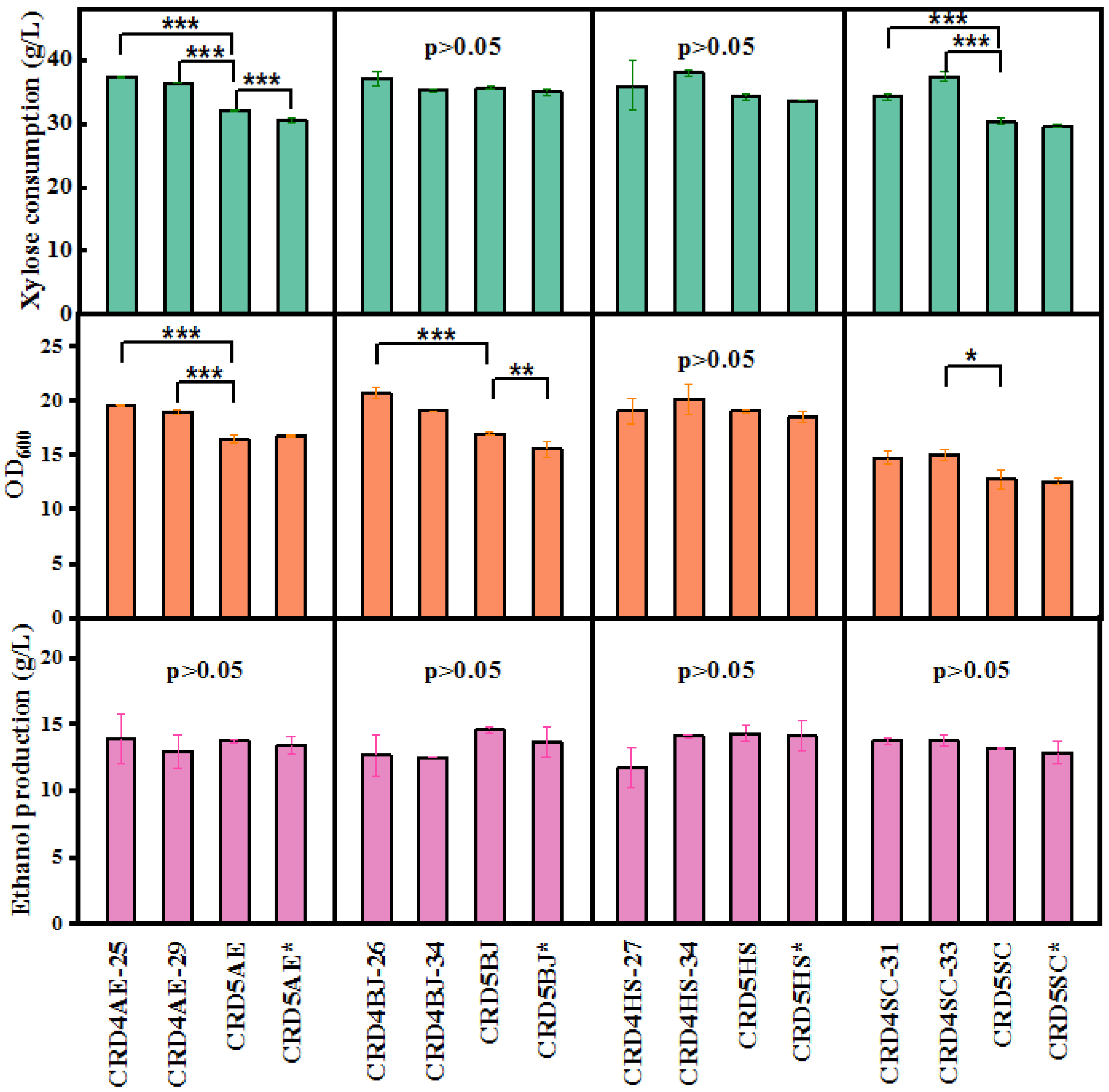

Fig. S8. Fermentation comparison of isolated strains (CRD5HS, CRD5BJ, CRD5AE, and CRD5SC) with populations from the last stage of ALE, populations from the stage with fastest xylose utilization rate, and populations of CRD5AE, CRD5BJ, CRD5HS, and CRD5SC after 5 consecutive transfers in YPD20 medium (CRD5HS*, CRD5BJ*, CRD5AE*, and CRD5SC*). The xylose consumption, cell growth, ethanol production were measured at 14 h in YPX40 medium. Statistical significances were evaluated by one-way ANOVA. ******* represents $p \leq 0.005$, ****** represents $0.005 < p \leq 0.01$, ***** represents $0.01 < p \leq 0.05$. The last numbers in strain names on X-axis represent the ALE stage. The data shown in the figure are the mean value ± SD (standard deviation) of two biological replicates.

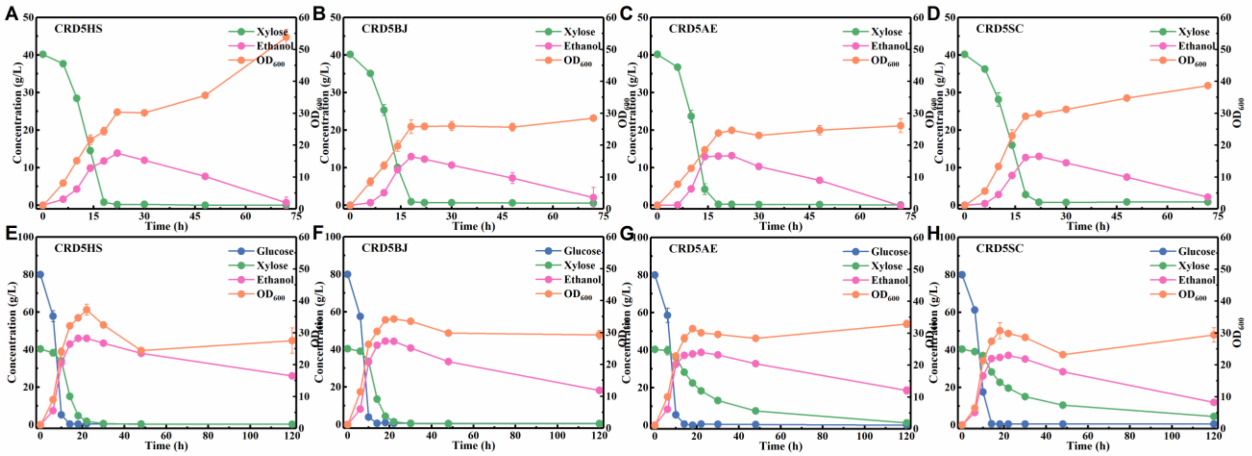

Fig. S9. Fermentation performances of the evolved strains with xylose as the main carbon source, or with glucose/xylose as mixed carbon sources under aerobic conditions. The evolved strains used here were CRD5HS (A, E), CRD5BJ (B, F), CRD5AE (C, G), and CRD5SC (D, H). The medium used for A to D was YPX40 medium (10 g/L yeast extract, 20 g/L tryptone, and 40 g/L xylose); The medium used for E to H was YPD80X40 medium (10 g/L yeast extract, 20 g/L tryptone, 80 g/L glucose, and 40 g/L xylose). The data shown in A to H are presented as the mean value ± SD (standard deviation) of two biological replicates.

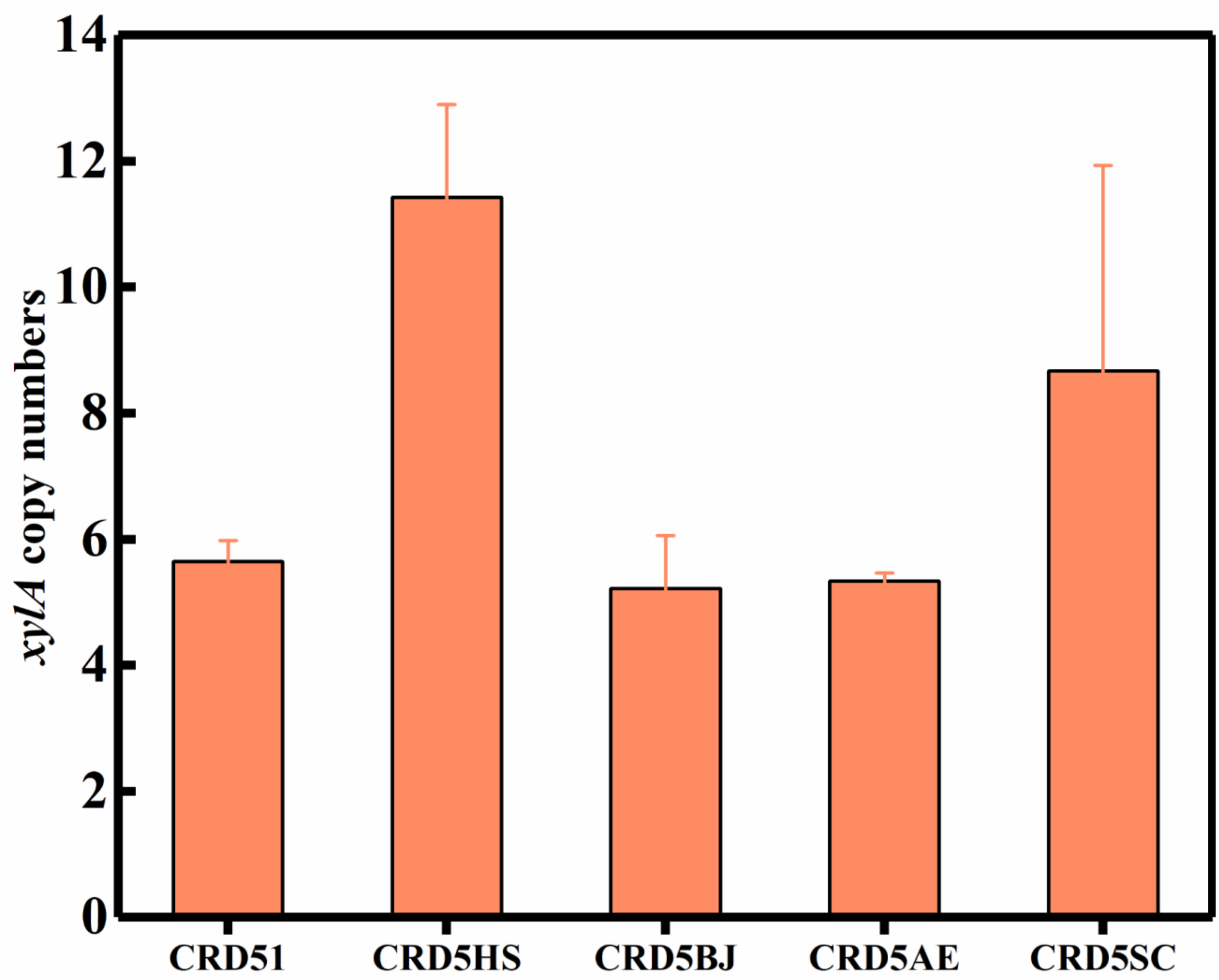

Fig. S10. Copy numbers of *Pir-xylA*, *Has-xylA*, *Baj-xylA*, *Ace-xylA*, and *Str-xylA* in CRD51, CRD5HS, CRD5BJ, CRD5AE and CRD5SC srtains.

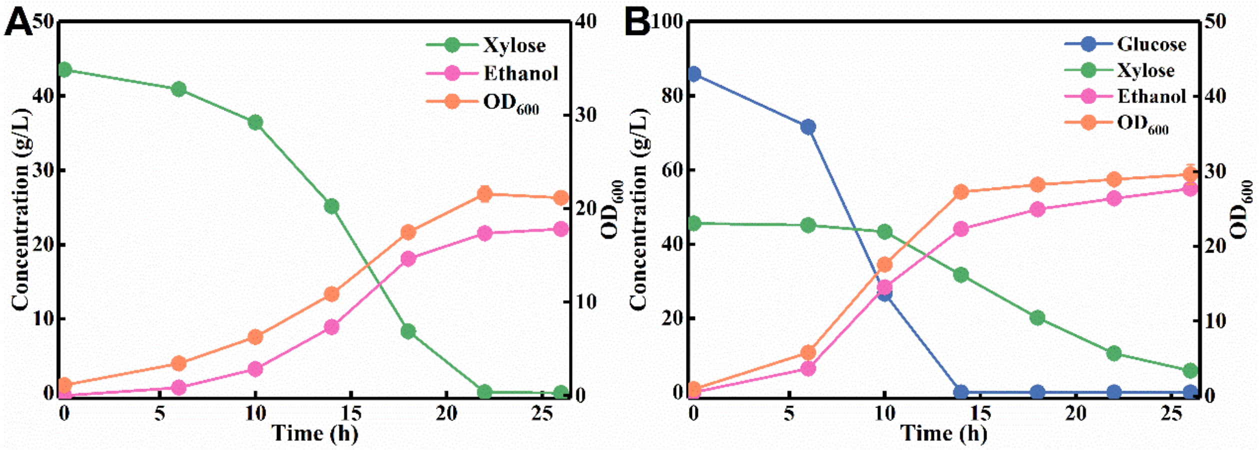

Fig. S11. Fermentation performances of CRD51 expressing PirXI with xylose as the main carbon source, or with glucose and xylose as the main co-carbon source. (A) YPX40 medium (10 g/L yeast extract, 20 g/L tryptone, and 40 g/L xylose) was used for fermentation; (B) YPD80X40 medium (10 g/L yeast extract, 20 g/L tryptone, 80 g/L glucose, and 40 g/L xylose) was used for fermentation. The data shown in A and B are present as the mean value ± SD (standard deviation) of two biological replicates.

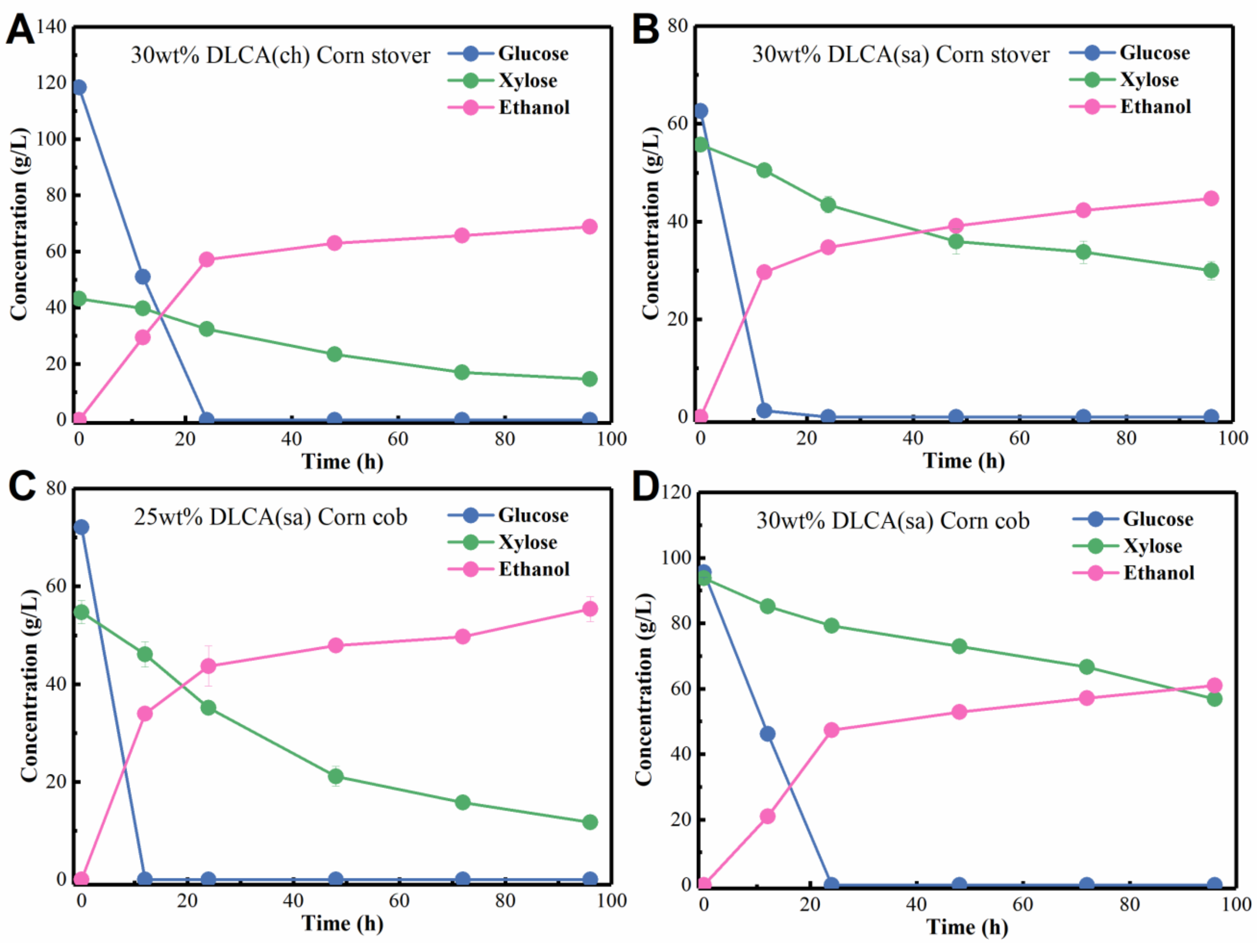

Fig. S12. Batch fermentation performances of *S. cerevisiae* CRD51 in different kinds of hydrolysates. (A) 30wt.% DLCA(ch) pretreated corn stover; (B) 30wt.% DLCA(sa) pretreated corn stover; (C) 25wt.% DLCA(sa) pretreated corn cob; (D) 30wt.% DLCA(sa) pretreated corn cob. The data shown in A to D are presented as the mean value ± SD (standard deviation) of two biological replicates.

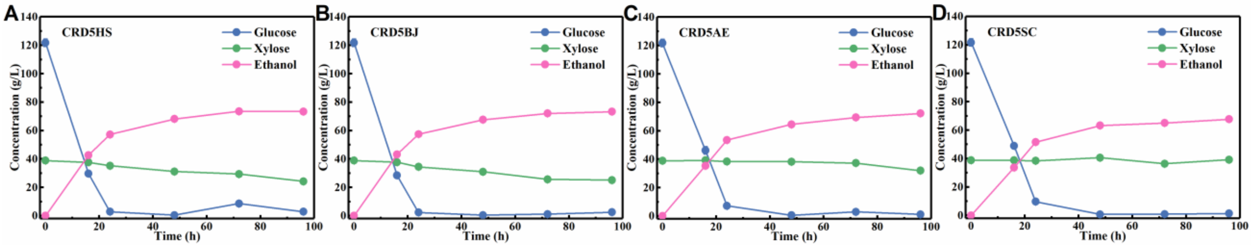

Fig. S13. Batch fermentation in 35wt.% DLCA(ch) corn stover hydrolysate. Fermentation strains were CRD5HS (**A**), CRD5BJ (**B**), CRD5AE (**C**), and CRD5SC (**D**). DLCA(ch) corn stover were hydrolyzed at 35wt.% for 22 h and the resulted hydrolysate was used for fermentation. The data shown in A to D are presented as the mean value ± SD (standard deviations) of two biological replicates.

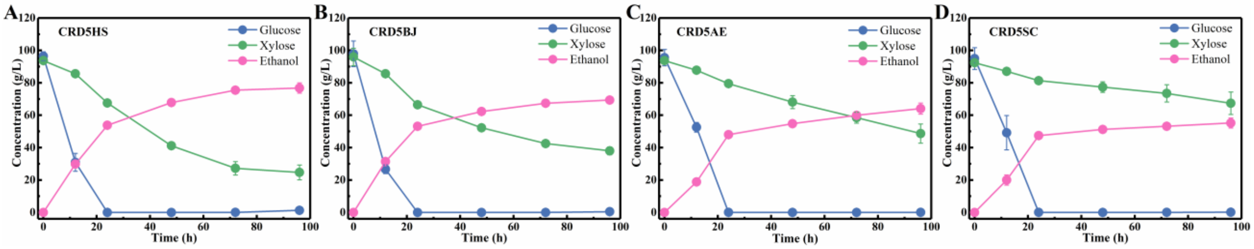

Fig. S14. Batch fermentation in 30wt.% DLCA(sa) corn cob hydrolysate. Fermentation strains were CRD5HS (**A**), CRD5BJ (**B**), CRD5AE (**C**), and CRD5SC (**D**). DLCA(sa) corn cob were hydrolyzed at 30wt.% for 72 h and the resulted hydrolysate was used for fermentation. The data shown in A to D are presented as the mean value ± SD (standard deviations) of two biological replicates.

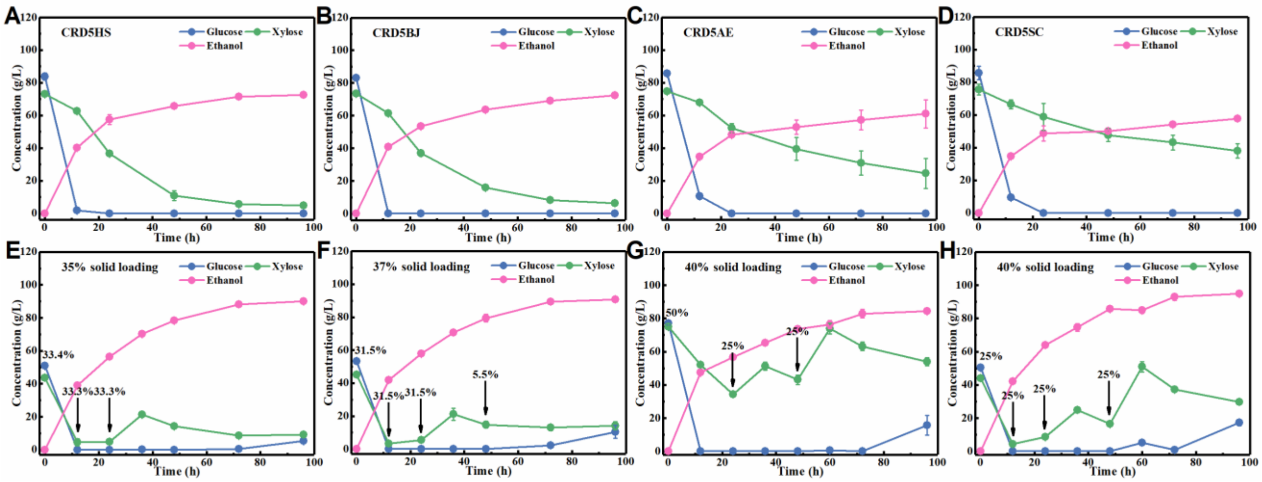

Fig. S15. Batch and fed-batch fermentation with DLCA(sa) corn cob as feedstock. For batch fermentation, DLCA(sa) corn cob was hydrolyzed at 25wt.% for 72 h and then CRD5HS (**A**), CRD5BJ (**B**), CRD5AE (**C**), and CRD5SC (**D**) were inoculated for fermentation. For fed-batch fermentation, different fed-batch operations were applied with CRD5HS as the fermentation strain. (**E**) DLCA(sa) corn cob was applied at a total solid loading of 35wt.%. In this scenario, 33.4% corn cob and cellulase were added at the initial time for prehydrolysis of 12 h. Thereafter, the remaining corn cob and cellulase were added to the fermentation system at 12 and 24 h. (**F**) DLCA(sa) corn cob was applied at a total solid loading of 37wt.%. In this scenario, 31.5% corn cob and cellulase were added at the initial time for prehydrolysis of 12 h. Thereafter, the remaining corn cob and cellulase were added during fermentation with 31.5%, 31.5%, and 5.5% of corn cob and cellulase fed at 12, 24, and 48 h, respectively. Two fed-batch operation modes were applied for 40wt.% solid loading scenario. Mode 1 (**G**): 50% corn cob and cellulase were added for prehydrolysis of 12 h. The remaining corn cob and cellulase were equally added into the fermentation system at 24 and 48 h; Mode 2 (**H**): 25% of the corn cob and cellulase were added for pre-hydrolysis of 12 h. The remaining corn cob and cellulase were equally added into the fermentation system at 12, 24 and 48 h. The data shown in A to H are presented as the mean value ± SD (standard deviation) of two biological replicates.

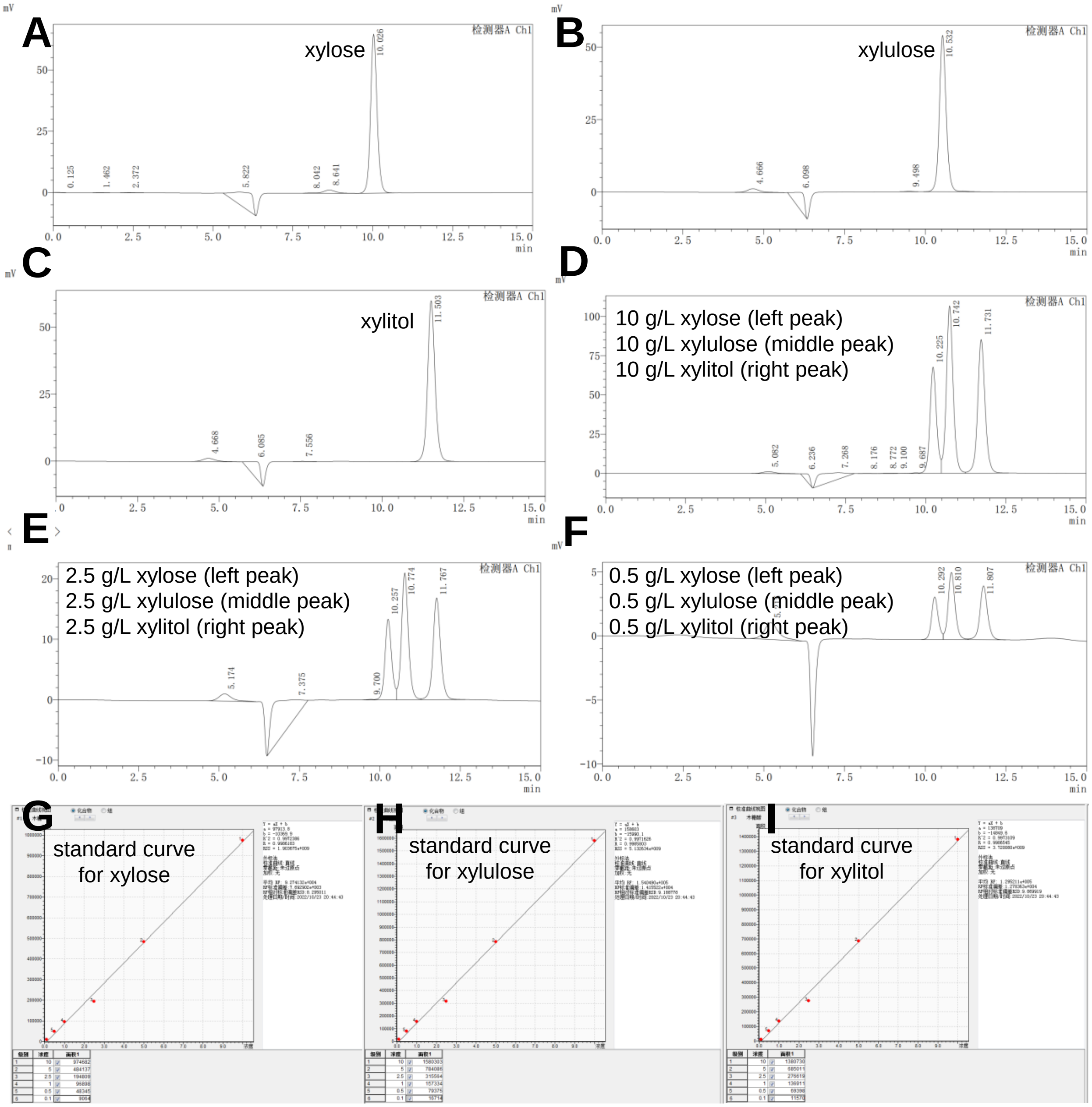

Fig. S16. The chromatograms and standard curves of xylose, xylulose, and xylitol. The chromatograms of single xylose (A); the chromatograms of single xylulose (B); the chromatograms of single xylitol (C); the chromatograms of the mixtures of xylose, xylulose, and xylitol (D, E, F); the standard curves of xylose (G), xylulose (H), xylitol (I). The chromatograms suggest that xylose, xylulose and xylitol can be well separated by HPX-87H column. The standard curves of xylose, xylulose and xylitol made from the mixed standard substances all exhibit good linear correlation, indicating that it is practicable to quantify the xylose, xylulose and xylitol in determining XI activity.

Supplementary Tables

Table S1. Strains and plasmids used in this study.

Strains or plasmids	Descriptions	Sources
E. coli DH5α	A host strain for the amplification of constructed plasmids	Preserved in our laboratory
S. cerevisiae ATCC26603	Diploid *S. cerevisiae* strain	Purchased from Guangdong Institute of Microbiology
S. cerevisiae CRD3	ATCC 26603, △*GRE3*, *PHO13::TPI1p-XKS1-ADH1t-FBA1p-TKL1-FBA1t-PGK1p-RKI1-GAL2t*, *pyk2::TEF1p-GAL2*^{N376F}*-TEF1t-TDH3p-TAL1-PGI1t*	This study
S. cerevisiae CRD3PE	CRD3 harboring pESC-pir	This study
S. cerevisiae CRD3AE	CRD3 harboring pESC-ace	This study
S. cerevisiae CRD3BJ	CRD3 harboring pESC-baj	This study
S. cerevisiae CRD3HS	CRD3 harboring pESC-has	This study
S. cerevisiae CRD3SC	CRD3 harboring pESC-str	This study
S. cerevisiae CRD3AR	CRD3 harboring pESC-ana	This study
S. cerevisiae CRD3NC	CRD3 harboring pESC-neo	This study
S. cerevisiae CRD3HT	CRD3 harboring pESC-hat	This study
S. cerevisiae CRD3CD	CRD3 harboring pESC-can	This study
S. cerevisiae CRD3BB	CRD3 harboring pESC-bea	This study
S. cerevisiae CRD3RG	CRD3 harboring pESC-rhi	This study
S. cerevisiae CRD3CK	CRD3 harboring pESC-cal	This study
S. cerevisiae CRD3RB	CRD3 harboring pESC-rho	This study
S. cerevisiae CRD3CA	CRD3 harboring pESC-cad	This study
S. cerevisiae CRD3AL	CRD3 harboring pESC-alp	This study
S. cerevisiae CRD3BA	CRD3 harboring pESC-bac	This study
S. cerevisiae 26603-AR1	26603 harboring pESC-ana1	This study
S. cerevisiae 26603- AR2	26603 harboring pESC-ana2	This study
S. cerevisiae 26603-NC1	26603 harboring pESC-neo1	This study
S. cerevisiae 26603-NC2	26603 harboring pESC-neo2	This study
S. cerevisiae 26603-RB1	26603 harboring pESC-rho1	This study
S. cerevisiae 26603-RG1	26603 harboring pESC-rhi1	This study
S. cerevisiae 26603-CK1	26603 harboring pESC-cal1	This study
S. cerevisiae 26603-BB1	26603 harboring pESC-bea1	This study
S. cerevisiae 26603-AN1	26603 harboring pESC-anc1	This study
S. cerevisiae 26603- AN2	26603 harboring pESC-anc2	This study
S. cerevisiae 26603- AN3	26603 harboring pESC-anc3	This study
S. cerevisiae 26603- AN4	26603 harboring pESC-anc4	This study
S. cerevisiae CRD41	CRD3, *delta::TDH3p-pirxylA*(codon optimization for *S. cerevisiae*)-*CYC1t*	This study
S. cerevisiae CRD4HS	CRD3, *delta::TDH3p-hasxylA*(codon optimization for *S. cerevisiae*)-*CYC1t*	This study
S. cerevisiae CRD4BJ	CRD3, *delta::TDH3p-bajxylA*(codon optimization for *S. cerevisiae*)-*CYC1t*	This study
S. cerevisiae CRD4AE	CRD3, *delta::TDH3p-acexylA*(codon optimization for *S. cerevisiae*)-*CYC1t*	This study
S. cerevisiae CRD4SC	CRD3, *delta::TDH3p-strxylA*(codon optimization for *S. cerevisiae*)-*CYC1t*	This study
S. cerevisiae CRD51	CRD41, adaptive evolution in xylose	This study
S. cerevisiae CRD5HS	CRD4HS, adaptive evolution in xylose	This study
S. cerevisiae CRD5BJ	CRD4BJ, adaptive evolution in xylose	This study
S. cerevisiae CRD5AE	CRD4AE, adaptive evolution in xylose	This study
S. cerevisiae CRD5SC	CRD4SC, adaptive evolution in xylose	This study
pESC-URA	High copy vector expressed in *S. cerevisiae*	Provided by GenScript
G418_pESC-URA	pESC-URA derivative containing G418 resistance gene	This study
TDH3_G418_pESC-URA	G418_pESC-URA derivative containing *S. cerevisiae* TDH 3 promoter	This study
pESC-ace	TDH3_G418_pESC-URA derivative containing codon optimized *acexylA* gene	This study
pESC-baj	TDH3_G418_pESC-URA derivative containing codon optimized *bajxylA* gene	This study
pESC-has	TDH3_G418_pESC-URA derivative containing codon optimized *hasxylA* gene	This study
pESC-str	TDH3_G418_pESC-URA derivative containing codon optimized *strxylA* gene	This study
pESC-ana	TDH3_G418_pESC-URA derivative containing codon optimized *anaxylA* gene	This study
pESC-neo	TDH3_G418_pESC-URA derivative containing codon optimized *neoxylA* gene	This study

pESC-hat	TDH3_G418_pESC-URA derivative containing codon optimized *hatxylA* gene	This study
pESC-can	TDH3_G418_pESC-URA derivative containing codon optimized *canxylA* gene	This study
pESC-bea	TDH3_G418_pESC-URA derivative containing codon optimized *beaxylA* gene	This study
pESC-rhi	TDH3_G418_pESC-URA derivative containing codon optimized *rhixylA* gene	This study
pESC-cal	TDH3_G418_pESC-URA derivative containing codon optimized *calxylA* gene	This study
pESC-rho	TDH3_G418_pESC-URA derivative containing codon optimized *rhoxylA* gene	This study
pESC-cad	TDH3_G418_pESC-URA derivative containing codon optimized *cadxylA* gene	This study
pESC-alp	TDH3_G418_pESC-URA derivative containing codon optimized *alpxylA* gene	This study
pESC-bac	TDH3_G418_pESC-URA derivative containing codon optimized *bacxylA* gene	This study
pESC-ana1	TDH3_G418_pESC-URA derivative containing *ana1xylA* gene	This study
pESC-ana2	TDH3_G418_pESC-URA derivative containing *ana2xylA* gene	This study
pESC-neo1	TDH3_G418_pESC-URA derivative containing *neo1xylA* gene	This study
pESC-neo2	TDH3_G418_pESC-URA derivative containing *neo2xylA* gene	This study
pESC-rho1	TDH3_G418_pESC-URA derivative containing *rho1xylA* gene	This study
pESC-rhi1	TDH3_G418_pESC-URA derivative containing *rhi1xylA* gene	This study
pESC-cal1	TDH3_G418_pESC-URA derivative containing *cal1xylA* gene	This study
pESC-bea1	TDH3_G418_pESC-URA derivative containing *bea1xylA* gene	This study
pESC-pir+	TDH3_G418_pESC-URA derivative containing *pir+xylA* gene	This study
pESC-pir-	TDH3_G418_pESC-URA derivative containing *pir-xylA* gene	This study
pESC-has+	TDH3_G418_pESC-URA derivative containing *has+xylA* gene	This study
pESC-has-	TDH3_G418_pESC-URA derivative containing *has-xylA* gene	This study
pESC-baj+	TDH3_G418_pESC-URA derivative containing *baj+xylA* gene	This study
pESC-baj-	TDH3_G418_pESC-URA derivative containing *baj-xylA* gene	This study
pESC-ace+	TDH3_G418_pESC-URA derivative containing *ace+xylA* gene	This study
pESC-ace-	TDH3_G418_pESC-URA derivative containing *ace-xylA* gene	This study
pESC-str+	TDH3_G418_pESC-URA derivative containing *str+xylA* gene	This study
pESC-str-	TDH3_G418_pESC-URA derivative containing *str-xylA* gene	This study
pESC-anc1	TDH3_G418_pESC-URA derivative containing codon optimized *anc1xylA* gene	This study
pESC-anc2	TDH3_G418_pESC-URA derivative containing codon optimized *anc2xylA* gene	This study
pESC-anc3	TDH3_G418_pESC-URA derivative containing codon optimized *anc3xylA* gene	This study
pESC-anc4	TDH3_G418_pESC-URA derivative containing codon optimized *anc4xylA* gene	This study
pML104	CRISPR–Cas9 genome editing vector for *S. cerevisiae*	Purchased from MiaoLingBio
pML-G418	pML104 containing G418 resistance gene	This study
PML-delta	pML-G418 containing 20mer guide sequence targeting the yeast delta locus	This study
pML-has	pML-delta derivative containing codon optimized *hasxylA* gene	This study
pML-baj	pML-delta derivative containing codon optimized *bajxylA* gene	This study
pML-ace	pML-delta derivative containing codon optimized *acexylA* gene	This study
pML-str	pML-delta derivative containing codon optimized *strxylA* gene	This study

Table S2. **Primers used in this study.**

Primer	Sequence (5'-3')
**For construction of PML-delta**	
G418-F	gtcgacggtatcgataagcttcgttttcgacactggatggc
G418-R	actgactagtagactgaattcctagagatctgtttagcttgcctcg
delta-F	acgagtactttaccgtgtacaacgagctgaccaaggtgaaatatg
delta-R	gcagtgaaagataaatgatctatactagaagttctcctcggttttagagctagaaat
**For construction of plasmids containing XI gene and homologation arm of delta**	
delta-P1-F	tgttggaatagaaatcaactatcatctact
delta-P1-R	gactcgaggatataggaatcctcaaaa
TDH3p-F	gattcctatatcctcgagtcattatcaatactgccatttcaaag
CYC1t-R	agaagttctccttcgagcgtcccaaaacc
delta-P2-F	acgctcgaaggagaacttctagtatattctgtatacctaatattatagc
delta-P2-R	tggaagctgaaacgtctaacgg
**For construction of plasmids containing modified inactive XI genes**	
ana-1F	acataaacaaacaaaatggctaaagaatacttcccacaaatccaaaagattaaatttgagggtaaagattctaaaaatccattagc tttccattactacgatgctgaa
ana-1R	tcggttagagcggattcaacagtacatagcgaccaaagc
ana-2R	actttttgcttagcggcttcaatagcattagc
ana-2F	gccgctaagcaaaaagttgatgctgccttcgaa
ana-3R	gtctgtggagtttcttctggtcttagcgtcga
ana-3F	gaagaaactccacagacttggaagatattgttattgctc
cal-1F	acataaacaaacaaaatgaagtacttcaaggatatcccaga
cal-2R	accaccgttatcgaaaccaccagctttaataact
cal-2F	gtttcgataacggtggtttgaacttcgatgca
cal-3R	ccaacttagcagcgattttaaaacccttagcg
cal-3F	aatcgctgctaagttggttaaggatggtgttttcg
cal-4R	gaatgtagcttgacctgaaacgatctttgcac
cal-4F	caggtcaagctacattcaagacattggaagaatacgct
cal-1R	tcggttagagcggatttacaatgaaaacatgtacttattcaaaatcattt
neo-1F	acataaacaaacaaaatggctaaagaatacttcccaaacgttagaaagatcag
neo-1R	tcggttagagcggattcaagcgtacatagcgacaatgg
neo-2R	ccttgttcttagcacgctctagagcagtcttacc
neo-2F	gcgtgctaagaacaaggttgacgctggtttcg
neo-3R	taccaccagtacccaaaccaccatttctgatg
neo-3F	tttgggtactggtggtactaacttcgatgctaagac
neo-4R	tcggttagagcggattcaagcgtacatagcgacaatggattcgtataattcttgcttaccagaagtttgctttggttcaccg
rho-1F	acataaacaaacaaaatggctaagatcgaatacttcacagg
rho-2R	acatacctgcatctgcagcaacagctaattcatgtt
rho-2F	tgcagatgcaggtatgttgggttctattgatgcaaata
rho-3R	tcgtccaagatcttatcagcaacgatcaaacc
rho-3F	gctgataagatcttggacgaatctgaattgcca
rho-1R	tcggttagagcggattcatttggagtacaaagcgacaa
rhi-1F	acataaacaaacaaaatgactaaagaatacttcccagaaatcg
rhi-2R	tacaccattccttcatcttcttacccttaacaactctt
rhi-2F	gaagatgaaggaatggtgtagattttcagttgtttactg
rhi-3R	tcatctgcagtatcccatggtctaaac
rhi-3F	ccatgggatactgcagatgatattgttgcttcatgttgtggtag
rhi-1R	tcggttagagcggatttataaagcatcattaaaaactctttcg
**For construction of plasmids containing modified active XI genes**	
PE--F	acataaacaaacaaaatggcattccattactacgatgc
PE+F	acataaacaaacaaaatgttcgaatcaataaagagaaagttattcggtatcaaaacaaccaactacttcccacaaatccaaaaga ttaa
PE-R	ttagagcggatcttagctagcttattggtacatagcaacaattgctt

HS--F	acataaacaaacaaaatggctttccactactacgaacca
HS+F	acataaacaaacaaaatgttcgaatcaataaagagaaagttattcggtatcaaaacaaccaactacttcccagaaatcggcaaga
HS-R	ttagagcggatcttagctagctcatttggagtacaaagcgacaa
BJ--F	acataaacaaacaaaatggcttacagatactacgatgccg
BJ+F	acataaacaaacaaaatgttcgaatcaataaagagaaagttattcggtatcaaaacaaccaactatttcccaggtattggtaagatt ca
BJ-R	ttagagcggatcttagctagctcaacagtacatagcgacaatagctt
AE--F	acataaacaaacaaaatggctttcaagttctacaaccca
AE+F	acataaacaaacaaaatgttcgaatcaataaagagaaagttattcggtatcaaaacaaccaactacttcccaactgttccaactgt c
AE-R	ttagagcggatcttagctagctcagccgaagatgatttcgttc
SC--F	acataaacaaacaaaatgtctttcaagtactacgacgctg
SC+F	acataaacaaacaaaatgttcgaatcaataaagagaaagttattcggtatcaaaacaaccaactatttcgtcaacatcccaaagat t
SC-R	ttagagcggatcttagctagctcaacgcaaaatgatttggttc
**For measuring XI gene copy numbers**	
RTACT1-F	atgcaaaccgctgctcaa
RTACT1-R	agtttggtcaataccggcaga
RTPIR-F	gaacggtgcttcaactaatcc
RTPIR-R	gccattgtcaacatagtagccata
RTHAS-F	aaccaactaagcaccaatacg
RTHAS-R	gcgtcgatagaacccaacata
RTBAJ-F	acttggtctcagagggtaactcc
RTBAJ-R	ttagtggcagcaccgttcat
RTACE-F	attgctccagagggtgctac
RTACE-R	gcgtaagcgaaagagtcagc
RTSTR-F	acactgaccaattcccatcc
RTSTR-R	caaaggtgtccatacccaag

Table S3. The information of 16 disclosed XIs active in *S. cerevisiae* and 15 XIs tested in this study.

Types	XIs	GenBank	Host strain	Superkingdom	Phylum	Length
16 disclosed active XIs in *S. cerevisiae*	TheXI	BAA14301	*Thermus thermophilus*	*Bacteria*	*Deinococcus-Thermus*	387
	PirXI	CAB76571	*Piromyces* sp. E2	*Eukaryotes*	*Eukaryotes*	437
	OrpXI	ACA65427	*Orpinomyces* sp. ukk1	*Eukaryotes*	*Eukaryotes*	437
	CloXI	BBG40462	*Clostridium phytofermentans* ISDg	*Bacteria*	*Firmicutes*	438
	So1XI	AEG75765	Soil – xym1 (unspecified)	*Bacteria*	Environmental samples	443
	So2XI	AEG75766	Soil – xym2 (unspecified)	*Bacteria*	Uncultured bacterium	442
	BasXI	AEK21499	*Bacteroides stercoris*	*Eukaryotes*	*Eukaryotes*	438
	RumXI	CAB51938	*Ruminococcus flavefaciens*	*Bacteria*	*Firmicutes*	438
	PreXI	AGL34957	*Prevotella ruminicola*	*Bacteria*	*Bacteroidetes*	439
	BurXI	CAR57287	*Burkholderia cenocepacia*	*Bacteria*	*Proteobacteria*	440
	BavXI	ABR41556	*Bacteroides vulgatus*	*Bacteria*	*Bacteroidetes*	438
	BovXI	AEL74969	Bovine rumen (unspecified)	*Bacteria*	Environmental samples	439
	SorXI	WP_020464968	*Sorangium cellulosum*	*Bacteria*	*Proteobacteria*	443
	TerXI	HV438106	Termite gut (unspecified)	*Bacteria*	*Firmicutes*	439
	LacXI	MK355208.1	Uncultured *Lachnospira* sp. clone XI58444 xylose isomerase gene, complete cds	*Bacteria*	*Firmicutes*	440
	PasXI	MT846924	Passalid beetle gut—8054_2 (unspecified)	*Bacteria*	Environmental samples	436
15 XIs tested in this study	AceXI	WP_092638076	*Acetanaerobacterium elongatum*	*Bacteria*	*Firmicutes*	437
	BajXI	RLT77382	bacterium J10(2018)	*Bacteria*	Unclassified Bacteria	438
	HasXI	WP_027952460	*Hallella seregens*	*Bacteria*	*Bacteroidetes*	439
	StrXI	WP_156299687	*Streptobacillus canis*	*Bacteria*	*Proteobacteria*	443
	AnaXI	ORX78688	*Anaeromyces robustus*	*Eukaryotes*	*Eukaryotes*	415
	NeoXI	ORY81692	*Neocallimastix californiae*	*Eukaryotes*	*Eukaryotes*	449
	HatXI	WP_008523802.1	*Halorhabdus tiamatea*	*Archaea*	*Archaea*	438
	CanXI	KXA98896.1	candidate division MSBL1 archaeon SCGC-AAA259J03	*Archaea*	*Archaea*	439
	BeaXI	KGQ13041.1	*Beauveria bassiana* D1-5	*Eukaryotes*	*Eukaryotes*	440
	RhiXI	ORY49883.1	*Rhizoclosmatium globosum*	*Eukaryotes*	*Eukaryotes*	407
	CalXI	WP_013433194.1	*Caldicellulosiruptor kristjanssonii*	*Bacteria*	*Firmicutes*	438
	RhoXI	RPF97854.1	*Rhodospirillaceae bacterium* TMED63	*Bacteria*	*Proteobacteria*	438
	CadXI	MCA9730992.1	candidate division KSB1 bacterium	*Bacteria*	Unclassified Bacteria	439
	AlpXI	RYY14481.1	*Alphaproteobacteria* bacterium	*Bacteria*	*Proteobacteria*	437
	BacXI	OUW45276.1	bacterium TMED181	*Bacteria*	Unclassified Bacteria	391

Table S4. Modifying inactive XI sequences for active expression in *S. cerevisiae*.

XIs	Modified amino acid sequences
AnaXI-1	Deleting M1, inserting MAKEYFPQIQKIKFEGKDSKNPL
AnaXI-2	Deleting M1, inserting MAKEYFPQIQKIKFEGKDSKNPL, H60Q, A325S
NeoXI-1	Deleting MFESIKRKLFGIKTTN, inserting MAKE
NeoXI-2	Deleting MFESIKRKLFGIKTTN, inserting MAKE, Q93K, H346T, A436G
RhoXI-1	Deleting TNDPSFSKIVDHRYDSFNHSKGSDFENGKLKLSDLYESALQAGPISAASGKQERLENIINQNI, inserting LDESELPQMLRDRYASFDEGQGKAFEEGRLSLEDLVDYAKQNGEPRQISGKQELYETIVALYSK, N290D, D291A, D292G
BeaXI-1	D9P, A31K, E39K, R81D, A110D, V125Y, G151M, T164A, N175D, Q179K, Q204R, G210A, Y251I, Y350E, R382I, K385D, G389S, Q396K, K400D, T406E, D439R, N440
RhiXI-1	Deleting MS, inserting MTKEYFPEIGKIPFEGTESKTPMAFHYYEPERVVKGKKMK, deleting NTSIPFD
CalXI	K332N, Y370A, D403T, E438F

Table S5. Amino acid sequences of ancestral XIs constructed in this study.

XIs	Amino acid sequences
AncXI-1	MTKEYFPGIGKIKFEGKESKNPMAFHYYDAEKVVMGKKMKDWLKFAMAWWHTLCAEGADQFGG<br>GTKSFPWNEGADPVQIAKQKVDAGFEIMQKLGIEYYCFHDVDLVSEGNSIEEYESNLKAIVAYLKQK<br>QQETGIKLLWGTANVFGHKRYMNGAATNPDFDVVARAAVQIKNAIDATIELGGSNYVFWGGREGYM<br>SLLNTDQKREKEHLATMLTMARDYARAKGFKGTFLIEPKPMEPTKHQYDVDTETVIGFLKAHGLDK<br>DFKVNIEVNHATLAGHTFEHELACAVDNGMLGSIDANRGDYQNGWDTDQFPIDNFELTQAMMQIIR<br>NGGLGNGGTNFDAKTRRNSTDLEDIFIAHISGMDAMARALESAAALLEESPYKKMKAERYASFDSGI<br>GKDFEEGKLTLEQVYEYGKQNGEPKQTSGKQELYEAIVAMYC
AncXI-2	MKVTIGNKEYFPGIGKIKFEGRESKNPLAFKWYDENRVVAGKTMKEHLRFAIAYWHTFCGTGGDPFG<br>PGTKSFPWDEASDPIERAKDKMDAAFEFITKIGVPYYCFHDVDLVDEGDSIAEYEKRLQAIVDYAKQK<br>QKESGVKLLWGTANVFSHPRYMNGAATNPDFNVVAHAATQVKNAIDATIALGGENYVFWGGREGY<br>MSLLNTDMKREKEHLARFLTMARDYARKQGFKGTFLIEPKPMEPTKHQYDFDAETVIGFLRHYGLD<br>KDFKLNIEVNHATLAGHTFQHELQVAADAGMLGSIDANRGDYQNGWDTDQFPINLYELTEAMLVILQ<br>AGGFTSGGINFDAKIRRNSTDLEDLFIAHIAGMDAFARALIIADKILEKSPYKKLRKERYASFDSGKGK<br>EFEEGKLTLEDLRKIAESGGEPKQISGKQELYEQIINMYI
AncXI-3	MKEYFKNVPKIKYEGPKSKNPFAFKYYNPDEVILGKTMKEHLKFAMSYWHTLCAEGTDMFGVGTM<br>DKSFGAKTDPMELAKAKADAGFELMEKLGIDYFCFHDVDIAPEGKTLEESNANLDVIVDYIKELMKK<br>TGIKLLWGTANCFSNPRYMHGAGTSPNADVFAYAAAQIKKAIDATIKLGGKGYVFWGGREGYETLLN<br>TDMGLELDNMARLMKMAVEYGRSIGFKGDFYIEPKPKEPTKHQYDFDVATVIGFLRKYGLEKDFKM<br>NIEANHATLAGHTFQHELRTARINGVFGSIDANQGDLLLGWDTDQFPTNVYDATLCMYEVIKAGGFT<br>NGGLNFDAKARRGSFTLEDIALAYIAGMDTFALGLRKAAKIIEDGRIDEFVENRYSSYNTGIGKDIVDG<br>KVTLEELEEYALKLGETNVESGRQEYLESIVNNILFSG
AncXI-4	MEYFPNVPKIKYEGPDSKNPLAFKYYNPDEVIGGKTMKEHLRFSVAYWHTFTADGSDPFGVGAPTM<br>QRPWDKTFSDPMDLAKARVEAAFEFMEKLGVPYFCFHDRDIAPEGETLRETNKNLDEIVDLIKELMK<br>ESGIKLLWGTANLFSHPRYVHGAATSCNADVFAYAAAQVKKALEITKELGGENYVFWGGREGYETLL<br>NTDMKLELDNLARFLHMAVDYAKEIGFKGQFLIEPKPKEPTKHQYDFDVATVLAFLRKYGLDKHFKL<br>NIEANHATLAGHTFQHELRVARINGMLGSIDANQGDLLLGWDTDQFPTNIYDTTLAMYEVLKNGGL<br>APGGLNFDAKVRRGSFEPEDLFYAHIAGMDAFAKGLKVAHKLIEDGVLEKFIEERYSSYYKSGIGKDI<br>VEGKVNFKELEEYALNHGNEEIKNKSGRQEMLESILNQYIFSALA

Table S6. XIs in NCBI database showing the highest similarity to ancestral XIs constructed in this study.

**Ancestral XIs**	**Proteins showing highest similarity to ancestral XIs**	**Strains**	**Per. Ident**
AncXI-1	OUM58912.1	*Piromyces* sp. E2	90.14%
AncXI-2	WP_028668460.1	*Runella zeae*	83.03%
AncXI-3	WP_092638076.1	*Acetanaerobacterium elongatum*	82.15%
AncXI-4	WP_094546057.1	*Petroclostridium xylanilyticum*	82.31%

Table S7. Fermentation performances of *S. cerevisiae* CRD5HS, CRD5BJ, CRD5AE, CRD5SC and CRD51 in YPX40 and YPD80X40 medium.

Medium	Strains	Production (g/L)				Yield (g/g consumed sugars)				Specific xylose consumption rate (g/g DCW/h)[a]	Specific ethanol production rate (g/g DCW/h)[b]	μ_{max} (h^{-1})
		Biomass	Xylitol	Glycerol	Ethanol	Biomass	Xylitol	Glycerol	Ethanol			
YPX40	CRD5AE	7.26±0.03	0.32±0.01	1.02±0.06	17.94±0.11	0.170±0.001	0.007±0.000	0.024±0.001	0.420±0.003	0.698±0.001	0.293±0.002	0.186±0.000
	CRD5BJ	6.61±0.14	0.35±0.01	0.70±0.02	17.60±0.25	0.162±0.003	0.009±0.000	0.017±0.000	0.432±0.006	0.832±0.001	0.359±0.005	0.199±0.002
	CRD5HS	7.74±0.21	0.34±0.02	0.74±0.01	17.86±0.12	0.189±0.005	0.008±0.000	0.018±0.000	0.437±0.003	0.851±0.001	0.372±0.002	0.203±0.001
	CRD5SC	5.87±0.18	0.28±0.00	0.74±0.02	17.70±0.08	0.145±0.005	0.007±0.000	0.018±0.001	0.436±0.002	0.808±0.003	0.352±0.002	0.181±0.000
	CRD51	6.95±0.28	0.99±0.03	0.51±0.01	17.37±0.16	0.160±0.007	0.023±0.001	0.012±0.000	0.401±0.004	0.631±0.002	0.253±0.002	0.152±0.000
YPD80X40	CRD5AE	9.84±0.15	0.75±0.06	4.06±0.01	51.19±0.46	0.083±0.001	0.023±0.000	0.034±0.000	0.430±0.004	0.172±0.001	0.071±0.003	0.249±0.001
	CRD5BJ	8.01±0.23	0.41±0.00	4.07±0.03	55.00±0.48	0.065±0.002	0.010±0.000	0.033±0.000	0.447±0.004	0.594±0.011	0.411±0.006	0.232±0.001
	CRD5HS	8.25±0.31	0.38±0.00	4.22±0.01	55.84±0.05	0.068±0.003	0.009±0.000	0.035±0.000	0.457±0.000	0.579±0.003	0.452±0.011	0.234±0.002
	CRD5SC	6.95±0.38	0.51±0.03	2.93±0.05	44.44±0.11	0.068±0.004	0.018±0.001	0.029±0.000	0.435±0.001	0.243±0.010	0.138±0.005	0.222±0.000
	CRD51	9.74±0.43	0.64±0.06	4.24±0.00	54.89±1.22	0.078±0.003	0.016±0.000	0.034±0.000	0.437±0.010	0.222±0.002	0.093±0.012	0.248±0.003

[a] The specific xylose consumption rate was calculated during xylose consumption phase in YPD80X40 medium;

[b] The specific ethanol production rate was calculated during xylose consumption phase in YPD80X40 medium, where ethanol is produced mostly from xylose and to a lesser extent from glucose.

Table S8. Fermentation performances of *S. cerevisiae* CRD5HS, CRD5BJ, CRD5AE, CRD5SC in lignocellulosic hydrolysates at different solid loadings[a].

Substrate	Solid loading (wt%)	Strain	Fermentation strategy	Initial OD_{600}	Residual sugars (g/L)		Production (g/L)			Ethanol yield[c]
					Glucose	Xylose	Glycerol	Acetic acid[b]	Ethanol	
DLCA(ch) Corn stover	30	CRD5HS	SHCF	2	0.39±0.46	4.29±3.76	6.10±0.43	7.71±0.89	73.06±1.43	0.71±0.02
		CRD5BJ			0.00±0.00	8.04±0.37	5.49±0.03	7.86±0.22	70.38±0.71	0.71±0.00
		CRD5AE			0.00±0.00	13.71±0.57	5.22±0.69	8.02±1.87	66.16±0.54	0.67±0.00
		CRD5SC			0.00±0.00	24.19±0.19	5.26±0.43	8.14±0.29	63.00±1.95	0.64±0.02
	35	CRD5HS	SSCF		2.88±0.41	24.23±0.91	7.96±0.07	9.88±0.03	73.41±0.20	0.62±0.02
		CRD5BJ			2.53±0.13	25.14±0.04	7.54±0.07	9.53±0.07	73.27±0.04	0.60±0.03
		CRD5AE			1.16±0.44	31.95±0.21	7.13±0.20	9.82±0.12	72.20±0.11	0.59±0.02
		CRD5SC			1.40±0.39	39.22±0.66	7.04±0.02	9.73±0.26	67.70±0.43	0.58±0.00
		CRD5HS	Fed-batch[d]		1.03±0.17	7.17±1.63	6.14±0.06	9.28±0.34	85.95±0.04	0.70±0.01
			Fed-batch[e]		0.69±0.09	7.34±0.92	5.62±0.27	8.23±0.54	83.34±3.75	0.70±0.03
	37		Fed-batch[f]		3.26±0.11	8.32±0.65	6.81±0.07	9.37±0.09	85.47±0.55	0.68±0.00
			Fed-batch[g]		2.47±0.37	10.97±0.66	5.65±0.22	8.92±0.48	84.63±3.17	0.66±0.02
DLCA(sa) Corn cob	25	CRD5HS	SHCF		0.00±0.00	4.89±0.55	5.91±0.02	2.85±0.12	72.57±1.72	0.84±0.01
		CRD5BJ			0.00±0.00	6.43±0.64	5.62±0.24	2.74±0.10	72.43±0.51	0.84±0.01
		CRD5AE			0.00±0.00	24.46±9.15	4.76±0.80	4.65±2.62	60.90±8.68	0.72±0.12
		CRD5SC			0.00±0.00	38.05±4.37	5.06±0.02	2.85±0.47	57.73±0.40	0.69±0.01
	30	CRD5HS			1.30±0.06	24.70±4.59	7.78±0.17	3.50±0.22	76.78±3.17	0.77±0.02
		CRD5BJ			0.46±0.26	38.01±2.41	7.09±0.31	3.50±0.16	69.38±0.97	0.70±0.01
		CRD5AE			0.04±0.03	48.68±5.93	6.97±0.50	4.06±0.78	64.09±3.40	0.68±0.04
		CRD5SC			0.15±0.19	67.38±6.91	6.57±0.19	4.04±0.58	55.22±3.09	0.58±0.07
	35	CRD5HS	Fed-batch[h]	20	5.32±0.87	9.20±0.26	9.76±0.19	5.00±0.06	89.94±0.29	0.69±0.01
	37		Fed-batch[i]		10.42±3.98	14.25±2.42	10.42±0.41	5.44±0.00	90.87±1.44	0.62±0.02
	40		Fed-batch[j]		15.58±5.93	54.02±2.39	7.85±0.10	6.27±0.03	84.37±0.6	0.52±0.00
			Fed-batch[k]		17.38±1.83	29.80±0.36	8.92±0.41	6.14±0.09	94.76±0.98	0.57±0.01

[a] The lignocellulosic hydrolysates were prepared by pretreating corn stover or corn cob by DLCA pretreatment method with $Ca(OH)_2$ or H_2SO_4 as the pretreatment reagent.
[b] Most of the acetic acid are generated during the pretreatment processes.
[c] Ethanol yield was calculated based on the theoretical ethanol production from total glucan and xylan in lignocellulosic feedstock with the formula: Ethanol yield = Produced ethanol (g)/theoretical ethanol production from total glucan and xylan (g).
[d, e, f, g, h, i, j, k] Detailed fed-batch modes were shown in Fig 9E, F, G, H and Fig S15E, F, G, H.

Table S9. SNPs detected in evolved mutants.

Gene symbol	Gene description	CRD5AE	CRD5HS	CRD5BJ	CRD5SC
MST28	DUP240 family protein MST28	57T>G(N19K)<br>67C>T(R23C)<br>80C>A(T27N)	57T>G(N19K)<br>67C>T(R23C)<br>80C>A(T27N)<br>91G>C(G31R)<br>97T>C(Y33H)<br>101A>G(N34S)	67C>T(R23C)	57T>G(N19K)
ATG13	serine/threonine protein kinase regulatory subunit ATG13			965A>T(Q322L)	
COS2	Cos2p	100G>A(G34S)<br>121C>T(H41Y)<br>399A>C(L133F)			
COS4	Cos4p	49G>T(V17F)			
GEA2	Arf family guanine nucleotide exchange factor GEA2			1881T>G(Y627X)	
AVL9	Avl9p	1854A>T(E618D)<br>1855C>G(H619D)			
CTR1	high-affinity Cu transporter CTR1			220A>G(S74G)	
FRE7	putative ferric-chelate reductase	1499C>G(S500W)			
FRE8	putative ferric-chelate reductase			1319T>C(L440P)	
ISU1	iron-binding protein ISU1		175G>T(V59F)	301G>A(A101T)	196G>C(A66P)
ATM1	ATP-binding cassette Fe/S cluster precursor transporter ATM1	996C>G(N332K)			
YOR389W	uncharacterized protein	83G>A(G28E)			
YPL277C	uncharacterized protein			457G>A(D153N)<br>697T>C(F233L)	457G>A(D153N)
NUM1	Num1p	3621C>A(D1207E)<br>3701A>G(N1234S)<br>5710G>C(V1904L)	1879G>A(V627I)<br>3621C>A(D1207E)<br>3701A>G(N1234S)<br>5252C>T(S1751F)	3701A>G(N1234S)<br>4561C>A(H1521N)<br>5710G>C(V1904L)	3621C>A(D1207E)
ATP6	F1F0 ATP synthase subunit a			320T>A(M107K)	
MSH1	mismatch repair ATPase MSH1			2354C>T(A785V)	
UBP11	ubiquitin-specific protease UBP11		1909G>A(G637R)		
PIR1	beta-1,3-glucan linked protein		488C>A(T163N)	488C>A(T163N)	488C>A(T163N)
MIX23	Mix23p	209C>T(S70F)			
AAD3	putative aryl-alcohol dehydrogenase			749A>G(K250R)	
AAD4	putative aryl-alcohol dehydrogenase	861T>A(N287K)	683A>G(K228R)<br>704G>A(R235K)<br>721A>C(I241L)<br>723T>G(I241M)<br>727T>A(S243T)<br>747A>T(E249D)	704G>A(R235K)	683A>G(K228R)<br>704G>A(R235K)<br>721A>C(I241L)<br>861T>A(N287K)
AAD10	putative aryl-alcohol dehydrogenase		448A>C(T150P)<br>449C>A(T150K)	448A>C(T150P)<br>449C>A(T150K)	
AAD14	putative aryl-alcohol dehydrogenase	448A>G(I150V)	448A>G(I150V)	448A>G(I150V)	
DDR48	DNA damage-responsive protein 48		629G>A(S210N)<br>634A>G(N212D)	629G>A(S210N)	
YMR045C	gag-pol fusion protein	25C>A(H9N)<br>34A>G(I12V)<br>71A>G(K24R)<br>94C>A(P32T)	71A>G(K24R)		25C>A(H9N) 34A>G(I12V)<br>71A>G(K24R) 86C>A(T29N)<br>94C>A(P32T)
YDR034C-D	gag-pol fusion protein	3192C>A(D1064E)	3192C>A(D1064E)<br>3416T>C(I1139T)		
YDR365W-B	gag-pol fusion protein	602A>G(Y201C)	602A>G(Y201C)		
YMR050C	gag-pol fusion protein	2682A>C(Q894H)	2682A>C(Q894H)		2682A>C(Q894H)
YBL005W-B	gag-pol fusion protein		190C>T(P64S)	373G>A(G125R)<br>376G>C(A126P)<br>400C>T(P134S)<br>404A>C(Q135P)<br>407A>C(Y136S)	502C>T(H168Y)<br>506T>C(V169A)
YGR027W-B	gag-pol fusion protein			1217T>C(F406S)<br>4257C>G(D1419E)	
YGR161C-D	gag-pol fusion protein			2700A>T(Q900H)	2769A>C(Q923H)
YLR410W-B	gag-pol fusion protein			1390C>T(P464S)	
YNL284C-B	gag-pol fusion protein		1363T>C(S455P)	908C>A(P303H)	1363T>C(S455P)
YGR109W-B	gag-pol fusion protein			2752G>A(G918S)	
YOR142W-B	gag-pol fusion protein			3050A>G(N1017S)	
YER160C	gag-pol fusion protein		2625A>C(E875D)	2625A>C(E875D)<br>3416G>A(R1139H)	
YGR038C-B	gag-pol fusion protein			200C>T(P67L)	40C>G(H14D)
YDR316W-B	gag-pol fusion protein		32A>C(H11P)	32A>C(H11P)	

YML133C	Y' element ATP-dependent helicase		4552C>A(L1518I)		4552C>A(L1518I)
MTR4	ATP-dependent RNA helicase MTR4			1242C>A(F414L)	
YLL066C	Y' element ATP-dependent helicase		1180C>T(P394S)	1180C>T(P394S)	1318T>G(C440G) 1417A>G(T473A)<br>1418C>A(T473N) 1449G>T(E483D)<br>1450A>G(N484D) 1460A>G(E487G)<br>1524T>G(F508L)<br>1612A>G(T538A)
YRF1-1	Y' element ATP-dependent helicase protein 1 copy 1				4777T>G(F1593V)
YRF1-2	Y' element ATP-dependent helicase protein 1 copy 2			761T>C(F254S)	
YRF1-4	Y' element ATP-dependent helicase protein 1 copy 4		269T>A(I90K)<br>2056G>A(E686K)<br>3192T>A(N1064K)	269T>A(I90K)<br>4909G>C(V1637L)	3350T>C(I1117T)<br>3626A>G(N1209S)
YRF1-5	Y' element ATP-dependent helicase protein 1 copy 5		2843C>T(T948I)	2734G>A(A912T)	2957A>G(K986R)
YRF1-8	Y' element ATP-dependent helicase protein 1 copy 8			563C>T(A188V)	563C>T(A188V)
ASG1	Asg1p		2633G>A(S878N)<br>2636G>A(S879N)		
BUD5	Ras family guanine nucleotide exchange factor BUD5			511C>A(L171I)<br>532A>G(N178D)	407G>A(R136Q)<br>511C>A(L171I) 532A>G(N178D)
MTF2	Mtf2p				833A>C(K278T)
FLO1	flocculin FLO1	509A>G(N170S)<br>511G>A(V171I)<br>559G>A(D187N)<br>572G>A(S191N)<br>1631G>A(S544N)<br>1636A>G(I546V)<br>3077A>G(N1026S)	511G>A(V171I)<br>559G>A(D187N)<br>572G>A(S191N)<br>1631G>A(S544N)<br>3077A>G(N1026S)	572G>A(S191N)<br>925A>G(I309V)	559G>A(D187N) 857C>T(T286I)<br>1631G>A(S544N)<br>1636A>G(I546V)
FLO5	flocculin FLO5	1259T>C(F420S)<br>1265C>T(S422L)<br>4037C>T(T1346I)<br>4039G>A(A1347T)	1259T>C(F420S)<br>1265C>T(S422L)<br>4181C>T(T1394I)<br>4183G>A(A1395T)		1259T>C(F420S) 4181C>T(T1394I)<br>4183G>A(A1395T)
FLO9[a]	flocculin FLO9	455C>G(T152S)<br>469A>G(I157V)<br>977C>T(T326I)<br>979G>A(A327T)		455C>G(T152S)<br>458C>G(T153S)<br>469A>G(I157V)	
FLO9[a]	flocculin FLO9	1892C>G(T631S)		1892C>G(T631S)	907T>G(L303V)<br>1892C>G(T631S)
FLO10	Flo10p	2017A>T(T673S)<br>2018C>T(T673I)<br>2021C>G(S674C)			2018C>T(T673I)
VVS1	Vvs1p		1060G>C(D354H)		
TIR4	Tir4p	440C>A(A147D)			440C>A(A147D)
HPF1	mannoprotein	2290G>A(V764I)<br>2302C>T(H768Y)<br>2404G>C(V802L)	1781T>A(F594Y)<br>2404G>C(V802L)	1781T>A(F594Y)<br>1828T>A(Y610N)<br>2290G>A(V764I)<br>2302C>T(H768Y)<br>2404G>C(V802L)	
BBC1	Bbc1p	2411C>T(A804V)	2411C>T(A804V)<br>2413C>T(P805S)		
SPA2	Spa2p	2903A>C(K968T)	2903A>C(K968T)<br>2906A>G(E969G)		2903A>C(K968T) 2906A>G(E969G)
PRM7	pheromone-regulated protein PRM7			1078G>A(V360I)	
FAR11	Far11p			2806C>A(R936S)	
NCR1	sphingolipid transporter				1350G>C(E450D)
PRY2	sterol-binding protein				221T>A(V74D)
ETR1	enoyl-[acyl-carrier-protein] reductase		286G>C(G96R)		
FEX2	fluoride transporter			449C>T(T150I)	449C>T(T150I)
CPS1	Gly-Xaa carboxypeptidase	1124A>G(K375R)			
URA3	orotidine-5'-phosphate decarboxylase	478G>T(A160S)		478G>T(A160S)	
SUC2	beta-fructofuranosidase SUC2			156T>A(D52E)	
HXT6	hexose transporter HXT6				1666G>A(A556T)
SEC59	dolichol kinase				1141C>T(H381Y)
PUF4	Puf4p	2147C>T(A716V)			
FRS2	phenylalanine--tRNA ligase subunit alpha			304G>T(G102C)	
DPH2	2-(3-amino-3-carboxypropyl)histidine synthase			1556G>A(R519H)	
RPR2	ribonuclease P protein subunit RPR2			483T>A(D161E)	

ARO8	bifunctional 2-aminoadipate transaminase/aromatic-amino-acid:2-oxoglutarate transaminase			302G>A(R101K)	
ATP23	putative metalloprotease		116C>G(A39G)		
YER189W	uncharacterized protein		160C>G(L54V)		
XRN1	chromatin-binding exonuclease XRN1				2552G>T(R851M)
PEX1	AAA family ATPase peroxin 1	8310T>G(D2770E)<br>8358A>T(E2786D)		8310T>G(D2770E)<br>8358A>T(E2786D)	8310T>G(D2770E) 8358A>T(E2786D)
PEX6	AAA family ATPase peroxin 6		2103C>A(Y701X)		
AXL1	Axl1p		1888G>T(E630X)		
ENA2	Na(+)-exporting P-type ATPase ENA2	580G>C(E194Q)			
THI5	4-amino-5-hydroxymethyl-2-methylpyrimidine phosphate synthase		111G>T(M37I)	111G>T(M37I)	111G>T(M37I)
PHO12	acid phosphatase PHO12		1508A>C(E503A)<br>1509A>T(E503D)<br>1535G>A(G512D)	476T>G(V159G)<br>1508A>C(E503A)<br>1509A>T(E503D)<br>1535G>A(G512D)<br>2535A>C(E845D)	476T>G(V159G) 518T>G(V173G)<br>1508A>C(E503A) 1509A>T(E503D)
SSP2	Ssp2p			1011G>A(M337I)	
CRZ1	DNA-binding transcription factor CRZ1				403C>A(Q135K)
FLC3	putative flavin adenine dinucleotide transporter	151T>A(F51I)			
CSS1	Css1p		957A>T(E319D)		957A>T(E319D) 983G>A(G328D)
CDC25	Ras family guanine nucleotide exchange factor CDC25				2092C>T(L698F)
TRA1	histone acetyltransferase TRA1			9698T>A(I3233N)	
DDI3	cyanamide hydratase			634G>A(D212N)	
PAU15	seripauperin PAU15	55G>A(A19T)		55G>A(A19T)<br>185G>C(S62T)<br>186T>A(S62R)	185G>C(S62T)<br>186T>A(S62R)
BUD27	Bud27p		1511T>C(I504T)<br>1619T>C(V540A)<br>1621A>G(I541V)	1619T>C(V540A)<br>1621A>G(I541V)<br>1631T>C(I544T)	
FLC2	flavin adenine dinucleotide transporter FLC2			493G>A(A165T)	
YPL277C	uncharacterized protein			457G>A(D153N)<br>697T>C(F233L)	457G>A(D153N)
VHS3	phosphopantothenoylcysteine decarboxylase complex subunit VHS3	1932A>C(E644D)<br>1938A>C(E646D)	1923A>T(E641D)<br>1932A>C(E644D)<br>1938A>C(E646D)		
CYC8	transcription regulator CYC8	1545T>A(H515Q)			
FIG2	Fig2p	1408G>A(A470T)<br>1886C>T(P629L)<br>3365G>A(S1122N)			1430C>T(T477M) 1886C>T(P629L)<br>3365G>A(S1122N)
MTL1	Mtl1p		270T>G(D90E)<br>273G>T(E91D)<br>291G>T(E97D)	261T>G(D87E)<br>270T>G(D90E)<br>273G>T(E91D)	270T>G(D90E)<br>273G>T(E91D)

[a] These two genes are located at different locations of the chromosome, but both are most similar to *FLO9*, indicating that *S. cerevisiae* ATCC26603 has undiscovered flocculation genes.

Table S10. Indels detected in evolved mutants.

Gene symbol	Gene description	CRD5AE	CRD5HS	CRD5BJ	CRD5SC
SEC7	Arf family guanine nucleotide exchange factor SEC7	317_318insTGAAGATGAAGATGAAGATGAAGATGAAGA(D106delinsDEDEDEDEDED)	317_318insTGAAGATGAAGATGAAGATGAAGATGAAGA(D106delinsDEDEDEDEDED)		317_318insTGAAGATGAAGATGAAGATGAAGATGAAGA(D106delinsDEDEDEDEDED)
NUM1	Num1p		5284_5285insGG(K1762fs)		
DDR48	DNA damage-responsive protein 48	1283_1284insTTC(G428delinsGS) 1307_1308insATC(G436delinsGS)			
YOR192C-B	gag-pol fusion protein		687_698del(229_233del)	687_698del(229_233del)	687_698del(229_233del)
AIP5	Aip5p				1399_1400insAAGAAGAAGAAGAAGAAGAAG(K467delinsKEEEEEEE)
SCW11	putative glucan endo-1,3-beta-D-glucosidase		736_747del(246_249del)		655_656insCTACGTCGTCCT(P219delinsPTSSS)
VBA5	basic amino acid transporter		1480delT(S494fs)	1480delT(S494fs)	
SNZ1	pyridoxine biosynthesis protein SNZ1		632_657del(211_219del)	632_657del(211_219del)	632_657del(211_219del)
VHS3	phosphopantothenoylcysteine decarboxylase complex subunit VHS3			1915_1941del(639_647del)	
PSR1	phosphatase		471_472insCAA(Q157delinsQQ)		
YRF1-1	Y' element ATP-dependent helicase protein 1 copy 1		445_467del(149_156del) 524_537del(175_179del)		445_467del(149_156del) 524_537del(175_179del)
ASG1	Asg1p	2612_2613insTAATAATAATAATAATAATAATAATAA(N871delinsNNNNNNNNNN)			
EHT1	medium-chain fatty acid ethyl ester synthase/esterase		965_966del(322_322del)		
CDC27	anaphase promoting complex subunit CDC27		1138_1139insATAATAATAATAATAATAATA(I380delinsNNNNNNNI)		
CYC8	transcription regulator CYC8				1590_1595del(530_532del)
FIG2	Fig2p	4375_4376insGTGCGGGTAAAAATTGTGAACAAACTTCCC(S1459delinsCAGKNCEQTSP)			4375_4376insGTGCGGGTAAAAATTGTGAACAAACTTCCC(S1459delinsCAGKNCEQTSP)
MTL1	Mtl1p	202_207del(68_69del)	202_207del(68_69del)		

867 putative XIs and 16 verified XIs active in *S. cerevisiae* selected to to build the phylogenetic tree in this study

Source	GenBank	Superking	Phylum	Length	Notes
Planctomycetes bacterium	HIE71014.1	Bacteria	Planctomycetes	406	
Acidobacteriaceae bacterium	MCA2963563.1	Bacteria	Acidobacteria	413	
Solibacteraceae bacterium	MBE7544281.1	Bacteria	Acidobacteria	412	
Acidobacteria bacterium	HHR99801.1	Bacteria	Acidobacteria	424	
Acidobacteria bacterium	HDU62502.1	Bacteria	Acidobacteria	465	
Acidobacteriaceae bacterium	MBV8831390.1	Bacteria	Acidobacteria	412	
Bryobacterales bacterium	HCC56763.1	Bacteria	Acidobacteria	440	
Acidobacteria bacterium	MBI1354310.1	Bacteria	Acidobacteria	440	
Bryobacterales bacterium	MCC6537863.1	Bacteria	Acidobacteria	419	
Bryobacteraceae bacterium	MBC7927640.1	Bacteria	Acidobacteria	413	
Streptomyces rochei	P22857.2	Bacteria	Actinobacteria	394	
Mycolicibacterium parafortuitum	BBY75161.1	Bacteria	Actinobacteria	394	
Xylanimicrobium sp. FW10M-9	WP_165350385.1	Bacteria	Actinobacteria	390	
Nocardioides sp. Kera G14	WP_227783188.1	Bacteria	Actinobacteria	386	
Nakamurella silvestris	WP_119384398.1	Bacteria	Actinobacteria	391	
Crossiella cryophila	WP_221490571.1	Bacteria	Actinobacteria	403	
Actinoalloteichus	WP_075742763.1	Bacteria	Actinobacteria	396	
Halosaccharopolyspora lacisalsi	MBA8823739.1	Bacteria	Actinobacteria	413	
Nocardiopsis chromatogenes	WP_017625353.1	Bacteria	Actinobacteria	387	
Thermobifida fusca	WP_011292029.1	Bacteria	Actinobacteria	385	
Nocardioides terrigena	WP_107767187.1	Bacteria	Actinobacteria	390	
Kribbella sp. ALI-6-A	WP_077014501.1	Bacteria	Actinobacteria	392	
Nocardioides sp. zg-536	WP_205291011.1	Bacteria	Actinobacteria	395	
Streptomyces nanshensis	WP_079132804.1	Bacteria	Actinobacteria	432	
Knoellia locipacati	GEQ13698.1	Bacteria	Actinobacteria	404	
Dactylosporangium vinaceum	WP_223097782.1	Bacteria	Actinobacteria	393	
Actinomycetia bacterium	MPZ70200.1	Bacteria	Actinobacteria	390	
Actinomycetia bacterium	MBV9204755.1	Bacteria	Actinobacteria	433	
Quadrisphaera granulorum	WP_109773293.1	Bacteria	Actinobacteria	405	
unclassified Ornithinimicrobium	WP_131104542.1	Bacteria	Actinobacteria	437	
Motilibacter rhizosphaerae	WP_130493678.1	Bacteria	Actinobacteria	395	
XylA Arthrobacter sp. Rue61a	AFR30717.1	Bacteria	Actinobacteria	410	
Brooklawnia cerclae	WP_167171764.1	Bacteria	Actinobacteria	396	
Ruaniaceae bacterium KH17	SNU01967.1	Bacteria	Actinobacteria	396	
Actinomyces sp. oral taxon 448 str. F0400	EGQ74558.1	Bacteria	Actinobacteria	459	
Actinomyces sp. oral taxon 180 str. F0310	EFU60787.1	Bacteria	Actinobacteria	431	
Cellulomonas aerilata	WP_146905260.1	Bacteria	Actinobacteria	395	
Kocuria flava	WP_101851423.1	Bacteria	Actinobacteria	413	
Nesterenkonia massiliensis	WP_044495285.1	Bacteria	Actinobacteria	393	
Cellulosimicrobium terreum	MBD5785448.1	Bacteria	Actinobacteria	414	
Nakamurella sp. s14-144	WP_124800035.1	Bacteria	Actinobacteria	395	
Ornithinicoccus halotolerans	WP_153396119.1	Bacteria	Actinobacteria	392	
Frankia elaeagni	WP_018636518.1	Bacteria	Actinobacteria	414	
Actinomycetia bacterium	NBU22838.1	Bacteria	Actinobacteria	390	
Actinomycetia bacterium	MSV56193.1	Bacteria	Actinobacteria	396	
Demequina lutea	NYI40830.1	Bacteria	Actinobacteria	405	
Arthrobacter globiformis NBRC 12137	GAB13648.1	Bacteria	Actinobacteria	417	
Propionibacteriaceae bacterium	MCB0910186.1	Bacteria	Actinobacteria	415	
Micromonospora sp. ATCC 39149	EEP71362.1	Bacteria	Actinobacteria	416	
Actinomycetia bacterium	NBY38603.1	Bacteria	Actinobacteria	390	
Motilibacter aurantiacus	WP_196791195.1	Bacteria	Actinobacteria	417	
Prauserella rugosa	WP_030530087.1	Bacteria	Actinobacteria	384	
Actinomycetia bacterium	TXH30757.1	Bacteria	Actinobacteria	390	
Actinomycetales bacterium	MBU6244587.1	Bacteria	Actinobacteria	393	
Candidatus Nanopelagicales bacterium	MBL6834779.1	Bacteria	Actinobacteria	389	
Acidimicrobiales bacterium	MCB0960879.1	Bacteria	Actinobacteria	378	
Frankia inefficax	WP_013428114.1	Bacteria	Actinobacteria	411	
Actinomycetia bacterium	MSV42525.1	Bacteria	Actinobacteria	404	
Planomonospora parontospora subsp. Antibioti	GGL55919.1	Bacteria	Actinobacteria	423	
Saccharothrix sp. NRRL B-16348	KOX31051.1	Bacteria	Actinobacteria	393	
Geodermatophilus daqingensis	WP_218859424.1	Bacteria	Actinobacteria	412	
Amycolatopsis arida	WP_092535096.1	Bacteria	Actinobacteria	411	
Nocardia crassostreae	WP_067536528.1	Bacteria	Actinobacteria	393	
Nocardia arthritidis	QIS12133.1	Bacteria	Actinobacteria	422	
Candidatus Dormibacteraeota bacterium	MBO0701999.1	Bacteria	Candidatus Dormib	397	

Tetrasphaera sp. F2B08	WP_152177653.1	Bacteria	Actinobacteria	396	
Acidiferrimicrobium australe	MST34992.1	Bacteria	Actinobacteria	401	
Chloroflexi bacterium	MBV8716718.1	Bacteria	Chloroflexi	389	
Chloroflexi bacterium	TMD60731.1	Bacteria	Chloroflexi	390	
Chloroflexi bacterium	MBA3959071.1	Bacteria	Chloroflexi	389	
Phycisphaerae bacterium	MAY75944.1	Bacteria	Planctomycetes	384	
Phycisphaeraceae bacterium	QOJ19100.1	Bacteria	Planctomycetes	394	
Phycisphaerae bacterium	MBL9032868.1	Bacteria	Planctomycetes	394	
Phycisphaerales bacterium	TVQ61620.1	Bacteria	Planctomycetes	390	
Pyrinomonadaceae bacterium	MBC7773765.1	Bacteria	Planctomycetes	390	
Luteolibacter ambystomatis	WP_211632431.1	Bacteria	Verrucomicrobia	387	
Humisphaera borealis	WP_206294631.1	Bacteria	Planctomycetes	394	
Planctomycetaceae bacterium	MAE61518.1	Bacteria	Planctomycetes	391	
Poriferisphaera corsica	WP_145079312.1	Bacteria	Planctomycetes	391	
Phycisphaerales bacterium	HCD32847.1	Bacteria	Planctomycetes	392	
Phycisphaeraceae bacterium	MCC7193734.1	Bacteria	Planctomycetes	392	
Phycisphaeraceae bacterium	MCC5830486.1	Bacteria	Planctomycetes	391	
Thermus thermophilus	WP_014677651.1	Bacteria	Deinococcus-Therı	387	
Thermus thermophilus	BAA14301.1	Bacteria	Deinococcus-Therı	387	Active XI in S. cerevisiae
Trueperaceae bacterium	MBW6456940.1	Bacteria	Deinococcus-Therı	387	
Deinococcus hopiensis	WP_084045522.1	Bacteria	Deinococcus-Therı	404	
Trueperaceae bacterium	MBA2666576.1	Bacteria	Deinococcus-Therı	389	
Acidimicrobiia bacterium	MBK5287586.1	Bacteria	Actinobacteria	386	
Actinomycetia bacterium	MBI2703814.1	Bacteria	Actinobacteria	391	
Solirubrobacter sp. CPCC 204708	WP_202953678.1	Bacteria	Actinobacteria	403	
Solirubrobacterales bacterium	MBV9197439.1	Bacteria	Actinobacteria	395	
Actinomycetia bacterium	NOY54604.1	Bacteria	Actinobacteria	387	
Thermoleophilia bacterium	MCC6830352.1	Bacteria	Actinobacteria	389	
Actinomycetia bacterium	MSV50860.1	Bacteria	Actinobacteria	387	
Streptosporangiales bacterium	MBO0829911.1	Bacteria	Actinobacteria	390	
Sulfobacillus thermosulfidooxidans	PSR35789.1	Bacteria	Firmicutes	377	
Sulfobacillus acidophilus	PSR22433.1	Bacteria	Firmicutes	381	
Calditrichaeota bacterium	RMD91835.1	Bacteria	Calditrichaeota	394	
Planctomycetes bacterium	MBI3855466.1	Bacteria	Planctomycetes	383	
Actinomycetia bacterium	MCB0918800.1	Bacteria	Actinobacteria	391	
Ktedonobacteria bacterium brp13	BCL81093.1	Bacteria	Chloroflexi	404	
bacterium	MBG97634.1	Bacteria	Unclassified Bacter	392	
Planctomycetes bacterium	TAH37413.1	Bacteria	Planctomycetes	415	
Planctomycetes bacterium	MBO47200.1	Bacteria	Planctomycetes	396	
Planctomycetes bacterium	HGY92650.1	Bacteria	Planctomycetes	392	
Planctomycetes bacterium	MSR74919.1	Bacteria	Planctomycetes	392	
bacterium TMED181	OUW45276.1	Bacteria	Unclassified Bacter	391	Selected XIs to express
Planctomycetes bacterium	MCA8968941.1	Bacteria	Planctomycetes	410	
Planctomycetes bacterium	MAD35031.1	Bacteria	Planctomycetes	392	
Planctomycetes bacterium	HIG10056.1	Bacteria	Planctomycetes	391	
Planctomycetes bacterium	MAF64343.1	Bacteria	Planctomycetes	392	
Planctomycetes bacterium	NOT29537.1	Bacteria	Planctomycetes	405	
Planctomycetes bacterium	MAB79125.1	Bacteria	Planctomycetes	396	
Planctomycetes bacterium	MBE7463363.1	Bacteria	Planctomycetes	390	
bacterium	RYX83595.1	Bacteria	Unclassified Bacter	386	
Abitibacteriaceae bacterium	MBV9868484.1	Bacteria	Abditibacteriota	386	
Armatimonadetes bacterium	MBV9848386.1	Bacteria	Armatimonadetes	389	
Chloroflexi bacterium	MCA1595413.1	Bacteria	Chloroflexi	390	
Akkermansiaceae bacterium	MBC7809200.1	Bacteria	Armatimonadetes	390	
Chloroflexi bacterium	MCC6566355.1	Bacteria	Chloroflexi	389	
Pleurocapsa minor GSE-CHR-MK 17-07R	MBW4435824.1	Bacteria	Cyanobacteria	388	
Bacteroidetes bacterium QS 8 68 28	PSR01615.1	Bacteria	Bacteroidetes	389	
Candidatus Brocadiae bacterium	NUN51074.1	Bacteria	Planctomycetes	389	
Caldilineaceae bacterium SB0666 bin 21	MXZ40983.1	Bacteria	Chloroflexi	390	
Caldilineaceae bacterium	MCB0087338.1	Bacteria	Chloroflexi	388	
Anaerolineae bacterium	MCA9894335.1	Bacteria	Chloroflexi	388	
Anaerolineae bacterium	MBX3084946.1	Bacteria	Chloroflexi	390	
Planctomycetes bacterium	MCC6672406.1	Bacteria	Planctomycetes	387	
Bacteroidetes bacterium SB0668 bin 1	MXW65336.1	Bacteria	Bacteroidetes	389	
Dehalococcoidia bacterium	MAG35523.1	Bacteria	Chloroflexi	391	
Ardenticatena maritima	GAP62313.1	Bacteria	Chloroflexi	398	
Acidobacteria bacterium	NOT49457.1	Bacteria	Acidobacteria	388	
Pyrinomonadaceae bacterium	NNE65171.1	Bacteria	Acidobacteria	389	
Acidobacteria bacterium	MBM63493.1	Bacteria	Acidobacteria	389	
Acidobacteria bacterium	MAM85618.1	Bacteria	Acidobacteria	403	

Acidobacteria bacterium	PYX47931.1	Bacteria	Acidobacteria	397	
Acidobacteria bacterium	PYR29204.1	Bacteria	Acidobacteria	390	
Chloroflexi bacterium HGW-Chloroflexi-10	PKO19283.1	Bacteria	Chloroflexi	389	
Candidatus Omnitrophica bacterium	NUN95540.1	Bacteria	Candidatus Omnitr	389	
Phycisphaerae bacterium	HGX38596.1	Bacteria	Planctomycetes	386	
Chloroflexi bacterium	NMB57772.1	Bacteria	Chloroflexi	391	
Anaerolineaceae bacterium	HBF40788.1	Bacteria	Chloroflexi	389	
Pelolinea submarina	WP_116224827.1	Bacteria	Chloroflexi	389	
Chloroflexi bacterium	HET59121.1	Bacteria	Chloroflexi	386	
Gemmatimonadetes bacterium	MBW8769261.1	Bacteria	Gemmatimonadete	395	
Gemmatimonadetes bacterium	MBW3628660.1	Bacteria	Gemmatimonadete	407	
Fimbriimonadaceae bacterium	MCC7490884.1	Bacteria	Armatimonadetes	389	
Chloroflexi bacterium	MYB16192.1	Bacteria	Chloroflexi	386	
Kallotenue sp. CFH 73958	WP_152674116.1	Bacteria	Chloroflexi	391	
Chloroflexi bacterium	TMD71227.1	Bacteria	Chloroflexi	402	
Chthonomonadales bacterium	HEG88020.1	Bacteria	Armatimonadetes	389	
Candidatus Poribacteria bacterium	MAE18236.1	Bacteria	Candidatus Poriba	392	
Bryobacteraceae bacterium	HEL71225.1	Bacteria	Acidobacteria	388	
Planctomycetes bacterium	MBI3820577.1	Bacteria	Planctomycetes	408	
Myxococcales bacterium	MCA9670469.1	Bacteria	Proteobacteria	393	
Planctomycetes bacterium	MCC6785626.1	Bacteria	Planctomycetes	394	
Planctomycetes bacterium	MBK8976613.1	Bacteria	Planctomycetes	389	
Alicyclobacillaceae bacterium I2511	RIV28167.1	Bacteria	Firmicutes	386	
Thermoflavifilum sp.	MBX6353464.1	Bacteria	Bacteroidetes	385	
Brevibacillus sp.	REK66843.1	Bacteria	Firmicutes	389	
Clostridia bacterium	MCC3197045.1	Bacteria	Firmicutes	384	
Gemmatimonadetes bacterium	MYC14087.1	Bacteria	Gemmatimonadete	389	
Acidobacteria bacterium	NQW04216.1	Bacteria	Acidobacteria	390	
Acidobacteria bacterium	MBK8317001.1	Bacteria	Acidobacteria	392	
Chloroflexi bacterium	NWJ44451.1	Bacteria	Chloroflexi	389	
Candidatus Melainabacteria bacterium	PWT95842.1	Bacteria	Candidatus Melain	407	
Candidatus Obscuribacterales bacterium	MBX9770056.1	Bacteria	Candidatus Melain	409	
Acidobacteria bacterium	PYU91124.1	Bacteria	Acidobacteria	388	
Acidobacteria bacterium	PYQ17104.1	Bacteria	Acidobacteria	390	
Chloropicon primus	QDZ22434.1	Eukaryotes	Eukaryotes	467	
Micromonas commoda	XP_002506620.1	Eukaryotes	Eukaryotes	411	
Ceratopteris richardii	KAH7441676.1	Eukaryotes	Eukaryotes	453	
Glycine soja	RZB55589.1	Eukaryotes	Eukaryotes	425	
Gossypium hirsutum	KAG4196541.1	Eukaryotes	Eukaryotes	470	
Clostridiaceae bacterium	NLJ94700.1	Bacteria	Firmicutes	439	
Clostridiaceae bacterium	NLJ71241.1	Bacteria	Firmicutes	437	
Fastidiosipila sanguinis	WP_106011734.1	Bacteria	Firmicutes	442	
Oscillospiraceae	WP_079700368.1	Bacteria	Firmicutes	439	
Firmicutes bacterium Adurb.Bin300	OQA48138.1	Bacteria	Firmicutes	442	
Termite gut unspecified	HV438106	Bacteria	Firmicutes	439	Active XI in S. cerevisiae
uncultured symbiotic protist of Reticulitermes sp	BAX90017.1	Eukaryotes	Eukaryotes	440	
Clostridiales bacterium	NLG57270.1	Bacteria	Firmicutes	439	
Firmicutes bacterium	HHU29454.1	Bacteria	Firmicutes	440	
Bacilli bacterium	MBR6056145.1	Bacteria	Firmicutes	441	
Bacilli bacterium	MBQ4183097.1	Bacteria	Firmicutes	439	
Eubacterium sp.	HBZ52894.1	Bacteria	Firmicutes	438	
Clostridia bacterium	MBO5773058.1	Bacteria	Firmicutes	438	
Candidatus Pelethenecus faecipullorum	HIT49725.1	Bacteria	Tenericutes	437	
Spirochaetales bacterium	MBN2651603.1	Bacteria	Spirochaetes	444	
Clostridiales bacterium	MBE7091410.1	Bacteria	Firmicutes	441	
Bacillus timonensis	WP_136380061.1	Bacteria	Firmicutes	439	
Clostridiales bacterium	MBR2749672.1	Bacteria	Firmicutes	442	
Lachnoanaerobaculum saburreum DSM 3986	EFU76426.1	Bacteria	Firmicutes	457	
Uncultured Lachnospira sp. clone XI58444	MK355208.1	Bacteria	Firmicutes	440	Active XI in S. cerevisiae
Clostridiales bacterium	NLC17100.1	Bacteria	Firmicutes	437	
Clostridia bacterium	MBN2878586.1	Bacteria	Firmicutes	437	
Anaerolineaceae bacterium oral taxon 439	AOH42717.1	Bacteria	Chloroflexi	439	
Clostridia bacterium	MBR6935831.1	Bacteria	Firmicutes	441	
Clostridia bacterium	MBQ9415787.1	Bacteria	Firmicutes	441	
Clostridia bacterium	MBQ7338758.1	Bacteria	Firmicutes	439	
Clostridiaceae bacterium	NLB44670.1	Bacteria	Firmicutes	437	
Fastidiosipila sp.	NLC25078.1	Bacteria	Firmicutes	437	
Oscillospiraceae bacterium HV4-5-C5C	NJP40438.1	Bacteria	Firmicutes	437	
Clostridia bacterium	MBQ7822029.1	Bacteria	Firmicutes	437	
Clostridia bacterium	MBP8606590.1	Bacteria	Firmicutes	440	

Clostridiales bacterium	NLZ36408.1	Bacteria	Firmicutes	440	
Clostridiales bacterium	HHT53435.1	Bacteria	Firmicutes	440	
Ructibacterium gallinarum	WP_226392142.1	Bacteria	Firmicutes	439	
Clostridiales bacterium	HBV52300.1	Bacteria	Firmicutes	439	
Clostridiales bacterium	NLP47786.1	Bacteria	Firmicutes	439	
Clostridium cellulosi	CDZ23165.1	Bacteria	Firmicutes	439	
Clostridia bacterium	MBP3322844.1	Bacteria	Firmicutes	439	
Clostridia bacterium	MBO5273775.1	Bacteria	Firmicutes	439	
Clostridia bacterium	MBQ8310334.1	Bacteria	Firmicutes	441	
Oscillospiraceae bacterium	MBQ6430780.1	Bacteria	Firmicutes	439	
Oscillospiraceae bacterium	MBR0352935.1	Bacteria	Firmicutes	437	
Epulopiscium sp. SCG-C07WGA-EpuloA2	ONI45776.1	Bacteria	Firmicutes	438	
Oscillospiraceae bacterium	MBQ4152750.1	Bacteria	Firmicutes	439	
Ndongobacter massiliensis	WP_072515043.1	Bacteria	Firmicutes	442	
Streptobacillus canis	WP_020464968	Bacteria	Proteobacteria	443	Selected XI to express
Anaerosalibacter sp. Marseille-P3206	WP_200804895.1	Bacteria	Firmicutes	440	
Clostridium phytofermentans	BBG40462	Bacteria	Firmicutes	438	Active XI in S. cerevisiae
Eubacterium sp.	MCC8097689.1	Bacteria	Firmicutes	436	
Acetanaerobacterium elongatum	WP_092638076	Bacteria	Firmicutes	437	Selected XI to express
Firmicutes bacterium Adurb. Bin099	OQC01492.1	Bacteria	Firmicutes	442	
Clostridiales bacterium	NTV90666.1	Bacteria	Firmicutes	439	
Anaerolineaceae bacterium	MBG0770425.1	Bacteria	Chloroflexi	439	
Blautia sp.	MBQ8306455.1	Bacteria	Firmicutes	442	
Clostridiaceae bacterium	HBM74193.1	Bacteria	Firmicutes	441	
Hydrogenoanaerobacterium saccharovorans	RPF43242.1	Bacteria	Firmicutes	439	
Ruminococcus callidus ATCC 27760	ERJ95007.1	Bacteria	Firmicutes	461	
Ruminococcus flavefaciens	CAB51938	Bacteria	Firmicutes	438	Active XI in S. cerevisiae
Acholeplasma palmae	WP_026658263.1	Bacteria	Tenericutes	439	
Acholeplasmataceae bacterium	NLH54220.1	Bacteria	Tenericutes	438	
Clostridiaceae bacterium	NLM14926.1	Bacteria	Firmicutes	437	
Clostridia bacterium	MBQ3869938.1	Bacteria	Firmicutes	442	
Acholeplasmatales bacterium	MBQ9520853.1	Bacteria	Tenericutes	435	
Spirochaetales bacterium	MBN2051609.1	Bacteria	Spirochaetes	435	
Candidatus Enterosoma merdigallinarum	HIU70072.1	Bacteria	Firmicutes	440	
Clostridiales bacterium COT073 COT-073	RRD93896.1	Bacteria	Firmicutes	435	
Clostridia bacterium	NCB74957.1	Bacteria	Firmicutes	438	
Culicoidibacter larvae	WP_138191427.1	Bacteria	Firmicutes	438	
Brachyspira intermedia PWS/A	AEM21675.1	Bacteria	Spirochaetes	442	
Lactococcus	WP_021166092.1	Bacteria	Firmicutes	441	
Spiroplasma clarkii	WP_100254008.1	Bacteria	Tenericutes	436	
Erysipelothrix sp. 165301687	WP_159520166.1	Bacteria	Firmicutes	434	
Erysipelothrix sp.	NMB18521.1	Bacteria	Firmicutes	435	
Aerococcus christensenii	WP_060936243.1	Bacteria	Firmicutes	435	
Anaerococcus porci	WP_154540351.1	Bacteria	Firmicutes	435	
Anaerococcus vaginimassiliensis	WP_130819184.1	Bacteria	Firmicutes	435	
Sneathia vaginalis	WP_046328457.1	Bacteria	Fusobacteria	435	
Dolosicoccus paucivorans	WP_102233275.1	Bacteria	Firmicutes	436	
Tetragenococcus halophilus	WP_094243826.1	Bacteria	Firmicutes	435	
Vagococcus salmoninarum	WP_126778109.1	Bacteria	Firmicutes	435	
Erysipelothrix sp. HDW6C	WP_166065452.1	Bacteria	Firmicutes	435	
Enterococcus cecorum	WP_173425970.1	Bacteria	Firmicutes	441	
Facklamia tabacinasalis	WP_138405245.1	Bacteria	Firmicutes	435	
Lacticaseibacillus paracasei	RND43187.1	Bacteria	Firmicutes	379	
Lacticaseibacillus paracasei subsp. paracasei Lp|	EPD12068.1	Bacteria	Firmicutes	450	
Weissella oryzae	WP_027699344.1	Bacteria	Firmicutes	466	
Pediococcus pentosaceus	WP_195749878.1	Bacteria	Firmicutes	447	
Passalid beetle gut—8054_2 (unspecified)	MT846924	Bacteria	Environmental sam	436	Active XI in S. cerevisiae
Paucilactobacillus suebicus DSM 5007 KCTC 35	KRM13063.1	Bacteria	Firmicutes	466	
Propionibacterium australiense	WP_119162843.1	Bacteria	Actinobacteria	451	
Bifidobacterium animalis subsp. animalis MCC (	KOA50460.1	Bacteria	Actinobacteria	469	
Alicyclobacillus ferrooxydans	WP_206658227.1	Bacteria	Firmicutes	439	
Litoribacterium kuwaitense	WP_165203483.1	Bacteria	Firmicutes	437	
Bacillus paralicheniformis	TWL35091.1	Bacteria	Firmicutes	469	
Listeria weihenstephanensis	WP_118907746.1	Bacteria	Firmicutes	435	
Endomicrobium sp.	MCA6071019.1	Bacteria	Elusimicrobia	434	
Paenibacillus sp.	WP_186445855.1	Bacteria	Firmicutes	465	
Cohnella thermotolerans. JC52	WP_027092932.1	Bacteria	Firmicutes	440	
Paenibacillus sp. P22	CDN44317.1	Bacteria	Firmicutes	454	
Paenibacillus tyrfis	KEQ26959.1	Bacteria	Firmicutes	451	
Paenibacillus aquistagni	WP_088548616.1	Bacteria	Firmicutes	438	

Paenibacillus sp.FSL R7-0273	WP_039877260.1	Bacteria	Firmicutes	439	
Alkalicoccus chagannorensis	WP_026697875.1	Bacteria	Firmicutes	444	
Salsuginibacillus kocurii	WP_018922986.1	Bacteria	Firmicutes	437	
Tuberibacillus sp. Marseille-P3662	WP_085523016.1	Bacteria	Firmicutes	441	
Bacillus sp. FJAT-44742	WP_100398771.1	Bacteria	Firmicutes	442	
Marinococcus luteus	WP_091610081.1	Bacteria	Firmicutes	444	
Nosocomiicoccus sp. HMSC09A07	WP_070457711.1	Bacteria	Firmicutes	441	
Alicyclobacillus	WP_072874004.1	Bacteria	Firmicutes	436	
Peribacillus psychrosaccharolyticus	WP_040373941.1	Bacteria	Firmicutes	438	
Bacillus subtilis	SPU04553.1	Bacteria	Firmicutes	448	
Geobacillus proteiniphilus	OKO90731.1	Bacteria	Firmicutes	460	
Geomicrobium sp. JCM 19039	GAK13131.1	Bacteria	Firmicutes	439	
Marinilactibacillus psychrotolerans	WP_087058894.1	Bacteria	Firmicutes	442	
Gracilibacillus boraciitolerans	WP_035721937.1	Bacteria	Firmicutes	442	
Alkalicoccus daliensis	WP_090843444.1	Bacteria	Firmicutes	445	
Marinilactibacillus piezotolerans	WP_080146633.1	Bacteria	Firmicutes	442	
Spirochaeta africana	WP_014456345.1	Bacteria	Spirochaetes	437	
Spirochaetaceae bacterium	TVR68045.1	Bacteria	Spirochaetes	436	
Spirochaetae bacterium HGW-Spirochaetae-3	PKL25228.1	Bacteria	Spirochaetes	430	
Thermotogae bacterium	MBP6982256.1	Bacteria	Thermotogae	441	
Athalassotoga saccharophila	WP_161847301.1	Bacteria	Thermotogae	438	
Petrotoga sp. 9PWA.NaAc.5.4	WP_121958289.1	Bacteria	Thermotogae	438	
candidate division WOR-3 bacterium	MBN1694317.1	Bacteria	candidate division	439	
Fervidobacterium sp.	MBP9518596.1	Bacteria	Thermotogae	441	
Thermotogae bacterium	RKX38304.1	Bacteria	Thermotogae	442	
Fervidobacterium pennivorans	HGQ77747.1	Bacteria	Thermotogae	437	
Marinitoga sp.1135	WP_217671647.1	Bacteria	Thermotogae	440	
Thermosipho sp. in Bacteria	MBO8140535.1	Bacteria	Thermotogae	428	
Kosmotoga sp.	TYB88826.1	Bacteria	Thermotogae	439	
Mesoaciditoga lauensis	WP_036226430.1	Bacteria	Thermotogae	440	
Thermotoga sp.	MBZ4661672.1	Bacteria	Thermotogae	444	
Thermoanaerobacter pseudethanolicus ATCC 3	AAC46145.1	Bacteria	Firmicutes	465	
Caldicellulosiruptor kristjanssonii	WP_013433194.1	Bacteria	Firmicutes	438	Selected XI to express
Kosmotoga sp.	MBO8166794.1	Bacteria	Thermotogae	440	
Thermotogaceae bacterium	HEW92595.1	Bacteria	Thermotogae	438	
Mesotoga sp. H07pep.5.4	WP_121510100.1	Bacteria	Thermotogae	446	
Candidatus Cryosericum terrychapinii	RIE05553.1	Bacteria	Candidatus Cryose	440	
Halobacteriales archaeon AArc-S	QSG01512.1	Archaea	Archaea	439	
Halobacteriales archaeon QH 6 64 20	PSP44228.1	Archaea	Archaea	439	
Halorhabdus tiamatea SARL4B	CCQ34339.1	Archaea	Archaea	448	
Halorhabdus tiamatea	WP_008523802.1	Archaea	Archaea	438	Selected XI to express
Candidatus Bipolaricaulota bacterium	MBS3814107.1	Bacteria	Candidatus Bipolar	442	
Halanaerobiales bacterium	MBS3811649.1	Bacteria	Firmicutes	441	
Halanaerobium saccharolyticum	WP_133514379.1	Bacteria	Firmicutes	439	
Firmicutes bacterium	MTI59082.1	Bacteria	Firmicutes	441	
Candidatus Cloacimonetes bacterium	MBS3766969.1	Bacteria	Candidatus Cloacir	435	
candidate division MSBL1 archaeon SCGC-AAA	KXA98896.1	Archaea	Archaea	439	Selected XI to express
Aminivibrio pyruvatiphilus	WP_133957125.1	Bacteria	Synergistetes	442	
Clostridiales bacterium	HBQ63884.1	Bacteria	Firmicutes	400	
Clostridiales bacterium	PWL53257.1	Bacteria	Firmicutes	440	
Clostridiales bacterium	HHX71877.1	Bacteria	Firmicutes	438	
Candidatus Marispirochaeta associata	WP_069896374.1	Bacteria	Spirochaetes	435	
Vallitalea pronyensis	QUI25646.1	Bacteria	Firmicutes	434	
Firmicutes bacterium	NLO65094.1	Bacteria	Firmicutes	439	
Clostridiales bacterium	NLJ41391.1	Bacteria	Firmicutes	440	
Clostridia bacterium	HGI09347.1	Bacteria	Firmicutes	441	
Anaerobacterium chartisolvens	WP_114296106.1	Bacteria	Firmicutes	439	
Desulfosporosinus acidiphilus	WP_014826083.1	Bacteria	Firmicutes	440	
Clostridiales bacterium	HHU78285.1	Bacteria	Firmicutes	442	
Spirochaeta thermophila	WP_013314408.1	Bacteria	Spirochaetes	440	
Spirochaetaceae bacterium	TVQ23335.1	Bacteria	Spirochaetes	435	
Spirochaetaceae bacterium	TVR63033.1	Bacteria	Spirochaetes	436	
Spirochaetaceae bacterium	TVQ37187.1	Bacteria	Spirochaetes	437	
Spirochaetaceae bacterium	TVQ28199.1	Bacteria	Spirochaetes	437	
Clostridiales bacterium	MBE5816642.1	Bacteria	Firmicutes	439	
Acholeplasma axanthum	WP_026390872.1	Bacteria	Tenericutes	439	
Bacilli bacterium	MBN2540644.1	Bacteria	Firmicutes	439	
Fusobacterium ulcerans	WP_130890766.1	Bacteria	Fusobacteria	436	
Spirochaetes bacterium	MBP8083166.1	Bacteria	Spirochaetes	441	
Bacteroidales bacterium	MBN1181816.1	Bacteria	Bacteroidetes	439	

Clostridia bacterium	MBQ3095671.1	Bacteria	Firmicutes	440	
Spirochaetes bacterium	NOY09700.1	Bacteria	Spirochaetes	437	
Spirochaeta sp.	MQY76975.1	Bacteria	Spirochaetes	440	
Acholeplasmatales bacterium	HCS24431.1	Bacteria	Tenericutes	438	
Acholeplasmatales bacterium	MBQ9124365.1	Bacteria	Tenericutes	435	
Clostridium beijerinckii	AWK51175.1	Bacteria	Firmicutes	441	
Epulopiscium sp.	NLM49014.1	Bacteria	Firmicutes	439	
Clostridiales bacterium oral taxon 876 str. F054(	ERI94441.1	Bacteria	Firmicutes	455	
Firmicutes bacterium	NLY10533.1	Bacteria	Firmicutes	439	
Epulopiscium sp.	HHX62016.1	Bacteria	Firmicutes	443	
Clostridia bacterium	MBQ6818653.1	Bacteria	Firmicutes	439	
Lachnospiraceae bacterium	HAB61884.1	Bacteria	Firmicutes	447	
Clostridiales bacterium 38-18	OJV66811.1	Bacteria	Firmicutes	437	
Vallitaleaceae bacterium	MBN2220663.1	Bacteria	Firmicutes	439	
Bacillus sp. FJAT-29953	WP_217032784.1	Bacteria	Firmicutes	452	
Crassaminicella profunda	WP_223036963.1	Bacteria	Firmicutes	439	
Oceanivirga salmonicida	WP_156285771.1	Bacteria	Fusobacteria	440	
Clostridioides difficile 002-P50-2011	EHJ32437.1	Bacteria	Firmicutes	454	
Chitinivibrionales bacterium	MBD3420666.1	Bacteria	Fibrobacteres	437	
Acanthamoeba castellanii str. Neff	XP_004356776.1	Eukaryotes	Eukaryotes	437	
Fibrobacteres bacterium	HAO99316.1	Bacteria	Fibrobacteres	435	
Blastocystis sp. subtype 4	XP_014524855.1	Eukaryotes	Eukaryotes	448	
Polarella glacialis	CAE8610118.1	Eukaryotes	Eukaryotes	449	
Hondaea fermentalgiana	GBG28639.1	Eukaryotes	Eukaryotes	457	
Synchytrium endobioticum	TPX44438.1	Eukaryotes	Eukaryotes	468	
Chytriomyces confervae	TPX77282.1	Eukaryotes	Eukaryotes	445	
Rhizoclosmatium globosum	ORY49883.1	Eukaryotes	Eukaryotes	407	Selected XI to express
Cafeteria roenbergensis	KAA0145869.1	Eukaryotes	Eukaryotes	462	
Symbiodinium sp. KB8	CAE7655680.1	Eukaryotes	Eukaryotes	431	
Phytophthora infestans T30-4	XP_002904118.1	Eukaryotes	Eukaryotes	469	
Aphanomyces cochlioides	KAG9415118.1	Eukaryotes	Eukaryotes	435	
Fragilariopsis cylindrus CCMP1102	OEU15264.1	Eukaryotes	Eukaryotes	444	
Phaeodactylum tricornutum CCAP 1055/1	XP_002184902.1	Eukaryotes	Eukaryotes	445	
Fistulifera solaris	GAX11382.1	Eukaryotes	Eukaryotes	447	
Nannochloropsis salina CCMP1776	TFJ80291.1	Eukaryotes	Eukaryotes	452	
Pelomyxa schiedti	KAH3759417.1	Eukaryotes	Eukaryotes	457	
Rotaria sordida	CAF1166178.1	Eukaryotes	Eukaryotes	461	
BOX15 Mlig030170g1 Macrostomum lignano	PAA55115.1	Eukaryotes	Eukaryotes	441	
Daphnia magna	XP_032797976.1	Eukaryotes	Eukaryotes	459	
Penaeus japonicus	XP_042858672.1	Eukaryotes	Eukaryotes	454	
Pollicipes pollicipes	XP_037078508.1	Eukaryotes	Eukaryotes	459	
Gigantopelta aegis	XP_041378737.1	Eukaryotes	Eukaryotes	460	
Lottia gigantea	XP_009053421.1	Eukaryotes	Eukaryotes	461	
Pecten maximus	XP_033736947.1	Eukaryotes	Eukaryotes	467	
Candidula unifasciata	CAG5117749.1	Eukaryotes	Eukaryotes	461	
Pangasianodon hypophthalmus	XP_034153774.1	Eukaryotes	Eukaryotes	443	
Salmo trutta	XP_029599031.1	Eukaryotes	Eukaryotes	470	
Dimorphilus gyrociliatus	CAD5126168.1	Eukaryotes	Eukaryotes	444	
Acanthaster planci	XP_022089489.1	Eukaryotes	Eukaryotes	460	
Branchiostoma belcheri	XP_019645181.1	Eukaryotes	Eukaryotes	458	
Styela clava	XP_039271074.1	Eukaryotes	Eukaryotes	469	
Lingula anatina	XP_013404708.1	Eukaryotes	Eukaryotes	457	
Planctomycetes bacterium	TVR14229.1	Bacteria	Planctomycetes	439	
Planctomycetes bacterium	MBA3710670.1	Bacteria	Planctomycetes	445	
Puniceicoccaceae bacterium	TVR46478.1	Bacteria	Verrucomicrobia	439	
Cephaloticoccus capnophilus	WP_068711694.1	Bacteria	Verrucomicrobia	455	
Chthoniobacterales bacterium	MBE2205676.1	Bacteria	Verrucomicrobia	450	
Alphaproteobacteria bacterium MarineAlpha5 E	PPR45514.1	Bacteria	Proteobacteria	437	
Alphaproteobacteria bacterium	MBT4911748.1	Bacteria	Proteobacteria	436	
Pelagibacteraceae bacterium	MAH78202.1	Bacteria	Proteobacteria	435	
Gammaproteobacteria bacterium	RCL43797.1	Bacteria	Proteobacteria	435	
Coxiella sp. DG 40	KPJ67902.1	Bacteria	Proteobacteria	444	
uncultured bacterium	EKE08639.1	Bacteria	Environmental san	438	
Gammaproteobacteria bacterium	MSP53168.1	Bacteria	Proteobacteria	438	
Chlamydiia bacterium	MCB1111506.1	Bacteria	Chlamydiae	445	
Coxiella burnetii	WP_005769174.1	Bacteria	Proteobacteria	436	
Gammaproteobacteria bacterium RIFCSPHIGHO	OGT39069.1	Bacteria	Proteobacteria	433	
Microthrixaceae bacterium	MCB1012300.1	Bacteria	Actinobacteria	453	
Actinomycetia bacterium	MBW3603811.1	Bacteria	Actinobacteria	448	
Acidimicrobiales bacterium	MBV6508023.1	Bacteria	Actinobacteria	453	

Ilumatobacter sp.	TVR25937.1	Bacteria	Actinobacteria	463	
Ilumatobacter sp.	MBC48644.1	Bacteria	Actinobacteria	447	
Acidimicrobiia bacterium	MBT8240085.1	Bacteria	Actinobacteria	443	
Acidimicrobiales bacterium	HEC08044.1	Bacteria	Actinobacteria	444	
Nitriliruptoria bacterium AS10	MBY5162015.1	Bacteria	Actinobacteria	441	
Acidimicrobiaceae bacterium	MAM31134.1	Bacteria	Actinobacteria	446	
Gammaproteobacteria bacterium	MYJ76225.1	Bacteria	Proteobacteria	438	
Acidimicrobiales bacterium	MBL8776453.1	Bacteria	Actinobacteria	443	
Euzebya pacifica	WP_114590165.1	Bacteria	Actinobacteria	441	
Acidimicrobiia bacterium	MBO2499937.1	Bacteria	Actinobacteria	445	
Chloroflexi bacterium	MBA2721122.1	Bacteria	Chloroflexi	467	
Chloroflexi bacterium	TME93739.1	Bacteria	Chloroflexi	465	
Chloroflexi bacterium RBG 16 72 14	OGO56541.1	Bacteria	Chloroflexi	450	
Nitriliruptoraceae bacterium ZYF776	MTV25645.1	Bacteria	Actinobacteria	454	
Acidimicrobiia bacterium	NNC91837.1	Bacteria	Actinobacteria	445	
Nitriliruptor alkaliphilus	WP_052668689.1	Bacteria	Actinobacteria	452	
Acidimicrobiaceae bacterium	MXW76933.1	Bacteria	Actinobacteria	443	
Actinobacteria bacterium IMCC26207	KLR60470.1	Bacteria	Actinobacteria	453	
Parasphingopyxis algicola	WP_176869054.1	Bacteria	Proteobacteria	438	
Rhodospirillales bacterium	MBE0532122.1	Bacteria	Proteobacteria	458	
Hyphomicrobiales bacterium	PLX34087.1	Bacteria	Proteobacteria	438	
Hyphomicrobiales bacterium	MBX2804730.1	Bacteria	Proteobacteria	445	
Rhodospirillales bacterium	NQV99973.1	Bacteria	Proteobacteria	436	
Rhodospirillales bacterium	MBL6931588.1	Bacteria	Proteobacteria	437	
Inquilinus sp.	NNG02616.1	Bacteria	Proteobacteria	437	
Deinococcus-Thermus bacterium	NBC96068.1	Bacteria	Deinococcus-Therı	437	
Alphaproteobacteria bacterium	MBN9509595.1	Bacteria	Proteobacteria	435	
Bosea sp. SSUT16	WP_142022331.1	Bacteria	Proteobacteria	435	
Alphaproteobacteria bacterium	MBN9525130.1	Bacteria	Proteobacteria	437	
Xenopus tropicalis	XP_017946154.1	Eukaryotes	Eukaryotes	467	
Roseomonas ponticola	WP_203077000.1	Bacteria	Proteobacteria	437	
Deltaproteobacteria bacterium	HAC78748.1	Bacteria	Proteobacteria	438	
Azospirillum oleiclasticum	WP_180284716.1	Bacteria	Proteobacteria	436	
Stigmatella aurantiaca	WP_002615196.1	Bacteria	Proteobacteria	438	
Myxococcaceae bacterium	CAG0954897.1	Bacteria	Proteobacteria	438	
Deltaproteobacteria bacterium	MBJ20920.1	Bacteria	Proteobacteria	445	
Deltaproteobacteria bacterium	MBW1685260.1	Bacteria	Proteobacteria	439	
Deltaproteobacteria bacterium	MAG29608.1	Bacteria	Proteobacteria	440	
Deltaproteobacteria bacterium	MBW2293084.1	Bacteria	Proteobacteria	442	
Deltaproteobacteria bacterium	MBW2270299.1	Bacteria	Proteobacteria	448	
Acidobacteria bacterium	MYN67984.1	Bacteria	Acidobacteria	439	
Deltaproteobacteria bacterium	MBW1883432.1	Bacteria	Proteobacteria	439	
Deltaproteobacteria bacterium	MBW2273769.1	Bacteria	Proteobacteria	439	
Deltaproteobacteria bacterium	MBM4268705.1	Bacteria	Proteobacteria	438	
Candidatus Binatia bacterium	MBY0276224.1	Bacteria	Candidatus Binatot	442	
Deltaproteobacteria bacterium	MBI3785828.1	Bacteria	Proteobacteria	439	
Lichenihabitans	WP_131113834.1	Bacteria	Proteobacteria	434	
Lichenicoccus roseus	WP_138323949.1	Bacteria	Proteobacteria	435	
Telmatospirillum siberiense	WP_101250217.1	Bacteria	Proteobacteria	437	
Terracidiphilus gabretensis	WP_058189414.1	Bacteria	Acidobacteria	442	
Bryocella elongata	WP_103935371.1	Bacteria	Acidobacteria	443	
Endobacter medicaginis	WP_176626062.1	Bacteria	Proteobacteria	439	
Granulicella sibirica	WP_128913632.1	Bacteria	Acidobacteria	439	
Betaproteobacteria bacterium AqS2	MBF2734955.1	Bacteria	Proteobacteria	439	
Nordella sp. HKS 07	WP_165167696.1	Bacteria	Proteobacteria	437	
Aestuariivirga litoralis	WP_196503738.1	Bacteria	Proteobacteria	435	
Aestuariivirga sp.	MCA3559532.1	Bacteria	Proteobacteria	437	
Siculibacillus lacustris	WP_131306160.1	Bacteria	Proteobacteria	441	
Aureimonas ureilytica	WP_019995944.1	Bacteria	Proteobacteria	440	
Bradyrhizobium sp. ORS 285	CCD86673.1	Bacteria	Proteobacteria	453	
Alphaproteobacteria bacterium	RYY14481.1	Bacteria	Proteobacteria	437	Selected XI to express
Piscinibacter sp.	MBX3607287.1	Bacteria	Proteobacteria	457	
Burkholderiales bacterium	MBI5719309.1	Bacteria	Proteobacteria	459	
Acidisoma silvae	WP_227319592.1	Bacteria	Proteobacteria	440	
Arenibaculum pallidiluteum	WP_207479092.1	Bacteria	Proteobacteria	447	
Lutibaculum baratangense	WP_023430420.1	Bacteria	Proteobacteria	436	
Oharaeibacter diazotrophicus	WP_126538087.1	Bacteria	Proteobacteria	438	
Hyphomicrobiales bacterium	MBN8996470.1	Bacteria	Proteobacteria	438	
Magnetospira sp. QH-2	WP_046022288.1	Bacteria	Proteobacteria	436	
Leucothrix pacifica	PWQ97442.1	Bacteria	Proteobacteria	438	

Bauldia sp.	MCC6984693.1	Bacteria	Proteobacteria	437
Methylobacteriaceae bacterium	MBV9219372.1	Bacteria	Proteobacteria	437
Devosia sp.	MBK8084728.1	Bacteria	Proteobacteria	436
Bradyrhizobium sp.	THD42015.1	Bacteria	Proteobacteria	437
Hyphomicrobiales bacterium	PCJ89455.1	Bacteria	Proteobacteria	437
SAR116 cluster bacterium	RZO30786.1	Bacteria	Proteobacteria	441
Alphaproteobacteria bacterium	GIR78741.1	Bacteria	Proteobacteria	438
Candidatus Puniceispirillum sp. TMED52	OUU55909.1	Bacteria	Proteobacteria	441
Kiloniella sp.	MAH88274.1	Bacteria	Proteobacteria	438
Rhodovulum sulfidophilum	WP_202259578.1	Bacteria	Proteobacteria	435
Devosia sp.	HHG90593.1	Bacteria	Proteobacteria	435
Stappiaceae	WP_022999245.1	Bacteria	Proteobacteria	439
Hyphomicrobiales bacterium	MBL1406768.1	Bacteria	Proteobacteria	439
Rhodovulum sp.	MAY31865.1	Bacteria	Proteobacteria	437
Rhodoligotrophos appendicifer	WP_144291700.1	Bacteria	Proteobacteria	436
Mesorhizobium sp.	TIU21811.1	Bacteria	Proteobacteria	421
Lupinus albus	KAF1853601.1	Eukaryote:	Eukaryotes	464
Poseidonocella sp. HB161398	WP_138469546.1	Bacteria	Proteobacteria	439
Rhodobacteraceae bacterium	RZW12574.1	Bacteria	Proteobacteria	437
Pseudooceanicola sp. HF7	WP_175581768.1	Bacteria	Proteobacteria	441
Cereibacter sphaeroides	QHA11673.1	Bacteria	Proteobacteria	458
Limimaricola cinnabarinus LL-001	GAD56635.1	Bacteria	Proteobacteria	448
Hwanghaeicola aestuarii	WP_111536756.1	Bacteria	Proteobacteria	433
Hasllibacter halocynthiae	WP_106159297.1	Bacteria	Proteobacteria	432
Profundibacterium mesophilum	WP_159964173.1	Bacteria	Proteobacteria	434
Sulfitobacter noctilucicola	WP_025056156.1	Bacteria	Proteobacteria	431
Lentibacter algarum	WP_215959042.1	Bacteria	Proteobacteria	432
Pseudorhodobacter sp. E13	WP_199743710.1	Bacteria	Proteobacteria	437
Rhodobacter sp. 24-YEA-8	WP_092895636.1	Bacteria	Proteobacteria	438
partial Candidatus Hydrogenedentes bacterium	PCJ49819.1	Bacteria	Candidatus Hydrog	418
Fibrobacteres bacterium	MBW8890079.1	Bacteria	Fibrobacteres	438
Myxococcales bacterium	MBV9946703.1	Bacteria	Proteobacteria	440
Deltaproteobacteria bacterium	MBN2576139.1	Bacteria	Proteobacteria	439
Propionivibrio sp.	MBP6655241.1	Bacteria	Proteobacteria	438
Candidatus Sulfopaludibacter sp. SbA3	SPE25015.1	Bacteria	Acidobacteria	438
Rhizomicrobium electricum	WP_166935833.1	Bacteria	Proteobacteria	438
Gammaproteobacteria bacterium	MCC7462827.1	Bacteria	Proteobacteria	438
Sinobacteraceae bacterium	MBV8805586.1	Bacteria	Proteobacteria	438
Hirschia baltica	WP_015828648.1	Bacteria	Proteobacteria	438
Parvularculaceae bacterium	NRA30521.1	Bacteria	Proteobacteria	437
Hyphomonas atlantica	HBQ48126.1	Bacteria	Proteobacteria	448
Henriciella sp.	MBO6696385.1	Bacteria	Proteobacteria	438
Hyphomonas sp.	HAY06353.1	Bacteria	Proteobacteria	436
Parvularcula dongshanensis	WP_183819010.1	Bacteria	Proteobacteria	439
Alphaproteobacteria bacterium	MBI1365435.1	Bacteria	Proteobacteria	439
Myxococcales bacterium	MBX2810310.1	Bacteria	Proteobacteria	442
Sphingomonas bacterium	WP_174292994.1	Bacteria	Proteobacteria	435
Sphingomonas oligoaromativorans	NIJ32218.1	Bacteria	Proteobacteria	449
Novosphingobium sp. 17-62-8	OYZ97817.1	Bacteria	Proteobacteria	400
Sphingomonadales bacterium	MBU6268161.1	Bacteria	Proteobacteria	446
Alphaproteobacteria bacterium	RME61863.1	Bacteria	Proteobacteria	438
Alphaproteobacteria bacterium	TNE65834.1	Bacteria	Proteobacteria	446
Gammaproteobacteria bacterium	MBO2489166.1	Bacteria	Proteobacteria	448
Amphiplicatus metriothermophilus	WP_089412709.1	Bacteria	Proteobacteria	439
Robiginitomaculum sp.	PHR92924.1	Bacteria	Proteobacteria	439
Asticcacaulis solisilvae	WP_209719649.1	Bacteria	Proteobacteria	438
Erythrobacter sp. KY5	WP_111992204.1	Bacteria	Proteobacteria	437
Erythrobacter sp. WH158	WP_218405326.1	Bacteria	Proteobacteria	439
Altererythrobacter rhizovicinus	WP_129522860.1	Bacteria	Proteobacteria	438
Cocleimonas flava	WP_131907615.1	Bacteria	Proteobacteria	437
Alphaproteobacteria bacterium	MBL1418955.1	Bacteria	Proteobacteria	437
Uliginosibacterium sp.	MBK9217046.1	Bacteria	Proteobacteria	438
Deltaproteobacteria bacterium	TMB15958.1	Bacteria	Proteobacteria	437
Deltaproteobacteria bacterium	MBN1961441.1	Bacteria	Proteobacteria	437
Rhodoferax sp.	MBL8335154.1	Bacteria	Proteobacteria	436
Massilia sp. HP4	WP_137174199.1	Bacteria	Proteobacteria	442
Inhella sp.	MBP8144491.1	Bacteria	Proteobacteria	440
Rhizobacter sp.	MBX3624499.1	Bacteria	Proteobacteria	437
Comamonadaceae bacterium PBBC2	OYU30777.1	Bacteria	Proteobacteria	439
Pseudorivibacter rhizosphaerae	WP_140630593.1	Bacteria	Proteobacteria	440

Burkholderiales bacterium	TAG27410.1	Bacteria	Proteobacteria	440	
Shewanella livingstonensis	WP_124730814.1	Bacteria	Proteobacteria	439	
Natronospirillum operosum	WP_135484387.1	Bacteria	Proteobacteria	441	
Reinekea blandensis	WP_008042520.1	Bacteria	Proteobacteria	443	
Nicoletella semolina	WP_132500856.1	Bacteria	Proteobacteria	439	
Celerinatantimonas diazotrophica	WP_131911963.1	Bacteria	Proteobacteria	438	
Commensalibacter sp. MX01	WP_034338385.1	Bacteria	Proteobacteria	439	
Pararobbsia alpina	CAB3790154.1	Bacteria	Proteobacteria	455	
Paraburkholderia sabiae	CAG9212793.1	Bacteria	Proteobacteria	457	
Burkholderia cenocepacia	CAR57287.1	Bacteria	Proteobacteria	440	Active XI in S. cerevisiae
Telmatospirillum sp.	MTJ83190.1	Bacteria	Proteobacteria	436	
Luteibacter pinisoli	WP_211352762.1	Bacteria	Proteobacteria	448	
Polaromonas sp. A23	WP_077592566.1	Bacteria	Proteobacteria	441	
Pseudomonas syringae pv. ribicola	KPY46556.1	Bacteria	Proteobacteria	465	
Acidovorax wautersii	KAF1023998.1	Bacteria	Proteobacteria	450	
Gammaproteobacteria bacterium	GBU09853.1	Bacteria	Proteobacteria	439	
Shewanella	WP_080915560.1	Bacteria	Proteobacteria	438	
Tenacibaculum sp. KUL113	GFD73863.1	Bacteria	Bacteroidetes	454	
Marinomonas profundi	WP_168822205.1	Bacteria	Proteobacteria	439	
Glaciecola sp. KUL10	GBL04291.1	Bacteria	Proteobacteria	450	
Agarivorans gilvus	WP_055733745.1	Bacteria	Proteobacteria	440	
Vibrio breoganii	NMO75251.1	Bacteria	Proteobacteria	468	
Exilibacterium tricleocarpae	WP_142904150.1	Bacteria	Proteobacteria	439	
Marinobacterium ramblicola	WP_217412564.1	Bacteria	Proteobacteria	438	
Klebsiella pneumoniae	WP_004872522.1	Bacteria	Proteobacteria	408	
XylA Serratia plymuthica 4Rx13	AGO53094.1	Bacteria	Proteobacteria	463	
Beauveria bassiana D1-5	KGQ13041.1	Eukaryotes	Eukaryotes	440	Selected XI to express
Planctomycetes bacterium	HAT09524.1	Bacteria	Planctomycetes	444	
Methyloprofundus sp.	BCG63003.1	Bacteria	Proteobacteria	443	
Acidobacteria bacterium	TDI42666.1	Bacteria	Acidobacteria	442	
Planctomycetes bacterium	MBS3762923.1	Bacteria	Planctomycetes	441	
partial bacterium M21	OVE80921.1	Bacteria	Unclassified Bacter	426	
Holophagales bacterium	MXW03024.1	Bacteria	Acidobacteria	447	
Deltaproteobacteria bacterium	MBI5528035.1	Bacteria	Proteobacteria	443	
Planctomycetes bacterium	MAE28239.1	Bacteria	Planctomycetes	443	
Ignavibacteriales bacterium	MBD3409486.1	Bacteria	Ignavibacteriae	438	
candidate division KSB1 bacterium	MBD3288715.1	Bacteria	Unclassified Bacter	441	
bacterium	HEU34634.1	Bacteria	Unclassified Bacter	443	
Candidatus Handelsmanbacteria bacterium	MBM3278896.1	Bacteria	Candidatus Handel	438	
Lentisphaerae bacterium	MBM4144543.1	Bacteria	Lentisphaerae	440	
Lentisphaerae bacterium RIFOXYA12 FULL 48 1	OGV57234.1	Bacteria	Lentisphaerae	440	
Lentisphaerae bacterium	MBM4149798.1	Bacteria	Lentisphaerae	432	
Desulfovibrio sp.	THB64761.1	Bacteria	Proteobacteria	436	
Pseudodesulfovibrio sp. SF6	BCS89731.1	Bacteria	Proteobacteria	435	
SAR324 cluster bacterium	MBF0278152.1	Bacteria	Proteobacteria	437	
Alkalispirochaeta odontotermitis	WP_037562869.1	Bacteria	Spirochaetes	437	
bacterium	MBU2643774.1	Bacteria	Unclassified Bacter	437	
candidate division KSB1 bacterium	MCA9732525.1	Bacteria	Unclassified Bacter	438	
Ignavibacteriae bacterium	MBK7259581.1	Bacteria	Ignavibacteriae	437	
Bacteroidetes bacterium	HCV42381.1	Bacteria	Bacteroidetes	441	
bacterium Adurb.Bin478	OPZ72934.1	Bacteria	Unclassified Bacter	448	
Deltaproteobacteria bacterium	MBN1831696.1	Bacteria	Proteobacteria	441	
Lentisphaerae bacterium GWF2 38 69	OGV17837.1	Bacteria	Lentisphaerae	439	
Bacteroidales bacterium	MBI9036888.1	Bacteria	Bacteroidetes	438	
Lentisphaerae bacterium	QSH42615.1	Bacteria	Lentisphaerae	436	
Victivallales bacterium	MBU8902100.1	Bacteria	Lentisphaeria	438	
Lentisphaeria bacterium	NQZ67148.1	Bacteria	Lentisphaerae	436	
Lentisphaeria bacterium	HCN10411.1	Bacteria	Lentisphaerae	437	
Desulfosediminicola flagellatus	WP_136810460.1	Bacteria	Proteobacteria	436	
Desulfopila sp. IMCC35008	WP_163337432.1	Bacteria	Proteobacteria	437	
Deltaproteobacteria bacterium	RLB90146.1	Bacteria	Proteobacteria	437	
Methanoregulaceae archaeon	MCC7433628.1	Archaea	Archaea	424	
bacterium	RYG47248.1	Bacteria	Unclassified Bacter	442	
Chlorobia bacterium	MBC8065216.1	Bacteria	Unclassified Bacter	437	
Planctomycetales bacterium zrk34	QNN25081.1	Bacteria	Planctomycetes	437	
Phycisphaera sp.	MBI1337614.1	Bacteria	Planctomycetes	437	
Phycisphaera mikurensis	WP_014436547.1	Bacteria	Planctomycetes	437	
Phycisphaeraceae bacterium	RZO56748.1	Bacteria	Planctomycetes	441	
Planctomycetes bacterium Pan265	QDU72235.1	Bacteria	Planctomycetes	436	
Planctomycetes bacterium	NOZ21217.1	Bacteria	Planctomycetes	437	

Sedimentisphaerales bacterium	MBN1765309.1	Bacteria	Planctomycetes	438	
Phycisphaerae bacterium	MBI9019043.1	Bacteria	Planctomycetes	436	
Phycisphaerales bacterium	MCB9850719.1	Bacteria	Planctomycetes	438	
Candidatus Sumerlaeia bacterium	MBX3727814.1	Bacteria	Candidatus Sumerl	441	
Kiritimatiella glycovorans	WP_052881091.1	Bacteria	Kiritimatiellaeota	437	
Armatimonadetes bacterium	NCO39587.1	Bacteria	Armatimonadetes	436	
Planctomycetes bacterium GWF2 41 51	OHB52044.1	Bacteria	Planctomycetes	436	
bacterium	MBI1387917.1	Bacteria	Unclassified Bacter	441	
Candidatus Hydrogenedentes bacterium	MCC6144355.1	Bacteria	Candidatus Hydrog	436	
Candidatus Hydrogenedentes bacterium	MBP7430745.1	Bacteria	Candidatus Hydrog	437	
Candidatus Hydrogenedentes bacterium	HDP34382.1	Bacteria	Candidatus Hydrog	440	
Phycisphaerae bacterium	NLU22726.1	Bacteria	Planctomycetes	441	
Planctomycetes bacterium	MBM4041428.1	Bacteria	Planctomycetes	422	
Planctomycetes bacterium	NBB94843.1	Bacteria	Planctomycetes	435	
Phycisphaeraceae bacterium	MCC6681562.1	Bacteria	Planctomycetes	437	
Pirellulales bacterium	MBN2215855.1	Bacteria	Planctomycetes	437	
Candidatus Poribacteria bacterium	MBT3266112.1	Bacteria	Candidatus Poribac	438	
Limnoglobus roseus	WP_149109417.1	Bacteria	Planctomycetes	439	
Planctomycetes bacterium Pan216	QDU59467.1	Bacteria	Planctomycetes	438	
Gemmataceae bacterium	MBY0229415.1	Bacteria	Planctomycetes	439	
Rosistilla oblonga	QDV54489.1	Bacteria	Planctomycetes	466	
Planctomycetaceae bacterium	HBH53523.1	Bacteria	Planctomycetes	441	
Planctomycetaceae bacterium	PHY03599.1	Bacteria	Planctomycetes	444	
Candidatus Peribacteria bacterium RIFCSPHIGH	OGJ62933.1	Bacteria	Candidatus Peregri	443	
Thermoguttaceae bacterium	MBQ1455546.1	Bacteria	Planctomycetes	439	
Acidobacteria bacterium	NOT60167.1	Bacteria	Acidobacteria	437	
Verrucomicrobia bacterium	MBN8246088.1	Bacteria	Verrucomicrobia	441	
Verrucomicrobia bacterium	MBR5605839.1	Bacteria	Verrucomicrobia	451	
Armatimonadia bacterium	MBD3178054.1	Bacteria	Armatimonadetes	438	
Sedimentisphaerales bacterium	MBN1816769.1	Bacteria	Planctomycetes	444	
Phycisphaerae bacterium	HDZ22346.1	Bacteria	Planctomycetes	437	
Planctomycetaceae bacterium	MBV8230502.1	Bacteria	Planctomycetes	442	
Opitutales bacterium	MCC5790848.1	Bacteria	Verrucomicrobia	439	
Candidatus Moanabacter tarae	AWT59279.1	Bacteria	Verrucomicrobia	438	
Chthoniobacter flavus	WP_006978554.1	Bacteria	Verrucomicrobia	437	
Verrucomicrobia bacterium	MBV8173828.1	Bacteria	Verrucomicrobia	440	
Acidobacteria bacterium	PYX28907.1	Bacteria	Acidobacteria	454	
Verrucomicrobia bacterium Adurb.Bin006	OQC67092.1	Bacteria	Verrucomicrobia	453	
Verrucomicrobia bacterium 21-51-4	OYV48436.1	Bacteria	Verrucomicrobia	435	
Ruficoccus sp. ZRK36	WP_220621034.1	Bacteria	Verrucomicrobia	435	
Puniceicoccaceae bacterium 5H	KAF0095473.1	Bacteria	Verrucomicrobia	452	
Verrucomicrobia bacterium	MCC5843155.1	Bacteria	Verrucomicrobia	435	
Spartobacteria bacterium LR76	PTX96241.1	Bacteria	Verrucomicrobia	437	
Phycisphaerae bacterium	MBC23348.1	Bacteria	Planctomycetes	436	
Verrucomicrobia bacterium	NBV85433.1	Bacteria	Verrucomicrobia	441	
Opitutales bacterium	MCC5835523.1	Bacteria	Verrucomicrobia	437	
Verrucomicrobiales bacterium	MBT15508.1	Bacteria	Verrucomicrobia	441	
Opitutae bacterium	MBL63346.1	Bacteria	Verrucomicrobia	437	
Armatimonadetes bacterium	MBC7980528.1	Bacteria	Armatimonadetes	435	
Puniceicoccus vermicola	WP_185694763.1	Bacteria	Verrucomicrobia	435	
bacterium	NBR48611.1	Bacteria	Unclassified Bacter	437	
Phycisphaera sp. TMED9	RPG14701.1	Bacteria	Planctomycetes	437	
Planctomycetaceae bacterium	MAJ47367.1	Bacteria	Planctomycetes	438	
Phycisphaerae bacterium	MBG79635.1	Bacteria	Planctomycetes	437	
Phycisphaerales bacterium	HCA38938.1	Bacteria	Planctomycetes	436	
Phycisphaerae bacterium	MBM43960.1	Bacteria	Planctomycetes	437	
Phycisphaerae bacterium	MAB82258.1	Bacteria	Planctomycetes	438	
Planctomycetaceae bacterium	PHX78033.1	Bacteria	Planctomycetes	443	
Planctomycetes bacterium	RLS61848.1	Bacteria	Planctomycetes	437	
Phycisphaerae bacterium	MBL9150588.1	Bacteria	Planctomycetes	456	
Phycisphaerae bacterium	MBM4106070.1	Bacteria	Planctomycetes	437	
Phycisphaeraceae bacterium	MBX3356555.1	Bacteria	Planctomycetes	437	
Phycisphaerae bacterium	MBM4111849.1	Bacteria	Planctomycetes	441	
Phycisphaerae bacterium	MBM4110909.1	Bacteria	Planctomycetes	441	
Planctomycetes bacterium	NDG61929.1	Bacteria	Planctomycetes	443	
Phycisphaerales bacterium	MSR44345.1	Bacteria	Planctomycetes	459	
Phycisphaerales bacterium	MSR29455.1	Bacteria	Planctomycetes	450	
Alkalispirochaeta americana	WP_076488533.1	Bacteria	Spirochaetes	455	
candidate division KSB1 bacterium	MCA9730992.1	Bacteria	Unclassified Bacter	439	Selected XI to express
Candidatus Marinimicrobia bacterium	MBT5269314.1	Bacteria	Candidatus Marinir	439	

candidate division KSB1 bacterium	MBD3384725.1	Bacteria	Unclassified Bacter	443	
Acidobacteria bacterium	MBM52388.1	Bacteria	Acidobacteria	444	
Calditrichaeota bacterium	KAA3612560.1	Bacteria	Calditrichaeota	445	
Candidatus Marinimicrobia bacterium	MAV96254.1	Bacteria	Candidatus Marinir	443	
Candidatus Marinimicrobia bacterium	MAQ87721.1	Bacteria	Candidatus Marinir	442	
Candidatus Marinimicrobia bacterium	GIR30577.1	Bacteria	Candidatus Marinir	445	
Candidatus Marinimicrobia bacterium	MBO8130993.1	Bacteria	Candidatus Marinir	445	
Candidatus Marinimicrobia bacterium	MBN2279138.1	Bacteria	Candidatus Marinir	445	
Calditrichaeota bacterium	RPH91893.1	Bacteria	Calditrichaeota	444	
bacterium	MBN1779537.1	Bacteria	Unclassified Bacter	446	
candidate division KSB3 bacterium	PID60038.1	Bacteria	Unclassified Bacter	444	
Actinomycetia bacterium	NIA31423.1	Bacteria	Actinobacteria	442	
Spirochaetes bacterium	MBN8217625.1	Bacteria	Spirochaetes	446	
Spirochaetia bacterium	MBL8992510.1	Bacteria	Spirochaetes	441	
Lentisphaera araneosa	WP_007276628.1	Bacteria	Lentisphaerae	436	
Deltaproteobacteria bacterium	MBM4251139.1	Bacteria	Proteobacteria	445	
Bacteroidetes bacterium	NBC25386.1	Bacteria	Bacteroidetes	440	
Balneolaceae bacterium	MCC5941015.1	Bacteria	Balneolaeota	440	
Planctomycetes bacterium	PCJ59489.1	Bacteria	Planctomycetes	445	
Deltaproteobacteria bacterium	MBN2801666.1	Bacteria	Proteobacteria	448	
Deltaproteobacteria bacterium	MBN2528967.1	Bacteria	Proteobacteria	444	
Kordiimonadaceae bacterium	MBT5765919.1	Bacteria	Proteobacteria	447	
Luminiphilus sp.	MBL6694207.1	Bacteria	Proteobacteria	449	
Halieaceae bacterium	MAP04176.1	Bacteria	Proteobacteria	450	
Arenicella xantha	WP_113954423.1	Bacteria	Proteobacteria	447	
Alteromonadaceae bacterium	PCK03603.1	Bacteria	Proteobacteria	447	
Veronia nyctiphanis	RXJ74060.1	Bacteria	Proteobacteria	448	
Povalibacter uvarum	WP_184331996.1	Bacteria	Proteobacteria	446	
partial Xanthomonas sp. Leaf131	KQQ71703.1	Bacteria	Proteobacteria	425	
Rhodanobacter sp.115	EIL91239.1	Bacteria	Proteobacteria	468	
Haliea sp.	MBK6510872.1	Bacteria	Proteobacteria	444	
Wenzhouxiangella sp. W260	WP_150863339.1	Bacteria	Proteobacteria	450	
Gammaproteobacteria bacterium	NOX69598.1	Bacteria	Proteobacteria	446	
Acanthopleuribacter pedis	WP_207863592.1	Bacteria	Acidobacteria	447	
Xanthomonadales bacterium	RPH98085.1	Bacteria	Proteobacteria	450	
Thalassobius sp.	MBT30846.1	Bacteria	Proteobacteria	443	
Sediminispirochaeta smaragdinae	WP_013256331.1	Bacteria	Spirochaetes	446	
Spirochaetales bacterium	MBI9105303.1	Bacteria	Spirochaetes	447	
Spirochaetales bacterium	MBN2627456.1	Bacteria	Spirochaetes	443	
Spirochaetes bacterium	RKX87856.1	Bacteria	Spirochaetes	443	
Treponema lecithinolyticum	WP_021686300.1	Bacteria	Spirochaetes	446	
Treponema primitia	WP_010255892.1	Bacteria	Spirochaetes	446	
Spirochaetales bacterium	HHA10855.1	Bacteria	Spirochaetes	412	
Spirochaetes bacterium Adurb.Bin110	OQB98325.1	Bacteria	Spirochaetes	453	
Acidobacteria bacterium	NLV31965.1	Bacteria	Acidobacteria	464	
Spirochaeta cellobiosiphila	WP_028974176.1	Bacteria	Spirochaetes	445	
Spirochaetia bacterium	MBL7005556.1	Bacteria	Spirochaetes	444	
unclassified Oceanispirochaeta	WP_114631117.1	Bacteria	Spirochaetes	446	
Salinispira pacifica	WP_024268780.1	Bacteria	Spirochaetes	446	
Spirochaetales bacterium	NCN05705.1	Bacteria	Spirochaetes	445	
Spirochaetales bacterium	NNM54587.1	Bacteria	Spirochaetes	444	
Spirochaetales bacterium	MBN1648674.1	Bacteria	Spirochaetes	442	
Spirochaetaceae bacterium	MBI9098259.1	Bacteria	Spirochaetes	438	
Sphaerochaeta sp.	TAH56144.1	Bacteria	Spirochaetes	439	
Spirochaetia bacterium	NCC64641.1	Bacteria	Spirochaetes	443	
Treponema sp. CETP13	OJF75634.1	Bacteria	Spirochaetes	442	
Spirochaetaceae bacterium	MBK5200914.1	Bacteria	Spirochaetes	441	
Sphaerochaeta coccoides	WP_013738790.1	Bacteria	Spirochaetes	444	
Spirochaetae bacterium HGW-Spirochaetae-4	PKL20180.1	Bacteria	Spirochaetes	444	
Sphaerochaetaceae bacterium	MBN2859002.1	Bacteria	Spirochaetes	432	
Bacteroidia bacterium	TFH38833.1	Bacteria	Bacteroidetes	442	
Bacteroidetes bacterium	HDR89148.1	Bacteria	Bacteroidetes	442	
Ignavibacteriales bacterium	MBI3786642.1	Bacteria	Ignavibacteriae	444	
Opitutaceae bacterium TAV4	RRJ94443.1	Bacteria	Verrucomicrobia	445	
Chitinispirillaceae bacterium	MBN2036071.1	Bacteria	Fibrobacteres	462	
Cyclobacteriaceae bacterium	MBL7852696.1	Bacteria	Bacteroidetes	439	
Azospira oryzae	PZR39501.1	Bacteria	Proteobacteria	442	
Soil – xym2 (unspecified)	AEG75766	Bacteria	Uncultured bacteri	442	Active XI in S. cerevisiae
Fulvivirga sp.1062	WP_225697917.1	Bacteria	Bacteroidetes	442	
Candidatus Marinimicrobia bacterium	MBF88841.1	Bacteria	Candidatus Marinir	444	

Candidatus Marinimicrobia bacterium	MAI88674.1	Bacteria	Candidatus Marinir	444	
Aliifodinibius sediminis	WP_142714288.1	Bacteria	Balneolaeota	442	
Balneola sp.	MAL17864.1	Bacteria	Balneolaeota	442	
Saprospiraceae bacterium	NNF33336.1	Bacteria	Bacteroidetes	437	
Saprospiraceae bacterium	MBK8515993.1	Bacteria	Bacteroidetes	439	
Bacteroidetes bacterium	RLD74399.1	Bacteria	Bacteroidetes	437	
Bacteroidetes bacterium	MBU0472981.1	Bacteria	Bacteroidetes	445	
Bacteroidia bacterium	MBT8221489.1	Bacteria	Bacteroidetes	442	
Membranicola marinus	WP_222581255.1	Bacteria	Bacteroidetes	442	
Balneola sp.	MAH86415.1	Bacteria	Balneolaeota	442	
Fulvivirga sp. 29W222	WP_202855150.1	Bacteria	Bacteroidetes	458	
Bacteroidetes bacterium	RMF24491.1	Bacteria	Bacteroidetes	448	
Saprospiraceae bacterium	MCB0653823.1	Bacteria	Bacteroidetes	448	
Saprospiraceae bacterium	MBX2870961.1	Bacteria	Bacteroidetes	452	
Bacteroidetes bacterium	MBR9922640.1	Bacteria	Bacteroidetes	441	
Fulvivirga sp. M361	WP_143397025.1	Bacteria	Bacteroidetes	442	
Lewinellaceae bacterium	MBK8426593.1	Bacteria	Bacteroidetes	445	
Bacteroidetes bacterium	MBI5916229.1	Bacteria	Bacteroidetes	453	
Saprospiraceae bacterium	MBK7790712.1	Bacteria	Bacteroidetes	441	
Bacteroidales bacterium	MBS3769338.1	Bacteria	Bacteroidetes	443	
Cyclobacteriaceae bacterium	MBS0001031.1	Bacteria	Bacteroidetes	437	
Lewinella sp. W8	MTB51209.1	Bacteria	Bacteroidetes	438	
Bacteroidetes Order II. Incertae sedis bacterium	MBT3448544.1	Bacteria	Bacteroidetes	439	
Rhodothermales bacterium	MBO6577226.1	Bacteria	Rhodothermaeota	437	
Fulvivirga lutimaris	WP_155186931.1	Bacteria	Bacteroidetes	447	
Saprospiraceae bacterium	NNE29972.1	Bacteria	Bacteroidetes	440	
uncultured bacterium	AOE07945.1	Bacteria	Environmental sam	441	
Rhodothermaeota bacterium MED-G19	PDH41433.1	Bacteria	Rhodothermaeota	443	
Cytophagales bacterium	HIF48435.1	Bacteria	Bacteroidetes	442	
Marivirga lumbricoides	WP_188462188.1	Bacteria	Bacteroidetes	442	
Bacteroidetes bacterium	MBU6169270.1	Bacteria	Bacteroidetes	445	
Saprospirales bacterium	MBK6931772.1	Bacteria	Bacteroidetes	453	
Flavobacteriaceae bacterium	UCE69242.1	Bacteria	Bacteroidetes	441	
Aquimarina celericrescens	MBQ0736376.1	Bacteria	Bacteroidetes	441	
Zhouia amylolytica	WP_074979729.1	Bacteria	Bacteroidetes	441	
Flavobacteriaceae bacterium	MBL6667032.1	Bacteria	Bacteroidetes	437	
Cytophagaceae bacterium	MBE16931.1	Bacteria	Bacteroidetes	441	
Ochrovirga pacifica	WP_010134855.1	Bacteria	Bacteroidetes	443	
Rhodospirillaceae bacterium TMED63	RPF97854.1	Bacteria	Proteobacteria	438	Selected XI to express
Flavobacteriaceae bacterium	MAU64033.1	Bacteria	Bacteroidetes	442	
Flavobacteriaceae bacterium	MBK78829.1	Bacteria	Bacteroidetes	441	
uncultured bacterium	AOE10719.1	Bacteria	Environmental sam	441	
Algibacillus agarilyticus	WP_111978035.1	Bacteria	Proteobacteria	444	
Cytophagales bacterium	HBH25142.1	Bacteria	Bacteroidetes	438	
Zunongwangia atlantica 22II14-10F7	ORL44102.1	Bacteria	Bacteroidetes	455	
Robertkochia solimangrovi	WP_143955657.1	Bacteria	Bacteroidetes	441	
Chryseobacterium sp. Leaf405	WP_055983457.1	Bacteria	Bacteroidetes	443	
Bacteroidetes bacterium	MCB0843808.1	Bacteria	Bacteroidetes	450	
Chitinophagaceae bacterium	MBL7699742.1	Bacteria	Bacteroidetes	443	
Aquirufa rosea	WP_129026770.1	Bacteria	Bacteroidetes	442	
Segetibacter sp.	MBA4139662.1	Bacteria	Bacteroidetes	442	
Sphingobacteriales bacterium	MBN8877712.1	Bacteria	Bacteroidetes	442	
Saprospiraceae bacterium	MBC7886072.1	Bacteria	Bacteroidetes	442	
Chitinophaga costaii	SCC15653.1	Bacteria	Bacteroidetes	463	
Elizabethkingia occulta	WP_078772168.1	Bacteria	Bacteroidetes	442	
Cyclobacteriaceae bacterium	MBY0433018.1	Bacteria	Bacteroidetes	402	
Hymenobacter sp.	RZK16911.1	Bacteria	Bacteroidetes	438	
Saprospiraceae bacterium	MBL7816515.1	Bacteria	Bacteroidetes	437	
Bacteroidetes bacterium	TAF72132.1	Bacteria	Bacteroidetes	442	
Bacteroidales bacterium	NOR34363.1	Bacteria	Bacteroidetes	443	
Cytophagaceae bacterium	MBX9852919.1	Bacteria	Bacteroidetes	452	
Rhodocytophaga rosea	WP_162446076.1	Bacteria	Bacteroidetes	445	
Cytophagales bacterium	TAH21206.1	Bacteria	Bacteroidetes	445	
Fibrisoma limi	WP_009284593.1	Bacteria	Bacteroidetes	448	
Cytophagales bacterium TFI 002	SOE22188.1	Bacteria	Bacteroidetes	446	
Emticicia aquatilis	GGD77028.1	Bacteria	Bacteroidetes	467	
Lentisphaerae bacterium	MBM4154822.1	Bacteria	Lentisphaerae	442	
Chthoniobacterales bacterium	HET87156.1	Bacteria	Verrucomicrobia	456	
Algisphaera agarilytica	WP_184678473.1	Bacteria	Planctomycetes	445	
Pelagicoccus enzymogenes	WP_191617329.1	Bacteria	Verrucomicrobia	443	

Opitutales bacterium	MBN2068373.1	Bacteria	Verrucomicrobia	445	
Gammaproteobacteria bacterium	MBN1378635.1	Bacteria	Proteobacteria	443	
Agaribacterium haliotis	WP_096086225.1	Bacteria	Proteobacteria	443	
Rubrivirga marina	WP_095508650.1	Bacteria	Rhodothermaeota	443	
Teredinibacter turnerae	WP_015820877.1	Bacteria	Proteobacteria	444	
Soil – xym1 (unspecified)	AEG75765	Bacteria	Environmental sam	443	Active XI in S. cerevisiae
Sorangium cellulosum	WP_020464968	Bacteria	Proteobacteria	443	Active XI in S. cerevisiae
Saprospiraceae bacterium	MBX2816973.1	Bacteria	Bacteroidetes	442	
Flammeovirga pacifica	WP_044227526.1	Bacteria	Bacteroidetes	449	
Bacteroidetes bacterium	RLD85127.1	Bacteria	Bacteroidetes	443	
Saprospiraceae bacterium	MCB0647565.1	Bacteria	Bacteroidetes	442	
Saprospiraceae bacterium	MBP6793696.1	Bacteria	Bacteroidetes	444	
Ignavibacteria bacterium	NLT51376.1	Bacteria	Bacteroidetes	444	
Bacteroidales bacterium	MBN1116951.1	Bacteria	Bacteroidetes	442	
Rhodothermia bacterium	MBN8588852.1	Bacteria	Bacteroidetes	436	
Cyclobacterium amurskyense	AKP54227.1	Bacteria	Bacteroidetes	460	
unclassified Alistipes	WP_216645973.1	Bacteria	Bacteroidetes	437	
uncultured symbiotic protist of Reticulitermes sp	BAX90016.1	Eukaryotes	Eukaryotes	438	
Bacteroidales bacterium	MBK7175233.1	Bacteria	Bacteroidetes	441	
Marinilabiliaceae bacterium	MBI9065087.1	Bacteria	Bacteroidetes	441	
Gaetbulibacter sp. 4G1	PIA82368.1	Bacteria	Bacteroidetes	438	
Bacteroidales bacterium	PCH71389.1	Bacteria	Bacteroidetes	441	
Bacteroidetes bacterium	HDZ40767.1	Bacteria	Bacteroidetes	432	
Bacteroidales bacterium	HHU99059.1	Bacteria	Bacteroidetes	457	
Bacteroidales bacterium	HCI54904.1	Bacteria	Bacteroidetes	442	
Tangfeifania diversioriginum	WP_073165153.1	Bacteria	Bacteroidetes	442	
Prolixibacteraceae bacterium	MBK5202536.1	Bacteria	Bacteroidetes	443	
Bacteroidetes bacterium	NLV19642.1	Bacteria	Bacteroidetes	445	
Sphingobacteriia bacterium	NCC72530.1	Bacteria	Bacteroidetes	441	
Marinilabiliales bacterium	PLW99285.1	Bacteria	Bacteroidetes	437	
Bacteroidetes bacterium GWE2 42 24	OFY10649.1	Bacteria	Bacteroidetes	442	
Parabacteroides sp. D13	EEU51936.1	Bacteria	Bacteroidetes	464	
Flavobacteriaceae bacterium 3519-10	ACU08186.1	Bacteria	Bacteroidetes	461	
Candidatus Sphingobacterium stercoripullorum	HIX53560.1	Bacteria	Bacteroidetes	440	
Bacteroidales bacterium	MBQ6911716.1	Bacteria	Bacteroidetes	440	
Hallella seregens	WP_027952460	Bacteria	Bacteroidetes	439	Selected XI to express
Bacteroidales bacterium	MBR1785201.1	Bacteria	Bacteroidetes	439	
Bovine rumen (unspecified)	AEL74969	Bacteria	Environmental sam	439	Active XI in S. cerevisiae
Prevotella ruminicola	AGL34957	Bacteria	Bacteroidetes	439	Active XI in S. cerevisiae
Bacteroidales bacterium 45-6	OJU49823.1	Bacteria	Bacteroidetes	439	
bacterium J10(2018)	RLT77382	Bacteria	Unclassified Bacter	438	Selected XI to express
Bacteroides vulgatus	ABR41556	Bacteria	Bacteroidetes	438	Active XI in S. cerevisiae
Bacteroides stercoris	KGQ13041.1	Eukaryotes	Eukaryotes	438	Active XI in S. cerevisiae
Neocallimastix californiae	ORY81692	Eukaryotes	Eukaryotes	449	Selected XI to express
Anaeromyces robustus	ORX78688	Eukaryotes	Eukaryotes	415	Selected XI to express
Piromyces sp.	AWI66859.1	Eukaryotes	Eukaryotes	461	
Piromyces sp. E2	CAB76571.1	Eukaryotes	Eukaryotes	437	Active XI in S. cerevisiae
Orpinomyces sp. ukk1	ACA65427	Eukaryotes	Eukaryotes	437	Active XI in S. cerevisiae

Superkingdom	Phylum	Number	Total number
Bacteria	Proteobacteria	180	829
	Firmicutes	135	
	Bacteroidetes	100	
	Actinobacteria	85	
	Planctomycetes	79	
	Spirochaetes	42	
	Acidobacteria	32	
	Chloroflexi	26	
	Verrucomicrobia	25	
	Unclassified Bacteria	19	
	Thermotogae	14	
	Lentisphaerae	9	
	Candidatus Marinimicrobia	8	
	Tenericutes	8	
	Armatimonadetes	7	
	Deinococcus-Thermus	6	
	Environmental samples	6	
	Balneolaeota	4	
	Candidatus Hydrogenedentes	4	
	Fibrobacteres	4	
	Calditrichaeota	3	
	Fusobacteria	3	
	Gemmatimonadetes	3	
	Ignavibacteriae	3	
	Rhodothermaeota	3	
	Candidatus Melainabacteria	2	
	Candidatus Poribacteria	2	
	Abditibacteriota	1	
	candidate division WOR-3	1	
	Candidatus Binatota	1	
	Candidatus Bipolaricaulota	1	
	Candidatus Cloacimonetes	1	
	Candidatus Cryosericota	1	
	Candidatus Dormibacteraeota	1	
	Candidatus Handelsmanbacteria	1	
	Candidatus Omnitrophica	1	
	Candidatus Peregrinibacteria	1	
	Candidatus Sumerlaeota	1	
	Chlamydiae	1	
	Cyanobacteria	1	
	Elusimicrobia	1	
	Kiritimatiellaeota	1	
	Lentisphaeria	1	
	Synergistetes	1	
Eukaryotes	Eukaryotes	48	48
Archaea	Archaea	6	6
